# Rotational Spectroscopy Pinpoints the Tetrahydrate as the Onset of Water Self-Aggregation in Sevoflurane Hydration

**DOI:** 10.1021/acs.jpclett.5c01767

**Published:** 2025-08-04

**Authors:** Amanda L. Steber, Luca Evangelisti, Simon Lobsiger, Zbigniew Kisiel, Brooks H. Pate, Alberto Lesarri, Cristóbal Pérez

**Affiliations:** † Departamento de Química Física y Química Inorgánica, Facultad de Ciencias-I.U. CINQUIMA, 16782 Universidad de Valladolid, E-47011 Valladolid, Spain; ‡ Dipartimento di Chimica “G. Ciamician”, 9296 Università di Bologna, 40126 Bologna, Italy; ¶ Department of Chemistry, University of Virginia, Charlottesville, Virginia 22904-4319, United States; § 86906 Federal Institute of Metrology METAS, Lindenweg 50, 3003 Bern-Wabern, Switzerland; ∥ Institute of Physics, 2358 Polish Academy of Sciences, 02-668 Warszawa, Poland

## Abstract

Characterizing the interactions between water and volatile anesthetics at a molecular level is crucial for understanding their mechanisms of action. We employed broadband molecular rotational spectroscopy (CP-FTMW) and extensive isotopic substitution experiments to generate and characterize the stepwise addition of up to four water molecules to the volatile anesthetic sevoflurane, a flexible molecule with multiple binding sites. The substantial amount of isotopic data enabled the conclusive derivation of accurate structural information. The observed structures contain the most stable conformer of the previously identified monomer, with water clusters favorably interacting with the molecule to form an open chain with up to three water molecules. Notably, two isomers were detected for the tetrahydrate, which exhibit a cyclic structure with either a clockwise or anticlockwise orientation, resembling that of the pure water tetramer. The four-water marks a transition where water–water interactions dominate over direct sevoflurane–water interactions driving the assembly of the water network.

## Graphical abstract

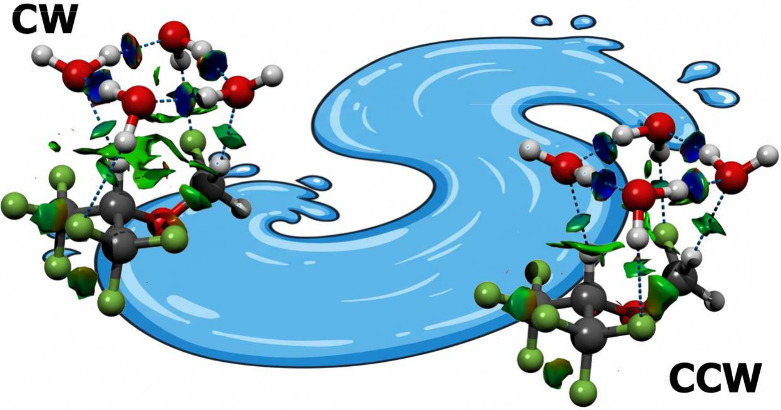

Anesthetic binding is not a simple, direct interaction. The interaction between general anesthetics and ligand- or voltage-gated ion channels exemplifies the complexity of molecular docking, a process governed by a delicate balance of weak, noncovalent interactions.^1^ These binding events, often exhibiting a prototypical character, represent localized interactions within a dynamic molecular environment. Biological processes invariably occur in solution, where active anesthetic molecules and biological receptors interact with surrounding water molecules. Water molecules, forming a dynamic hydration shell, play a critical role by mediating interactions through hydrogen bonds (HB), bridging anesthetics and channel residues, and influencing binding affinity. The hydrophobic nature of many anesthetics drives them toward nonpolar regions within the channel, with water expulsion contributing to binding entropy.^2^ However, interfacial water molecules also structure the binding pocket and regulate anesthetic access. Additionally, water molecules participate in long-range communication within the protein, forming hydrogen-bonded networks that propagate conformational changes. They can also directly compete with anesthetics for binding sites. A deeper investigation of these water-mediated interactions, using techniques like molecular dynamics simulations and high-resolution structural studies, is thus essential for a more comprehensive understanding of anesthetic mechanisms.

Understanding the preferred structures of solvated organic molecules and the role of solvation in their reactivity is crucial. Modeling solute–water interactions at the molecular level — where the solute’s structure is explicitly defined by the number and arrangement of solvent molecules — bridges gas-phase and solution-phase behaviors. While solution-phase complexity necessitates sophisticated computational approaches, gas-phase studies provide a unique opportunity to isolate and analyze these interactions. Under controlled conditions, water molecules can be sequentially incorporated into a microsolvated molecule, effectively constructing the first solvation shell. Consequently, many structural and dynamic studies are conducted in the gas phase, often utilizing supersonic jet expansions. When coupled with high-resolution spectroscopic techniques, in particular Chirped-Pulsed Fourier Transform Microwave Spectroscopy (CP- FTMW),^3,4^ this approach yields precise structural information on weakly bound complexes, offering valuable insights into the initial steps of solvation.^5–12^

Sevoflurane (CH_2_FOCH­(CF_3_)_2_, C_4_H_3_F_7_O, SEV), a fluorinated ether, is among the most widely utilized inhalational anesthetics for both the induction and maintenance of general anesthesia. Previous investigations employing rotational spectroscopy have been conducted to elucidate the intrinsic molecular properties of the sevoflurane monomer,^13^ dimer,^14^ trimer,^15^ and its complex with benzene.^16^ These studies revealed the preferred structures of the molecular aggregates while challenging the limits of rotational spectroscopy due to their large molecular mass.

In this study, we examine the preferred structures and HB networks when the volatile anesthetic sevoflurane is sequentially surrounded by up to four water molecules. Due to the dual nature of water as either a donor or an acceptor, SEV’s structure offers multiple hydrogen-bonding sites where water molecules can be linked. For instance, SEV can participate in proton-acceptor hydrogen bonds through the oxygen lone pairs and/or proton-donor through its fluorine-activated C–H bonds. Using extensive isotopic substitution in both, natural and enriched samples, we were able to fully determine the structures of SEV with up to three water molecules and the HB networks that are established. Our results confirm that, on complexation with up to three water molecules, the perfluoro isopropyl hydrogen (donor) atom acts as the main anchoring point to establish a strong HB with a water molecule and that the fluoromethoxy group (acceptor) closes the cycle regardless of the number of water molecules that are sequentially inserted in the structure or the cluster. For the four water complexes, two isomers with similar experimental populations were found. These two complexes are structurally similar and only differ in the clockwise or counterclockwise orientation of the HB network of the cyclic water tetramer.

The rotational spectrum of SEV–water was recorded in the CP-FTMW spectrometer at the University of Virginia operating in the 2–8 GHz regime. First, a mixture of 0.2% sevoflurane in Ne was flown over an external reservoir containing distilled water and injected into the vacuum chamber through five solenoid valves (Parker, Series 9, nozzle diameter 0.8 mm) at a backing pressure of 1.5 atm. A more detailed description of the operating principle has been reported elsewhere.^3,4^ The final scan consisted of one million averaged free induction decays (FID), as shown in Figure 1. The predominant spectrum was identified as belonging to the previously studied sevoflurane monomer^13^ at a signal-to-noise ratio (SNR) of roughly 6500:1. After all the monomer transitions as well as the pure water clusters spectra previously reported^17,18^ were removed, three distinct rotational spectra became discernible. The next strongest spectrum (in red in Figure 1) exhibits a SNR of about 4500:1. In decreasing order of intensity, the other two spectra appeared at a SNR of 600:1 and 500:1, both are shown in blue and green respectively in Figure 1. All three have a sufficient SNR to observe their singly substituted ^13^C species in natural abundance as well as the ^18^O isotopologues for the strongest spectrum. Transitions of a-, b-, and c-type were observed in all three spectra. Their corresponding rotational parameters are reported in Table 1 while the transitions are tabulated in the Supporting Information. Measured transition frequencies in all spectra were fit using the Watson’s A-reduced Hamiltonian in the I^*r*^ representation as implemented in the SPFIT/SPCAT program suite.^19^ The statistically controlled single- substitution, whether in natural abundance or using enriched samples, induces relatively minor changes in the system’s moments of inertia. These variations are, nonetheless, detectable due to the high resolution and sensitivity of current broadband spectrometers. The Kraitchman substitution method^20^ utilizes these small changes to determine the magnitude of each positional coordinate of the substituted atom within the principal-axis system of the parent isotopic species. Therefore, in order to obtain such isotopic data for the observed water-containing complexes and assuming that the larger species belonged to a three-water cluster, we performed a second experiment using a spiked sample of water with a 3:1 mixture H_2_^16^O:H_2_^18^O (500 k FIDs) to statistically favor the insertion of a single H_2_^18^O molecule in the structure of the three-water complex without significantly decreasing the signal of the smaller clusters. Without any input from theory, we employed automatic fitting routines^21^ aiming to find the corresponding singly substituted species. The used AUTOFIT program only requires, as an initial guess, a set of rotational constants and the magnitude of dipole moment components in the principal system, both extracted from the assigned normal species spectra. Hence, our methodology consisted of searches over the necessarily red-shifted frequency ranges as the mass of the complex increases upon substitution with the heavier oxygen isotope. Following this procedure, a total of 6 additional spectra were identified. A small portion of this measurement is also shown in Figure 1 illustrating the effect of the three distinctive H_2_^18^O insertions. Subsequently, they were easily correlated with their corresponding parent species based on their rotational constants. Their rotational parameters are reported in Table S1 along with the observed frequencies. The H_2_^18^O doped sample enabled the identification of three distinct groups of new spectra, each correlating with the single-substitution of water molecules within the one-, two-, and three-water complexes, respectively. This observation provides strong evidence for the precise number of water molecules comprising each cluster.

**Figure 1:**
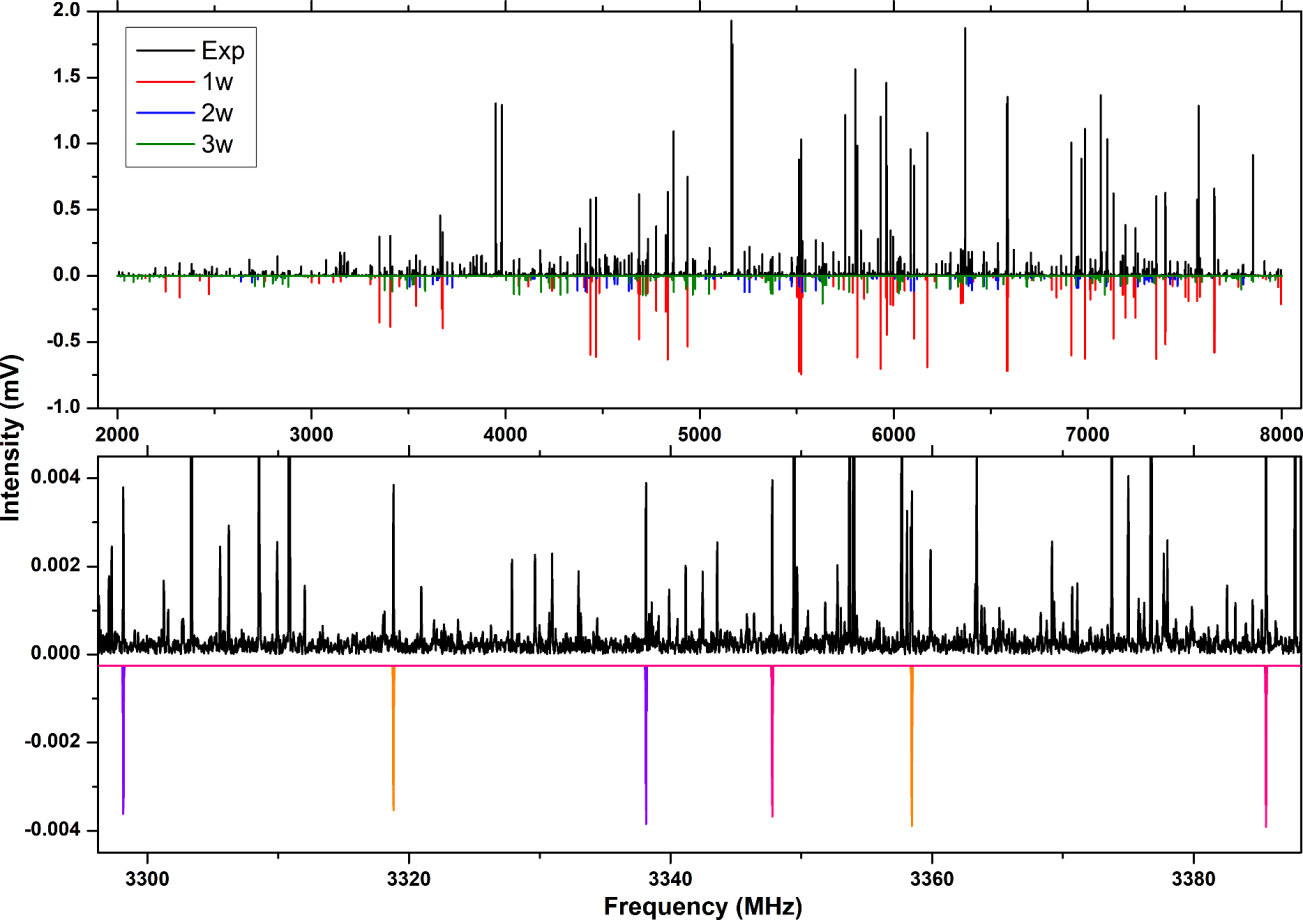
2–8 GHz spectrum (1 Million FIDs) of SEV–water, with 1.5 K simulations (top panel) of SEV–(H_2_O) (red), SEV–(H_2_O)_2_ (blue), and SEV–(H_2_O)_3_ (green) based on experimentally determined rotational parameters. The three ^18^O isotopologues of SEV–(H_2_O)_3_ are also shown (bottom panel) with two transitions marked for each (5_15_-4_14_ (lower frequency) and 5_05_-4_04_ (higher frequency)). The isotopologues are well resolved in frequency space, where the substitutions furthest from the center of mass are the most red-shifted from the parent transition. This spectrum comprises 500k FIDs using an H_2_^18^O enriched water sample.

**Table 1:** Experimentally Determined Rotational Parameters for the SEV–(H_2_O)_1–4_ Complexes^a^

	SEV–(H_2_O)	SEV–(H_2_O)_2_	SEV–(H_2_O)_3_	SEV–(H_2_O)_4_ CW	SEV–(H_2_O)_4_ CCW
*A* (MHz)	822.23653(44)	737.85016(159)	612.47396(75)	492.54315(10)	504.68006(14)
*B* (MHz)	645.85854(40)	481.99047(75)	372.429002(303)	368.83715(10)	359.21357(12)
*C* (MHz)	533.76059(47)	425.93344(55)	328.729677(305)	328.372229(94)	316.06881(10)
Δ_*J*_ (kHz)	0.0833(46)	0.1448(54)	0.09496(130)	0.09307(46)	0.12512(50)
Δ_*JK*_ (kHz)	0.5733(112)	–0.3736(270)	–0.1687(39)	–0.1478(20)	–0.1768(21)
Δ_*K*_ (kHz)	–0.4339(113)	0.564(68)	0.4302(297)	0.1705(21)	0.1955(26)
δ_*J*_ (kHz)	–0.01330(237)	–0.0315(33)	–9.31(92)	–0.00372(27)	–0.00687(29)
δ_*K*_ (kHz)	9.70(70)	-	–1.02(59)	0.0607(43)	0.1109(44)
σ (kHz)	4.8	4.3	10.9	3.5	4.2
*N*	193	90	206	199	180

a *A*, *B*, and *C* are the rotational constants. *Δ*_*J*_, *Δ*_*JK*_, *Δ*_*K*_, *δ*_*J*_, *δ*_*K*_ are the centrifugal distortion constants in the Watson’s A-reduction. *σ* is the rms deviation of the fit, and *N* is the number of transitions in the fit.

Two approaches were utilized for the structural analysis. First, we used the Kraitchman method^20^ that allows us to obtain the so-called substitution structure, *r*_*s*_. The main results of this method are shown in Figure 2, and the complete analysis is reported in the SM. Coordinates resulting from the *r*_*s*_ analysis are absolute values that require sign assignment from additional information. Therefore, we performed an exhaustive computational search to obtain reliable candidate structures for each cluster size.

**Figure 2:**
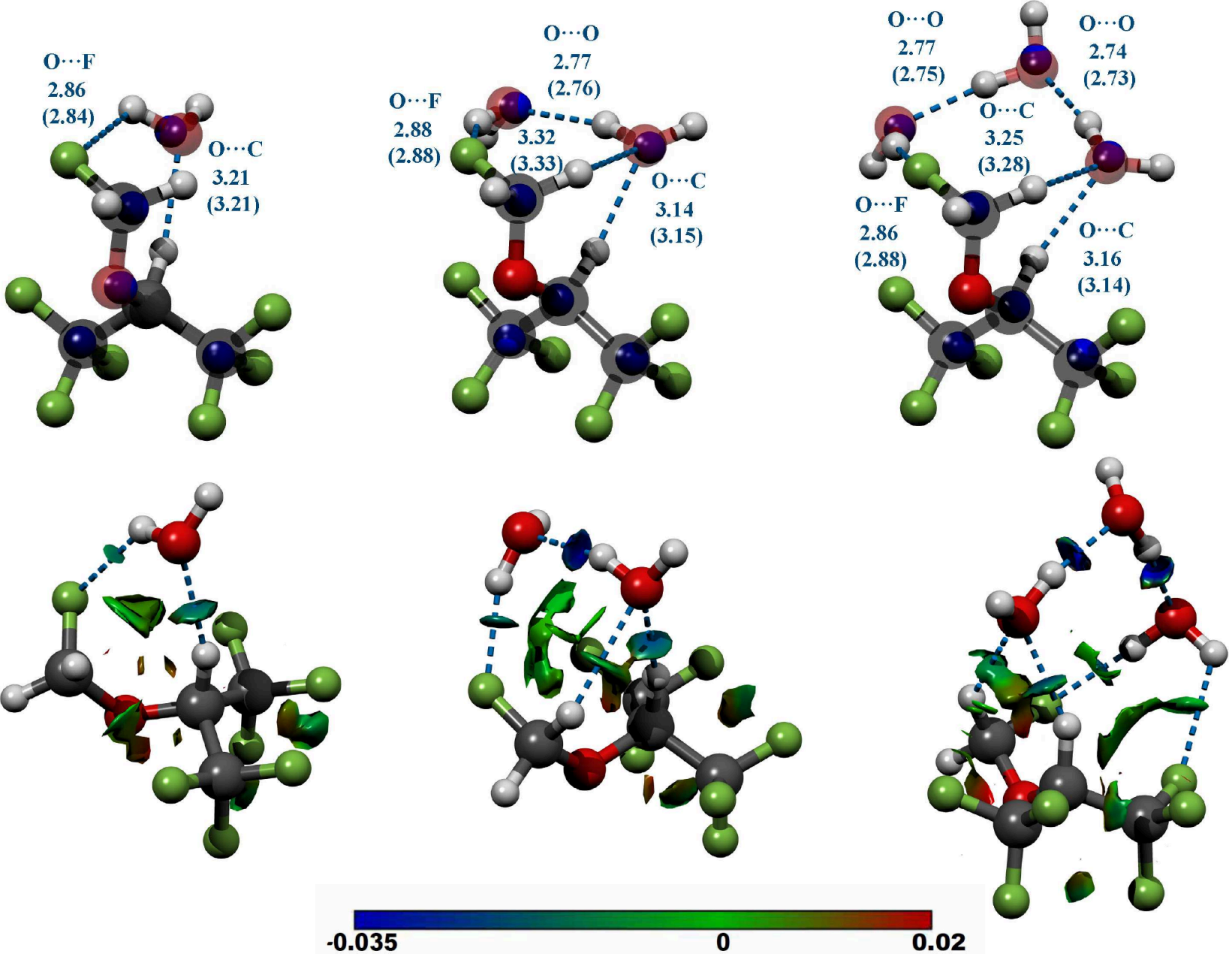
Top row: Experimental structures of the observed sevoflurane–water clusters with up to three water molecules obtained from the *r*_0_ least-squares fits to 18, 21, and 24 determined moments of inertia of the parent, ^13^C, and ^18^O isotopologues for the one-, two-, and three-water complexes, respectively. The relevant experimental structural parameters are compared with the results from quantum chemical calculations at the B3LYP-D3­(BJ)/aug-cc-pVTZ level of theory (in brackets). The blue spheres represent the experimental atom positions obtained using the Kraitchman method from single isotopic substitutions. All distances are in angstroms. The experimental uncertainties are reported in the Supporting Information for simplicity. Bottom row: the NCI analysis mapping the location and strength of intermolecular interactions. Interactions range from attractive, strong HB in blue to repulsive interactions in red based on the sign of (λ_2_)*ρ. λ*_2_ is the second eigenvalue of the electron density Hessian and ρ is the electron density in atomic units.

An initial exploration was conducted using the Conformer-Rotamer Ensemble Sampling Tool (CREST)^22^ to evaluate the potential energy surface (PES) of each cluster size. The resulting set of initial structures was then optimized using quantum-chemical calculations, specifically DFT methods. We initially selected ωB97X-D3/6–31+G­(d) as implemented in ORCA,^23,24^ balancing speed and accuracy. The obtained structures were subsequently reoptimized at B3LYP-D3­(BJ)/aug-cc-pVTZ with an energy cutoff of 2 kJ/mol, including vibrational frequency calculations to confirm they were real minima and to obtain zero-point vibrational corrections. Additionally, we performed optimizations and frequency calculations at the MP2/cc-pVTZ level using the resolution-of-identity (RI) approximation with the cc-pVTZ/C auxiliary basis set. This level of theory provided satisfactory results in previous studies.^5,8^ The results of this search are reported in the SM. The identified candidate structures enabled a comprehensive structural analysis by leveraging all available isotopic information. Often, the preferred analysis involves performing a least-squares fit of a theoretical structure to the experimental moments of inertia from the isotopic species. This approach allows for accurately assessing structural parameters that would otherwise be unobtainable with limited isotopic information.^25,26^ In this study, we chose the effective structure (*r*_0_), which neglects ro-vibrational contributions but has been shown to provide reliable results for structural studies of other water clusters.^4,5,8,17,18^ Relevant parameters from this analysis, in particular the O···O, O···F, and O···C distances that characterize the heavy-atom backbone of the water cluster and its distances from sevoflurane are presented in Figure 2. We performed structural fits using Density Functional Theory (DFT) and ab initio structures as initial models, yielding comparable results. In all *r*_0_ structural fits, only the minimal number of heavy atom intermolecular parameters was fitted, ensuring fit stability and rapid convergence. The fits incorporated all available rotational constants, including those for isotopic substitution within the sevoflurane unit. While the isolated monomer adopts a single conformation featuring a gauche fluoromethoxy group and a nearly symmetric alignment of the isopropyl group relative to the ether plane, computations indicated significant changes in the C–C–O–C and F–C–O–C twists (dihedral angles) in the heavy atom backbone of sevoflurane on its successive hydration. Some of these changes exceeded 15°, but could not be fitted unambiguously given the lack of isotopic information concerning the fluorine atoms, so they were assumed from computation. A comprehensive description of the analysis is provided in the SM. Additionally, to characterize the noncovalent interactions within the system, a Noncovalent Interaction (NCI) analysis^27^ was performed using the computational package Multiwfm.^28,29^ The results derived from this computational method, which provides a qualitative, three-dimensional representation of interaction regions, are also presented in Figure 2.

From the *r*_0_ structural analysis, the stabilization of the sevoflurane cluster with one water molecule is achieved through two HB interactions. The water molecule simultaneously functions as both a donor and an acceptor, interacting with the fluoromethoxy and hexafluoroisopropyl hydrogen atoms, with O···F distance of 2.86Å and O···C distance of 3.21 Å.

In the two-water complex, the second water molecule is positioned to form a cyclic arrangement with the first water molecule and the fluoromethoxy group, engaging with one of the methylene hydrogen atoms. Additionally, the second water molecule acts as an HB donor to the fluorine atom. This cyclic structure is further anchored to the monomer via a directional interaction with the isopropylic hydrogen atom, exhibiting an O···C distance of 3.14 Å. The O···O distance within this complex is 2.77 Å, which is significantly shorter than the 2.98(4) Å observed in the isolated water dimer.^30^ This indicates cooperative effects influenced by the local environment of the dimer unit, and is consistent with similar determinations, such as for ethyl carbamate–(H_2_O)_2_, *r*_0_(O···O) = 2.763(5) Å,^31^ or HCl–(H_2_O)_2_, *r*_0_(O···O) =2 .809(1) Å.^32^

The three-water complex also exhibits a cyclic HB network maintaining interactions with the fluoromethoxy group. The third water monomer is integrated into the cycle, functioning as both a donor and acceptor. The O···O distances in this configuration are 2.77 Å and 2.74 Å, while the O···C distance of 3.16 Å remains essentially unchanged. Notably, DFT predicted the global minimum to be the observed open-chain arrangement of the three water units, incorporated into a cycle completed by O···FC and CH···O interactions. On the other hand, MP2 computations also predicted a slightly more stable cyclic water attached to the fluoromethoxy fluorine on one corner. Despite exhaustive searches, such a cyclic water-based trimer structure could not be observed. Furthermore, isotopic substitution experiments conclusively distinguish the open chain structure from a cyclic water arrangement. This discrepancy can be rationalized by the tendency of MP2 calculations to overestimate water–water interactions in homodromic water rings, while dispersion-corrected methods more accurately account for weaker interactions that significantly influence the overall configuration of the observed hydrogen bond networks.^33,34^

To further complete the spectral analysis and given the significant number of remaining transitions, we conducted a comprehensive exploration of the PES of the four-water complex utilizing the same procedure described above. These computational investigations revealed the existence of two nearly isoenergetic isomers (differing by 0.69 kJ/mol, 0.85 kJ/mol for the electronic and zero-point corrected energies, respectively). The results from this search are reported in Table S4. The primary distinction between these isomers lies in the orientation of the hydrogen bond network within the cyclic water tetramer, which can be oriented either in a clockwise (CW), or counterclockwise (CCW) direction as shown in Figure 3. Both isomers exhibit somewhat similar rotational constants and dipole moments. However, the assignment of these isomers is conclusively determined, particularly through the use of MP2-calculated rotational constants. It is noteworthy that in the four-water complex, the water cycle does not interact with the fluoromethoxy group in the same manner as observed in the smaller clusters. Instead, the pure water cluster arrangement predominates and is positioned atop the SEV monomer. This cluster size appears to be the transition point where water–water interactions dominate, thereby altering the interaction between water and the anesthetic molecule.

**Figure 3:**
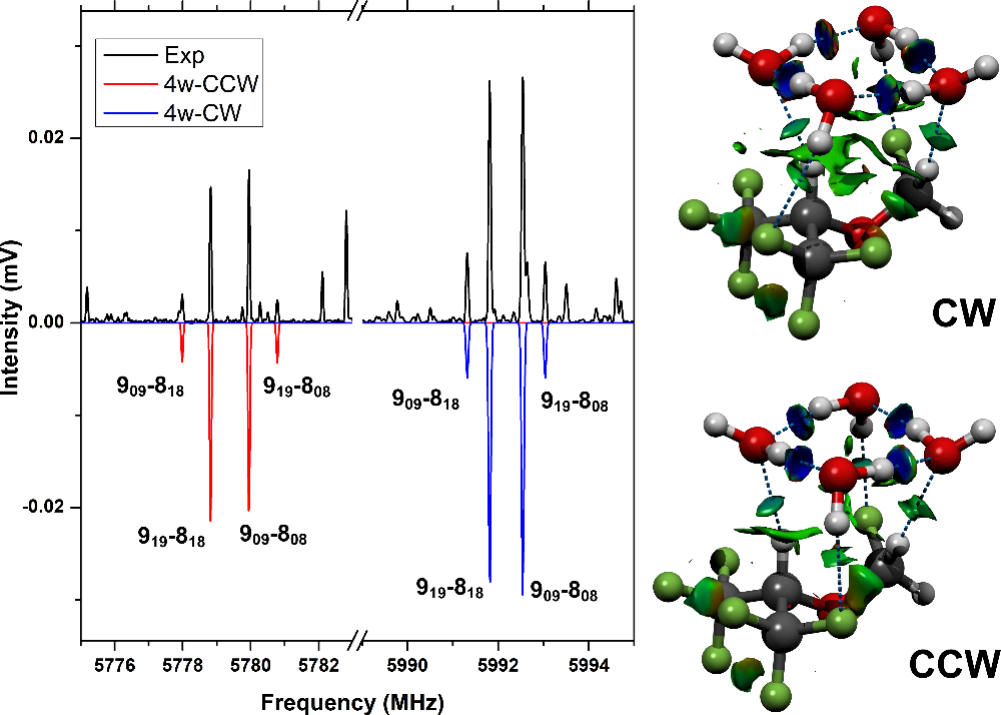
Structures of the observed four-water clusters on the right. The two isomers only differ on the clockwise (CW) or counterclockwise (CCW) orientation of the hydrogen bond network of the cyclic water tetramer. The NCI analysis is also displayed. Two small portions of the rotational spectrum containing characteristic a-b quartets are shown on the left-hand side for both isomers. The rotational levels for each transition are labeled using the standard asymmetric top notation, *J*_*K*_*a*_ *K*_*c*__. Here, *J* represents the total angular momentum quantum number, while *K*_*a*_ and *K*_*c*_ are pseudoquantum numbers indicating the projection of the angular momentum onto the molecule’s principal axes — the *a*-axis and *c*-axis — corresponding to the prolate and oblate symmetric top limits, respectively. The simulations (1.5 K) in negative scale are based on the rotational parameters of Table 1.

Additionally, a relative population analysis, based on the signal intensity and the predicted dipole moments, indicates a population ratio of 1:0.75 between the two isomers. To more accurately examine the energy difference between these two isomers, we performed DLPNO–CCSD­(T) computations (domain-based local pair-natural orbital coupled cluster perturbative triple-excitations method with the cc-pVTZ basis set and resolution- of-identity (RIJCOSX) approximation as implemented in ORCA^23,24^ ). These calculations rendered an energy difference of 0.62 kJ/mol which corresponds to a 0.77 Boltzman population ratio at the 298.15 K expansion nozzle temperature, in good agreement with our experimentally determined population ratio.

In conclusion, the rotational spectrum of SEV–water clusters was successfully recorded and analyzed, revealing detailed structural insights into clusters with up to four water molecules. Isotopic substitution experiments using H_2_^18^O confirmed the cluster compositions and provided precise structural information from the slight changes in the moments of inertia upon isotopic labeling. This enabled the determination of effective structures (*r*_0_) through least-squares fits to experimental data. The one-water cluster forms two hydrogen bonds, with the water acting as both donor and acceptor, while the two-water cluster adopts a cyclic structure with enhanced hydrogen bonding and shorter O···O distances than the isolated water dimer, indicating cooperative interactions. The three-water complex maintains this cyclic pattern, incorporating the third monomer seamlessly into the hydrogen bond network. Interestingly, computational results revealed a discrepancy between DFT and MP2 predictions, with DFT accurately predicting the observed open-chain structure, while MP2 favored a cyclic water trimer. This deviation was attributed to MP2’s tendency to overestimate water–water interactions in cyclic systems. For the four-water complex, experimental and computational exploration uncovered two nearly isoenergetic isomers differing only in the hydrogen bond network’s orientation, with experimental population ratios aligning with high-level DLPNO–CCSD­(T) calculations. Unlike smaller clusters, the four-water complex forms a self-sustained water cycle decoupled from the fluoromethoxy group, marking a transition where water–water interactions dominate over direct SEV–water interactions taking part in the water network. The NCI plots in Figures 2 and 3 underline these conclusions by providing a useful way of differentiating between strong and weak contacts between constituent molecules in the four SEV–water clusters. These findings provide a comprehensive view of SEV–water cluster structures, highlighting the evolving balance between water–water and water-anesthetic interactions as cluster size increases.

## Supplementary Material



## Notes

Structures from quantum-chemical calculations. Experimental rotational parameters. Substitution *r*_*s*_ and effective *r*_0_ structures. Tables of transition frequencies. NCI plots for the observed clusters (PDF)
