# Supplementary material for: Rotational Spectroscopy Pinpoints the Tetrahydrate as the Onset of Water Self-Aggregation in Sevoflurane Hydration
Source: J Phys Chem Lett. 2025 Aug 4;16(32):8209–15. doi: 10.1021/acs.jpclett.5c01767 (PMC12584135; doi:10.1021/acs.jpclett.5c01767)

Supplementary Material

Rotational Spectroscopy Pinpoints the Tetrahydrate as the Onset of Water Self-aggregation in Sevoflurane Hydration

Amanda L. Steber,[†] Luca Evangelisti,[‡] Simon Lobsiger,[¶,§] Zbigniew Kisiel,[‖] Brooks H. Pate,[¶] Alberto Lesarri,[*,†] and Cristóbal Pérez[*,†]

†Departamento de Química Física y Química Inorgánica, Facultad de Ciencias-I.U. CINQUIMA, Universidad de Valladolid, E-47011 Valladolid, Spain
‡Dipartimento di Chimica "G. Ciamician",Universita di Bologna, 40126 Bologna, Italy
¶Department of Chemistry, University of Virginia, Charlottesville, Virginia 22904-4319, USA
§Federal Institute of Metrology METAS, Lindenweg 50, 3003 Bern-Wabern, Switzerland
‖Institute of Physics, Polish Academy of Sciences, 02-668 Warszawa, Poland

E-mail: alberto.lesarri@uva.es; cristobal.perez@uva.es

Table of Contents

1. Computational Results

Table S1: Calculated spectroscopic parameters of SEV-(H_2O) cluster.

Table S2: Calculated spectroscopic parameters of SEV-(H_2O)$_2$ cluster.

Table S3: Calculated spectroscopic parameters of SEV-(H_2O)$_3$ cluster.

Table S4: Calculated spectroscopic parameters of SEV-(H_2O)$_4$ cluster.

2. Experimental Results

2.1 Spectroscopic Parameters

Table S5: Spectroscopic parameters for SEV- (H_2O) cluster and its isotopologues.

Table S6: Experimental structures from r_s and r_0 methods for the SEV-H_2O cluster.

Table S7: Spectroscopic parameters for SEV-(H_2O)$_2$ cluster and its isotopologues.

Table S8: Experimental structures from r_s and r_0 methods for the SEV-(H_2O)$_2$ cluster.

Table S9: Spectroscopic parameters for SEV-(H_2O)$_3$ cluster and its isotopologues.

Table S10: Experimental structures from r_s and r_0 methods for the SEV-(H_2O)$_3$ cluster.

Table S11: Spectroscopic parameters for SEV-(H_2O)$_4$ clusters.

Table S12: Observed rotational transitions (Obs.) and residuals (Obs.-Calc.) for the sevoflurane – 1 water complex (in MHz).

Table S13: Observed rotational transitions (Obs.) and residuals (Obs.-Calc.) for the C1 sevoflurane – 1 water complex (in MHz).

Table S14: Observed rotational transitions (Obs.) and residuals (Obs.-Calc.) for the C5 sevoflurane – 1 water complex (in MHz).

Table S15: Observed rotational transitions (Obs.) and residuals (Obs.-Calc.) for the C7 sevoflurane – 1 water complex (in MHz).

Table S16: Observed rotational transitions (Obs.) and residuals (Obs.-Calc.) for the O3 sevoflurane – 1 water complex (in MHz).

Table S17: Observed rotational transitions (Obs.) and residuals (Obs.-Calc.) for the O13 sevoflurane – 1 water complex (in MHz).

Table S18: Observed rotational transitions (Obs.) and residuals (Obs.-Calc.) for the sevoflurane – 2 water complex (in MHz).

Table S19: Observed rotational transitions (Obs.) and residuals (Obs.-Calc.) for the C1 sevoflurane – 2 water complex (in MHz).

Table S20: Observed rotational transitions (Obs.) and residuals (Obs.-Calc.) for the C2 sevoflurane – 2 water complex (in MHz).

Table S21: Observed rotational transitions (Obs.) and residuals (Obs.-Calc.) for the C3 sevoflurane – 2 water complex (in MHz).

Table S22: Observed rotational transitions (Obs.) and residuals (Obs.-Calc.) for the C4 sevoflurane – 2 water complex (in MHz).

Table S23: Observed rotational transitions (Obs.) and residuals (Obs.-Calc.) for the O1 sevoflurane – 2 water complex (in MHz).

Table S24: Observed rotational transitions (Obs.) and residuals (Obs.-Calc.) for the O2 sevoflurane – 2 water complex (in MHz).

Table S25: Observed rotational transitions (Obs.) and residuals (Obs.-Calc.) for the sevoflurane – 3 water complex (in MHz).

Table S26: Observed rotational transitions (Obs.) and residuals (Obs.-Calc.) for the C1 sevoflurane – 3 water complex (in MHz).

Table S27: Observed rotational transitions (Obs.) and residuals (Obs.-Calc.) for the C2 sevoflurane – 3 water complex (in MHz).

Table S28: Observed rotational transitions (Obs.) and residuals (Obs.-Calc.) for the C3 sevoflurane – 3 water complex (in MHz).

Table S29: Observed rotational transitions (Obs.) and residuals (Obs.-Calc.) for the C4 sevoflurane – 3 water complex (in MHz).

Table S30: Observed rotational transitions (Obs.) and residuals (Obs.-Calc.) for the O1 sevoflurane – 3 water complex (in MHz).

Table S31: Observed rotational transitions (Obs.) and residuals (Obs.-Calc.) for the O2 sevoflurane – 3 water complex (in MHz).

Table S32: Observed rotational transitions (Obs.) and residuals (Obs.-Calc.) for the O3 sevoflurane – 3 water complex (in MHz).

Table S33: Observed rotational transitions (Obs.) and residuals (Obs.-Calc.) for the sevoflurane – 4 water Clockwise complex (in MHz).

Table S34: Observed rotational transitions (Obs.) and residuals (Obs.-Calc.) for the sevoflurane – 4 water CounterClockwise complex (in MHz).

2.2 Least Squares Fit r_0 Structural Analysis

Table S35: The Sevofluarane-H_2O cluster.

Table S36: The Sevofluarane-$(H_2O)_2$ cluster.

Table S37: The Sevofluarane-$(H_2O)_3$ cluster.

3. NCI Plots

Figure S1. NCI plot for the SEV-(H_2O) cluster.

Figure S2. NCI plot for the SEV-$(H_2O)_2$ cluster.

Figure S3. NCI plot for the SEV-$(H_2O)_3$ cluster.

Figure S4. NCI plot for the SEV-$(H_2O)_4$ cluster.

Table S1. Calculated spectroscopic parameters of SEV-(H_2O) cluster

	B3LYP D3BJ aug-cc-pVTZ/ RI-MP2 cc-pVTZ								
	Rot. Consts (MHz)			Dipole (Debye)			(kJ mol^{-1})		
	A	B	C	μ_a	μ_b	μ_c	ΔE_e	ZPE	ΔG
1w-I*	823.0/830.8	642.9/658.8	528.1/538.7	2.6/2.7	1.1/1.0	0.9/1.2	0.0	0.0	0.0

*Only one isomer is reported. The next isomer is 10kJ/mol higher in energy

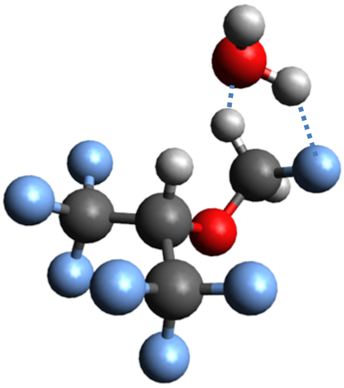

1w-I

Table S2. Calculated spectroscopic parameters of SEV-$(H_2O)_2$ cluster.

	B3LYP D3BJ aug-cc-pVTZ/ RI-MP2 cc-pVTZ								
	Rot. Consts (MHz)			Dipole (Debye)			(kJ mol^{-1})		
	A	B	C	μ_a	μ_b	μ_c	ΔE_e	ZPE	ΔG
2w-I*	747.3/756.3	476.9/490.9	424.7/434.3	1.6/1.5	1.0/0.9	0.7/0.7	0.0	0.0	0.0

*Only one isomer is reported. The next isomer is 5kJ/mol higher in energy.

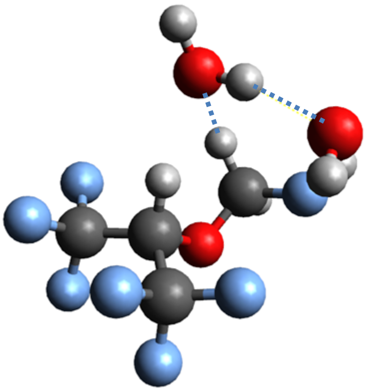

2w-I

Table S3. Calculated spectroscopic parameters of SEV-$(H_2O)_3$ cluster below 5kJ/mol.

	B3LYP D3BJ aug-cc-pVTZ/ RI-MP2 cc-pVTZ								
	Rot. Consts (MHz)			Dipole (Debye)			(kJ mol^{-1})		
	A	B	C	μ_a	μ_b	μ_c	ΔE_e	ZPE	ΔG
3w-I	616.7/604.0	369.0/390.7	332.2/346.7	1.8/1.8	1.4/0.9	0.5/0.7	0.0/2.1	0.0/0.5	0.0/0.0
3w-II	624.0/634.9	391.8/401.6	338.5/346.6	2.5/2.5	2.2/2.1	0.2/0.2	1.4/0.0	3.2/0.0	4.2/0.1
3w-III	627.6/640.7	384.8/396.7	332.6/340.6	2.7/2.8	2.3/2.1	0.1/0.0	2.0/0.5	4.0/0.6	4.8/1.0

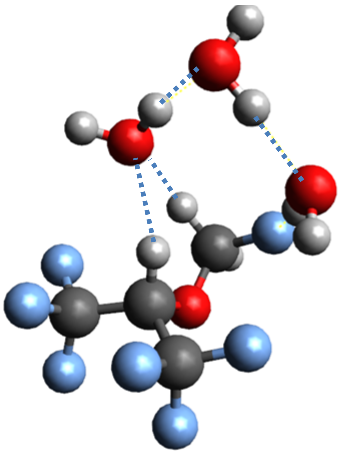

3w-I

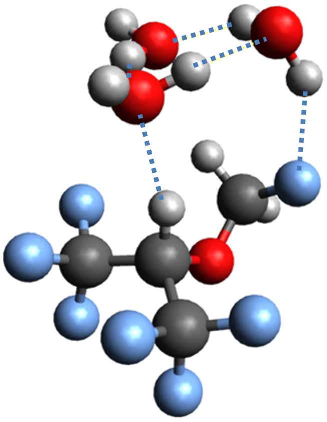

3w-II

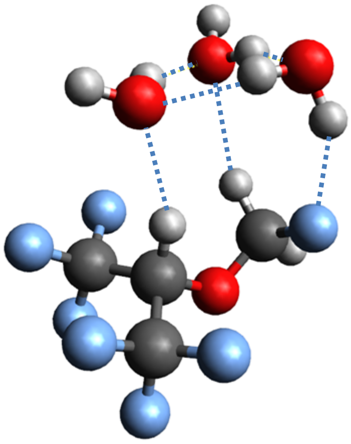

3w-III

Table S4. Calculated spectroscopic parameters of SEV-$(H_2O)_4$ cluster below 5kJ/mol.

	B3LYP D3BJ aug-cc-pVTZ/ RI-MP2 cc-pVTZ								
	Rot. Consts (MHz)			Dipole (Debye)			(kJ mol^{-1})		
	A	B	C	μ_a	μ_b	μ_c	ΔE_e	ZPE	ΔG
4w-I	502.7/503.8	365.5/380.3	320.7/333.5	1.7/1.7	0.9/0.8	0.8/0.8	0.0/0.0	0.0/0.0	0.0/0.0
4w-II	525.5/512.1	346.9/373.8	298.3/325.5	2.0/1.7	0.8/0.8	0.7/0.9	0.7/0.7	0.4/0.8	0.0/0.7
4w-III	496.3/486.9	353.0/376.2	346.1/365.9	0.7/0.7	2.7/2.2	0.1/1.3	2.8/3.1	1.7/1.7	0.4/0.0

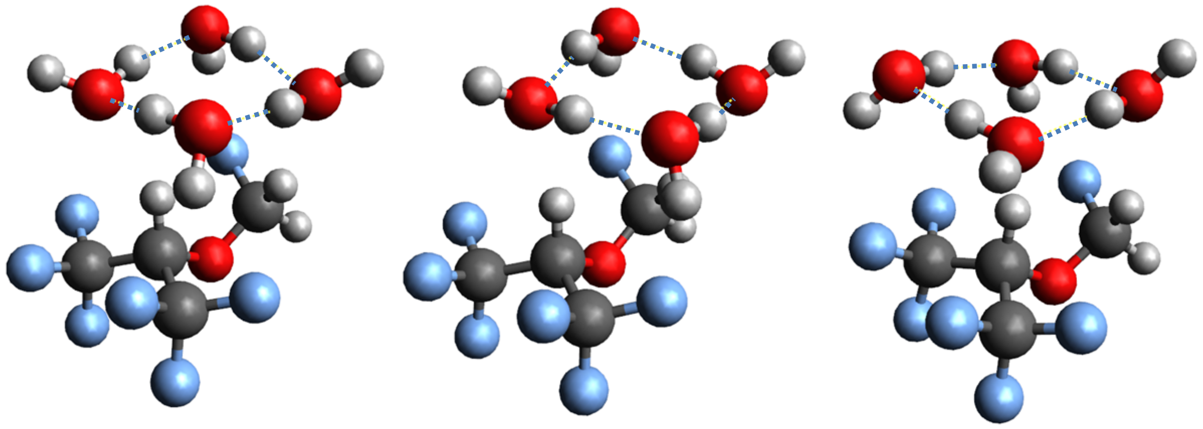

4w-I 4w-II 4w-III

Table S5. Spectroscopic parameters for SEV- (H_2O) cluster and its isotopologues.

	A/MHz	B/MHz	C/MHz	N [b]	σ/kHz [c]
Normal	822.23653(44)	645.85854(40)	533.76059(47)	193	4.8
C1	819.11534(70)	642.03232(64)	531.26993(38)	40	5.7
C5	820.95002(62)	644.58947(48)	532.35177(34)	52	3.2
C7	819.63397(66)	645.77566(50)	532.59909(37)	50	5.3
O3	817.88819(30)	642.76207(64)	533.19846(46)	24	5.8
O13	810.49226(39)	629.622105(31)	526.606887(29)	104	5.2

[a] The parent species centrifugal distortion values are: D_J = *0.0833(46) kHz*, D_{JK} = 0.573(11) *kHz*, D_K = -0.433(11) kHz, d_1 = -0.0133(23) *kHz* and d_2 = 9.70(70)Hz. For isotopically substituted species these parameters have been kept fixed at the values of the parent species. Uncertainties (in parentheses) are expressed in units of the last digit. [b] Number of transitions in the fit. [c] Standard deviation of the fit.

Atom mumbering is displayed in the structural analysis section.

Table S6. Experimental structures from the r_s method for the SEV-H_2O cluster.

	a/Å	*b/*Å	*c/*Å
C1	1.8303(9)	1.036(2)	1.143(1)
C5	1.239(1)	0.987(2)	0.031 *i [0]**
C7	0.325(5)	1.401(1)	0.080i
O3	0.611(2)	0.351(4)	1.235(1)
O13	2.4289(6)	0.639(2)	2.062(7)

*Imaginary coordinates are set to zero for structural considerations.

Table S7. Spectroscopic parameters for SEV-$(H_2O)_2$ cluster and its isotopologues.

	A/MHz [a]	*B*/MHz	*C*/MHz	*N* [b]	*σ*/kHz [c]
Normal	737.85016(16)	481.99047(75)	425.93344(55)	90	4.3
C1	737.1678(31)	480.67889(14)	424.74329(12)	39	12.4
C2	735.6918(30)	481.96070(17)	425.19668(14)	37	11.6
C3	737.840(35)	481.97180(23)	425.92665(27)	29	6.6
C4	733.552(35)	480.34767(20)	424.85318(21)	32	6.0
O1	735.88112(20)	469.05838(69)	416.34494(55)	82	7.9
O2	726.30016(28)	476.16626(73)	421.46746(58)	74	4.8

[a]The parent species centrifugal distortion values are: D_J = *0.1448(54) kHz,* D_{JK} = -0.373(27) *kHz*, D_K = 0.564(68) kHz, and d_1 = -0.0315(33) *kHz*. For isotopically substituted species these parameters have been kept fixed at the values of the parent species. Uncertainties (in parentheses) are expressed in units of the last digit. [c] Number of transitions in the fit. [d] Standard deviation of the fit.

Atom mumbering is displayed in the structural analysis section.

Table S8. Experimental structures from the r_s method for the SEV-$(H_2O)_2$ cluster.

	a/Å	*b*/Å	*c*/Å
C1	1.665(5)	0.74(1)	0.29(2)
C2	0.23(3)	1.415(5)	0.09(7)
C3	0.16(5)	0.078 *i [0]*	0.12(6)
C4	1.133(8)	1.304(7)	1.525(6)
O1	3.6926(4)	0.259(6)	0.952(2)
O2	1.8857(9)	1.618(1)	1.7187(9)

Table S9. Spectroscopic parameters for SEV-$(H_2O)_3$ cluster and its isotopologues.

	A/MHz [a]	*B*/MHz	*C*/MHz	*N* [b]	*σ*/kHz [c]
Normal	612.47396(75)	372.429002(30)	328.729677(30)	206	10.9
C1	612.5047(13)	372.34499(61)	328.67462(42)	31	3.3
C2	611.8305(14)	371.56946(81)	327.87766(42)	25	11.0
C3	610.9356(15)	372.23946(54)	328.21163(42)	30	5.2
C4	609.0368(16)	371.09775(73)	328.56690(47)	25	5.0
O1	608.32094(50)	362.644329(20)	321.237315(16)	99	7.2
O2	609.87781(78)	366.002307(26)	323.042320(19)	89	8.8
O3	605.02078(97)	370.843562(22)	325.612000(15)	89	7.6

[a] The parent species centrifugal distortion values are: D_J = *0.0949(13)kHz,* D_{JK} = -0.1687(39) *kHz*, D_K = 0.430(30) kHz, d_1 = -9.31(92) *kHz* and d_2 = -1.02(59) Hz. For isotopically substituted species these parameters have been kept fixed at the values of the parent species. [b] Number of transitions in the fit. [c] Standard deviation of the fit.

Atom mumbering is displayed in the structural analysis section.

Table S10. Experimental structures from the r_s method for the SEV-$(H_2O)_3$ cluster.

	a/Å	*b*/Å	*c*/Å
C1	0.550(8)	0.212 *i [0]*	0.06(7)
C2	1.769(3)	0.931(5)	0.08(6)
C3	0.719(7)	1.381(4)	0.42(1)
C4	0.695(8)	0.512(1)	2.096(3)
O1	4.0808(4)	1.131(1)	1.306(1)
O2	3.4402(5)	1.331(1)	0.269(6)
O3	1.598(1)	2.1973(7)	0.576(3)

Table S11. Spectroscopic parameters for SEV-$(H_2O)_4$ clusters.

	SEV-$(H_2O)_4$ -CW	SEV-$(H_2O)_4$ -CCW
A (MHz)	492.54315(10)	504.68006(14)
B (MHz)	368.83715(10)	359.21357(12)
C (MHz)	328.372229(94)	316.06881(10)
Δ_J (kHz)	0.09307(46)	0.12512(50)
Δ_{JK} (kHz)	-0.1478(20)	-0.1768(21)
Δ_K (kHz)	0.1705(21)	0.1955(26)
δ_J (kHz)	-0.00372(27)	-0.00687(29)
δ_K (kHz)	0.0607(43)	0.1109(44)
σ	10.9	4.2
N	199	180

A, B and C are the rotational constants. Δ_J , Δ_{JK}, Δ_K, δ_J , δ_K are the centrifugal distortion constants in the Watson's A-reduction. σ is the rms deviation of the fit, and N is the number of transitions in the fit.

Table S12. Observed rotational transitions (Obs.) and residuals (Obs.-Calc.) for the sevoflurane – 1 water complex (in MHz).

J'	Ka'	Kc'	←	J''	Ka''	Kc''	υ_{obs}	υ_{obs}-υ_{cal}
2	0	2	←	1	0	1	2320.3173	0.0006
2	1	1	←	1	1	0	2471.3338	0.0029
3	1	3	←	2	1	2	3349.446	0.0016
3	0	3	←	2	0	2	3406.5574	0.0019
3	2	2	←	2	2	1	3538.8337	0
3	2	1	←	2	2	0	3671.1266	-0.0017
3	1	2	←	2	1	1	3676.6553	-0.0009
4	1	4	←	3	1	3	4436.3211	0.0012
4	2	3	←	3	2	2	4687.2456	-0.0004
4	3	2	←	3	3	1	4776.351	-0.0011
4	3	1	←	3	3	0	4825.8389	-0.002
4	1	3	←	3	1	2	4835.2153	-0.0005
4	2	2	←	3	2	1	4938.5829	-0.0018
5	1	5	←	4	1	4	5512.6689	0.0003
5	0	5	←	4	0	4	5524.0717	-0.0003
5	2	4	←	4	2	3	5812.4562	-0.0015
5	1	4	←	4	1	3	5932.1775	-0.0006
5	3	3	←	4	3	2	5964.6826	-0.0005
5	3	2	←	4	3	1	6105.3477	-0.0019
5	2	3	←	4	2	2	6173.3376	-0.0016
6	1	6	←	5	1	5	6583.5614	0.001
6	0	6	←	5	0	5	6587.4477	-0.0005
6	2	5	←	5	2	4	6915.4525	-0.0006
6	1	5	←	5	1	4	6986.5428	0.0009
6	3	4	←	5	3	3	7133.5562	0.001
6	2	4	←	5	2	3	7353.2294	0.0009
6	3	3	←	5	3	2	7400.2665	-0.0006
7	1	7	←	6	1	6	7652.1648	-0.0018
7	0	7	←	6	0	6	7653.3869	-0.0019
4	0	4	←	3	0	3	4465.3831	0.0015
2	1	2	←	1	1	1	2247.1375	0.0017
5	4	2	←	4	4	1	5983.5294	-0.0014
5	4	1	←	4	4	0	5996.6381	-0.0023
6	5	2	←	5	5	1	7179.291	0.0013
6	5	1	←	5	5	0	7182.1616	0.0006
6	4	3	←	5	4	2	7194.4231	0.001
6	4	2	←	5	4	1	7246.8083	-0.0007
2	0	2	←	1	1	1	2143.9418	0.0024
2	1	2	←	1	0	1	2423.5153	0.0022
2	2	1	←	1	1	0	3000.4643	0.003
3	0	3	←	2	1	2	3303.3595	0.0004

3	1	3	←	2	0	2	3452.6431	0.0023
3	2	2	←	2	1	1	4067.9666	0.0025
4	0	4	←	3	1	3	4419.2975	0.0012
4	1	3	←	3	2	2	4443.9065	-0.0016
4	1	4	←	3	0	3	4482.4067	0.0017
3	3	1	←	2	2	0	4683.2978	-0.0013
3	3	0	←	2	2	1	4731.2956	-0.002
4	2	3	←	3	1	2	5078.5545	0.0006
5	0	5	←	4	1	4	5507.0508	0.0023
5	1	5	←	4	0	4	5529.692	-0.0001
5	1	4	←	4	2	3	5688.8383	-0.0019
4	3	2	←	3	2	1	5788.5223	-0.0006
5	2	4	←	4	1	3	6055.7956	0
5	2	3	←	4	3	2	5323.397	-0.004
4	4	1	←	3	3	0	6346.2508	-0.0017
4	4	0	←	3	3	1	6357.0694	-0.0017
6	0	6	←	5	1	5	6581.8269	-0.0012
6	1	6	←	5	0	5	6589.18	-0.0003
6	2	4	←	5	3	3	6711.9449	-0.0015
5	3	3	←	4	2	2	6814.6214	0.0002
6	1	5	←	5	2	4	6862.9246	0.0003
6	2	5	←	5	1	4	7039.0715	0.0008
4	3	2	←	3	0	3	7060.5983	-0.0366
5	3	2	←	4	2	3	7436.4063	-0.0021
5	4	2	←	4	3	1	7503.9414	-0.0009
5	4	1	←	4	3	2	7577.3582	-0.0012
7	0	7	←	6	1	6	7651.6547	-0.0021
7	1	7	←	6	0	6	7653.8972	-0.0016
6	3	4	←	5	2	3	7774.8398	0.0026
7	1	6	←	6	2	5	7981.7062	-0.0007
5	5	1	←	4	4	0	7995.3415	-0.0014
5	5	0	←	4	4	1	7997.382	-0.0008
2	1	1	←	1	0	1	2759.8036	-0.0023
2	2	0	←	1	1	0	3039.3833	0.0027
2	2	1	←	1	1	1	3112.5635	0.0044
3	2	1	←	2	1	1	4239.1766	-0.0015
4	1	3	←	3	2	1	4272.6923	-0.0017
3	3	0	←	2	2	0	4692.3773	-0.0008
3	3	1	←	2	2	1	4722.2172	-0.0013
5	2	3	←	4	3	1	5264.8309	-0.0023
5	1	4	←	4	2	2	5266.2872	-0.0001
4	2	2	←	3	1	2	5501.1064	-0.0003
4	2	3	←	3	1	3	5742.0624	0.0036
4	3	1	←	3	2	1	5847.0906	-0.0001
4	3	2	←	3	2	2	5959.7363	-0.0007
4	4	0	←	3	3	0	6347.9912	-0.0009
4	4	1	←	3	3	1	6355.3297	-0.0018

6	2	4	←	5	3	2	6512.7119	-0.0001
5	2	3	←	4	1	3	6839.2278	-0.0022
5	1	4	←	4	0	4	7011.6042	0.0014
5	3	2	←	4	2	2	7013.854	-0.0015
5	2	4	←	4	1	4	7118.1981	0.0014
5	3	3	←	4	2	3	7237.1725	-0.0015
7	3	4	←	6	4	2	7405.1262	-0.001
5	4	1	←	4	3	1	7518.792	0.0003
5	4	2	←	4	3	2	7562.5095	-0.0007
7	2	5	←	6	3	3	7576.8946	0
5	5	0	←	4	4	0	7995.6438	0.0006
5	5	1	←	4	4	1	7997.0813	-0.0012
7	5	2	←	7	4	4	2016.2373	-0.0033
6	4	3	←	6	2	4	2080.3011	-0.0016
8	5	3	←	8	4	5	2103.3136	-0.0034
9	5	5	←	9	4	6	2125.6872	0.0022
8	2	6	←	8	2	7	2126.2018	-0.0019
8	2	6	←	8	1	7	2133.0119	0.0022
9	6	3	←	9	5	4	2207.0433	-0.0028
9	4	6	←	9	3	7	2219.5461	0.0023
8	3	6	←	8	2	7	2236.412	0.001
5	4	2	←	5	2	3	2239.1128	0.0037
8	3	6	←	8	1	7	2243.2184	0.0015
7	1	6	←	7	1	7	2266.9585	-0.0028
7	1	6	←	7	0	7	2267.4721	0.0008
7	2	6	←	7	1	7	2286.6622	-0.0008
7	2	6	←	7	0	7	2287.1695	-0.0034
8	6	3	←	8	5	3	2339.3562	-0.0019
8	6	2	←	8	5	3	2343.4138	0.0012
9	6	4	←	9	5	5	2391.3548	0.0014
8	6	2	←	8	5	4	2412.4869	0.0011
7	6	2	←	7	5	2	2418.3298	0.0037
7	6	1	←	7	5	2	2418.9395	0.003
7	6	2	←	7	5	3	2436.112	0.0001
7	6	1	←	7	5	3	2436.7263	0.004
6	6	0	←	6	5	1	2458.5895	0.0046
6	6	1	←	6	5	2	2461.7021	-0.0056
9	2	7	←	9	1	8	2536.4921	0.002
9	3	7	←	9	2	8	2579.7616	0.001
8	1	7	←	8	1	8	2636.8332	-0.0037
8	1	7	←	8	0	8	2636.9815	-0.0007
8	2	7	←	8	1	8	2643.6418	-0.0011
8	2	7	←	8	0	8	2643.788	-0.0001
9	7	2	←	9	6	3	2833.6818	-0.0014
9	7	3	←	9	6	4	2851.5891	0.0004
8	7	2	←	8	6	2	2882.5195	-0.0025
8	7	1	←	8	6	2	2882.6298	-0.0017

8	7	2	←	8	6	3	2886.5735	-0.003
8	7	1	←	8	6	3	2886.6866	0.0006
7	7	1	←	7	6	1	2912.5905	0.012
3	1	2	←	2	2	0	3108.6016	-0.0048
3	1	2	←	2	2	1	3147.5221	-0.0037
2	2	0	←	1	0	1	3327.8573	0.0015
6	4	2	←	6	1	5	3421.2775	0
4	2	3	←	3	3	0	3494.7973	0.015
4	2	2	←	3	3	0	3917.331	-0.004
4	2	2	←	3	3	1	3926.4108	-0.0032
3	1	2	←	2	0	2	4116.1501	0.0044
5	1	4	←	4	3	1	4357.7758	-0.0056
5	3	3	←	4	4	0	4383.963	-0.001
3	2	2	←	2	1	2	4404.2687	0.0118
5	2	4	←	4	3	1	4481.3924	-0.0065
5	2	4	←	4	3	2	4539.9627	-0.004
3	2	1	←	2	1	2	4575.4891	0.018
3	2	1	←	2	0	2	4678.6807	0.0132
6	2	4	←	5	4	1	5099.2648	-0.0053
6	0	6	←	5	1	4	5099.9092	-0.0082
6	4	3	←	5	5	0	5180.5691	-0.001
6	4	3	←	5	5	1	5180.8851	0.0147
6	1	5	←	5	3	2	5238.9736	0
6	4	2	←	5	5	0	5247.8075	0.0011
6	4	2	←	5	5	1	5248.1069	0.0002
3	3	0	←	2	1	1	5260.4275	-0.0004
6	2	5	←	5	3	2	5291.5057	0.0032
6	2	5	←	5	3	3	5490.7345	-0.0023
6	3	4	←	5	4	1	5520.8748	-0.0039
6	3	4	←	5	4	2	5535.7286	0.0006
4	1	3	←	3	0	3	5544.8085	0.0024
7	0	7	←	6	1	5	5766.7653	0.0007
6	3	3	←	5	4	1	5986.8238	-0.0012
6	3	3	←	5	4	2	6001.6723	-0.002
4	3	1	←	3	2	2	6018.2951	-0.0097
5	2	4	←	4	1	3	6055.7972	0.0015
6	1	5	←	5	2	3	6079.4918	0.0019
4	2	2	←	3	1	3	6164.6276	0.016
7	2	5	←	6	4	2	6316.9102	-0.0004
7	2	6	←	6	3	4	6358.5896	-0.0007
7	4	4	←	6	5	1	6396.0837	0.0006
7	4	4	←	6	5	2	6399.2666	0.012
4	3	1	←	3	1	2	6409.6109	-0.0017
7	4	3	←	6	5	1	6603.5706	-0.0026
7	4	3	←	6	5	2	6606.7484	0.0035
7	3	5	←	6	4	3	6618.4665	0.0074
7	1	6	←	6	2	4	6760.5	0.0026

4	3	2	←	3	1	3	7014.5673	0.0176
8	5	4	←	7	6	1	7182.0467	-0.0049
8	5	3	←	7	6	2	7251.7389	0.0036
8	2	6	←	7	4	3	7297.8709	-0.0006
4	4	0	←	3	2	1	7369.2394	-0.0025
8	3	6	←	7	4	3	7408.0822	0.0034
7	3	4	←	6	4	3	7472.3615	-0.0019
4	4	1	←	3	2	2	7538.7199	0.0034
8	4	5	←	7	5	2	7566.7454	0.0011
8	4	5	←	7	5	3	7584.5276	-0.0024
8	3	6	←	7	4	4	7615.5687	-0.0001

Table S13 Observed rotational transitions (Obs.) and residuals (Obs.-Calc.) for the C1 sevoflurane – 1 water complex (in MHz).

J'	Ka'	Kc'	←	J''	Ka''	Kc''	υ_{obs}	υ_{obs}-υ_{cal}
4	1	4	←	3	1	3	4414.88652	-0.00157
4	0	4	←	3	0	3	4444.41082	0.03043
4	2	3	←	3	2	2	4662.71378	-0.00118
4	2	2	←	3	2	1	4909.8703	0.00009
5	1	5	←	4	1	4	5486.3508	-0.00376
5	0	5	←	4	0	4	5498.05754	-0.00193
5	2	4	←	4	2	3	5782.81185	-0.00463
5	1	4	←	4	1	3	5903.16907	-0.00219
5	3	2	←	4	3	1	6068.79397	0.00082
5	2	3	←	4	2	2	6138.72695	-0.00288
6	1	6	←	5	1	5	6552.33929	-0.0009
6	0	6	←	5	0	5	6556.3713	-0.0027
6	2	5	←	5	2	4	6881.0243	-0.00055
6	1	5	←	5	1	4	6953.41234	0.0009
6	3	4	←	5	3	3	7095.40548	0.00128
6	2	4	←	5	2	3	7314.21789	-0.00312
6	3	3	←	5	3	2	7356.18236	0.00187
7	1	7	←	6	1	6	7615.9981	-0.00471
7	0	7	←	6	0	6	7617.28632	0.00208
7	2	6	←	6	2	5	7962.28918	-0.00035
3	1	2	←	2	1	1	3656.48881	0.00133
3	0	3	←	2	0	2	3390.25111	-0.00168
3	1	3	←	2	1	2	3332.96097	-0.00191
6	4	3	←	5	4	2	7154.14098	-0.00258
6	4	2	←	5	4	1	7204.28644	0.0007
4	1	4	←	3	0	3	4461.93889	-0.00387
3	3	1	←	2	2	0	4664.9591	-0.00288
5	0	5	←	4	1	4	5480.4968	-0.0003
4	3	2	←	3	2	1	5765.35773	0.00443
4	4	0	←	3	3	1	6331.71112	0.00161
6	0	6	←	5	1	5	6550.51126	-0.00528
6	1	5	←	5	2	4	6826.49014	0.0077
5	4	2	←	4	3	1	7473.51738	-0.0037
5	4	1	←	4	3	2	7544.29721	-0.00113
7	0	7	←	6	1	6	7615.46216	0.00158
7	1	7	←	6	0	6	7617.82768	0.00121
5	5	0	←	4	4	1	7965.92875	0.00247
5	4	1	←	4	3	1	7487.68067	-0.00347
5	4	2	←	4	3	2	7530.13896	0.00366
5	5	1	←	4	4	1	7965.64351	-0.00034

Table S14 Observed rotational transitions (Obs.) and residuals (Obs.-Calc.) for the C5 sevoflurane – 1 water complex (in MHz).

J’	Ka’	Kc’	←	J’’	Ka’’	Kc’’	υ_{obs}	υ_{obs}-υ_{cal}
4	1	4	←	3	1	3	4425.21744	-0.00043
4	0	4	←	3	0	3	4454.24906	-0.00129
4	2	3	←	3	2	2	4676.46237	-0.00291
4	3	2	←	3	3	1	4765.75096	-0.00256
4	3	1	←	3	3	0	4815.39327	0.0002
4	1	3	←	3	1	2	4824.51912	-0.0014
4	2	2	←	3	2	1	4928.21462	-0.00177
5	1	5	←	4	1	4	5498.73792	-0.00433
5	0	5	←	4	0	4	5510.11774	-0.00565
5	2	4	←	4	2	3	5798.88002	-0.00348
5	1	4	←	4	1	3	5918.57713	-0.00061
5	3	3	←	4	3	2	5951.39594	0.00112
5	4	2	←	4	4	1	5970.32879	0.00109
5	3	2	←	4	3	1	6092.43264	0.00119
5	2	3	←	4	2	2	6160.26959	0.00007
6	1	6	←	5	1	5	6566.80945	-0.00004
6	0	6	←	5	0	5	6570.68527	-0.00097
6	2	5	←	5	2	4	6899.05199	-0.00068
6	1	5	←	5	1	4	6970.04978	0.00074
6	3	4	←	5	3	3	7117.52375	0.00185
6	5	1	←	5	5	0	7166.33989	-0.00092
6	4	3	←	5	4	2	7178.5883	0.00325
6	4	2	←	5	4	1	7231.17905	0.00211
6	2	4	←	5	2	3	7337.31699	-0.00017
4	4	0	←	3	2	1	7358.89778	0.00206
6	3	3	←	5	3	2	7384.79981	0.0006
7	1	7	←	6	1	6	7632.59423	0.0013
7	0	7	←	6	0	6	7633.81011	-0.00054
7	2	6	←	6	2	5	7982.1693	0.00026
3	1	2	←	2	1	1	3668.76026	0.00241
3	0	3	←	2	0	2	3398.26475	-0.00251
3	1	3	←	2	1	2	3341.15595	-0.00206
5	0	5	←	4	1	4	5493.13803	-0.00238
5	1	5	←	4	0	4	5515.72188	-0.00336
5	2	4	←	4	1	3	6041.94806	-0.00092
4	4	1	←	3	3	0	6335.90444	0.00059
4	4	0	←	3	3	1	6346.76589	0.00637
6	0	6	←	5	1	5	6565.09444	0.01004
6	1	6	←	5	0	5	6572.41176	0.00043
6	2	4	←	5	3	3	6696.51503	-0.0035
6	1	5	←	5	2	4	6846.67685	-0.00095

5	4	2	←	4	3	1	7490.83843	-0.00005
5	4	1	←	4	3	2	7564.49666	-0.00163
7	0	7	←	6	1	6	7632.08698	0.00142
7	1	7	←	6	0	6	7634.32113	0.00311
7	1	6	←	6	2	5	7962.54831	0.00519
4	3	1	←	3	2	1	5836.62694	-0.01086
4	3	2	←	3	2	2	5949.44641	0.00286
4	4	1	←	3	3	1	6345.0102	-0.00219
5	3	3	←	4	2	3	7224.38066	0.00757
5	4	1	←	4	3	1	7505.75109	0.0009
5	4	2	←	4	3	2	7549.57827	-0.0083

Table S15 Observed rotational transitions (Obs.) and residuals (Obs.-Calc.) for the C7 sevoflurane – 1 water complex (in MHz).

J’	Ka’	Kc’	←	J’’	Ka’’	Kc’’	υ_{obs}	υ_{obs}-υ_{cal}
4	0	4	←	3	0	3	4456.08254	-0.00073
4	2	3	←	3	2	2	4681.40492	-0.00101
4	3	2	←	3	3	1	4772.65178	-0.00493
4	1	3	←	3	1	2	4829.03556	0.02333
4	2	2	←	3	2	1	4936.94594	-0.00403
5	1	5	←	4	1	4	5501.70064	-0.006
5	0	5	←	4	0	4	5512.58606	0.01021
5	2	4	←	4	2	3	5803.99092	0.00085
5	1	4	←	4	1	3	5921.70414	-0.00245
5	3	3	←	4	3	2	5959.40998	-0.00074
5	3	2	←	4	3	1	6104.9513	-0.00191
5	2	3	←	4	2	2	6169.30751	-0.00086
4	3	1	←	3	1	2	6390.8426	0.00031
6	1	6	←	5	1	5	6570.13569	0.00249
6	0	6	←	5	0	5	6573.77862	-0.00077
6	2	5	←	5	2	4	6904.0626	0.00002
6	1	5	←	5	1	4	6972.6205	0.00121
6	3	4	←	5	3	3	7125.96026	0.00085
6	5	2	←	5	5	1	7174.68405	0.00486
6	4	3	←	5	4	2	7189.58395	-0.00029
6	4	2	←	5	4	1	7245.0349	0.00283
6	2	4	←	5	2	3	7344.97731	0.00324
6	3	3	←	5	3	2	7399.39415	-0.00141
7	1	7	←	6	1	6	7636.34824	0.00084
7	0	7	←	6	0	6	7637.47834	0.00248
7	2	6	←	6	2	5	7987.10215	0.00021
3	1	3	←	2	1	2	3343.56845	0.00355
3	0	3	←	2	0	2	3399.91896	0.00232
3	2	2	←	2	2	1	3535.09665	-0.00436
3	1	2	←	2	1	1	3673.58892	-0.00038
4	3	1	←	3	3	0	4824.24342	-0.00379
4	0	4	←	3	1	3	4411.79605	-0.00406
4	1	4	←	3	0	3	4472.18741	-0.00049
3	3	1	←	2	2	0	4669.17115	0.01441
4	3	2	←	3	2	1	5771.51485	0.00319
4	3	1	←	3	2	2	6007.75838	-0.00094
5	2	4	←	4	1	3	6039.46207	-0.00468
4	4	1	←	3	3	0	6327.32608	0.00186
4	4	0	←	3	3	1	6338.6768	-0.00351
6	0	6	←	5	1	5	6568.53676	-0.00721
6	1	6	←	5	0	5	6575.36529	-0.00334

6	2	4	←	5	3	3	6720.31003	0
6	2	5	←	5	1	4	7021.81707	-0.00567
5	4	2	←	4	3	1	7482.6311	-0.00199
5	4	1	←	4	3	2	7559.52577	-0.00122
7	1	6	←	6	2	5	7968.9482	0.00164
4	4	1	←	3	3	1	6336.81825	-0.00679
5	3	3	←	4	2	3	7224.66541	-0.00736
5	4	1	←	4	3	1	7498.43537	-0.0003
5	4	2	←	4	3	2	7543.72939	0.00497

Table S16 Observed rotational transitions (Obs.) and residuals (Obs.-Calc.) for the O3 sevoflurane – 1 water complex (in MHz).

J’	Ka’	Kc’	←	J’’	Ka’’	Kc’’	υ_{obs}	υ_{obs}-υ_{cal}
7	0	7	←	6	0	6	7642.32711	0.00331
7	1	7	←	6	1	6	7641.06224	0.00501
7	2	6	←	6	2	5	7983.58485	0.00248
6	3	3	←	5	3	2	7368.74381	0.00404
6	2	4	←	5	2	3	7327.17263	-0.00188
6	1	5	←	5	1	4	6970.2422	-0.00428
6	2	5	←	5	2	4	6898.67497	0.00368
6	0	6	←	5	0	5	6577.53705	-0.00208
6	1	6	←	5	1	5	6573.55166	0.00012
5	2	3	←	4	2	2	6149.06936	-0.00493
5	3	3	←	4	3	2	5944.6775	-0.00229
5	1	4	←	4	1	3	5916.03581	0.00879
5	2	4	←	4	2	3	5796.99796	-0.003
5	0	5	←	4	0	4	5515.32012	-0.00103
5	1	5	←	4	1	4	5503.73653	-0.01217
5	1	5	←	4	0	4	5521.1136	0.0032
5	4	2	←	4	4	1	5962.3913	-0.0122
5	4	1	←	4	4	0	5974.7842	0.0034
4	3	1	←	3	2	2	5986.0652	-0.0051
5	3	2	←	4	3	1	6079.9206	-0.0101
6	1	6	←	5	0	5	6579.3442	0.0009
6	3	4	←	5	3	3	7110.748	0.0035
6	5	2	←	5	5	1	7153.8531	-0.0021
6	4	2	←	5	4	1	7218.4969	0.0066

Table S17 Observed rotational transitions (Obs.) and residuals (Obs.-Calc.) for the O13 sevoflurane – 1 water complex (in MHz).

J’	Ka’	Kc’	←	J’’	Ka’’	Kc’’	υ_{obs}	υ_{obs}-υ_{cal}
7	1	7	←	6	1	6	7543.0223	0.0007
7	0	7	←	6	0	6	7544.6953	-0.002
7	2	6	←	6	2	5	7871.588	-0.0149
7	1	6	←	6	1	5	7911.7921	-0.007
6	2	4	←	5	2	3	7203.4334	0.0065
6	3	4	←	5	3	3	6991.0483	-0.001
6	1	5	←	5	1	4	6877.7176	0.0003
6	2	5	←	5	2	4	6798.0452	0.0022
6	0	6	←	5	0	5	6493.3994	-0.0017
6	1	6	←	5	1	5	6488.4321	-0.0023
5	2	3	←	4	2	2	6035.0607	0.0005
5	3	3	←	4	3	2	5840.8552	-0.0054
5	2	4	←	4	2	3	5708.2451	0.0017
5	0	5	←	4	0	4	5444.8793	0.0175
5	1	5	←	4	1	4	5431.317	-0.0044
4	1	3	←	3	1	2	4741.5833	0.0008
4	2	3	←	3	2	2	4598.4864	0.0019
4	0	4	←	3	0	3	4400.5799	0.0074
4	1	4	←	3	1	3	4368.6362	0
3	1	2	←	2	1	1	3597.9169	-0.0155
3	2	2	←	2	2	1	3468.6702	0.0069
3	0	3	←	2	0	2	3353.9905	0.003
2	1	2	←	1	1	1	2209.4399	0.0017
2	0	2	←	1	0	1	2279.3833	0.0019
2	1	1	←	1	1	0	2415.4706	0.0028
3	1	3	←	2	1	2	3295.9532	0.0023
3	2	1	←	2	2	0	3583.3539	-0.0016
4	3	2	←	3	3	1	4675.0775	-0.0024
4	3	1	←	3	3	0	4714.1829	-0.0049
4	2	2	←	3	2	1	4821.5709	-0.001
5	1	4	←	4	1	3	5831.3889	0.0019
5	4	2	←	4	4	1	5853.8377	-0.0061
5	4	1	←	4	4	0	5863.2672	-0.0106
5	3	2	←	4	3	1	5955.6604	-0.0012
4	3	1	←	3	1	2	6314.673	0.0004
6	5	2	←	5	5	1	7023.131	-0.016
6	5	1	←	5	5	0	7025.0169	-0.0189
6	4	3	←	5	4	2	7038.2619	-0.0051
6	4	2	←	5	4	1	7076.7653	-0.0059
6	3	3	←	5	3	2	7217.9479	0.002
5	3	2	←	4	1	3	7528.7481	-0.0037

2	1	2	←	1	0	1	2390.3137	0.006
7	7	1	←	7	6	2	2929.5695	0.0054
2	2	1	←	1	1	0	2958.0799	0.0052
3	1	2	←	2	2	1	3055.3253	-0.0003
2	2	0	←	1	1	1	3094.1713	0.0071
3	0	3	←	2	1	2	3243.0623	0.0011
3	1	3	←	2	0	2	3406.8783	0.0011
3	2	2	←	2	1	1	4011.2725	0.0023
4	1	3	←	3	2	2	4328.2473	0.0024
4	0	4	←	3	1	3	4347.6844	0.0016
4	1	4	←	3	0	3	4421.5291	0.003
3	3	1	←	2	2	0	4615.6742	-0.0006
3	3	0	←	2	2	1	4655.8126	0.0022
4	2	3	←	3	1	2	5011.8244	0.0023
5	2	3	←	4	3	2	5149.2359	0.003
5	0	5	←	4	1	4	5423.9113	0.0031
5	1	5	←	4	0	4	5452.2778	0.0027
5	1	4	←	4	2	3	5561.1514	0.004
4	3	2	←	3	2	1	5707.3994	0.0001
4	3	1	←	3	2	2	5901.3355	0.0005
5	2	4	←	4	1	3	5978.4846	0.0016
4	4	1	←	3	3	0	6252.6186	-0.0007
4	4	0	←	3	3	1	6260.9251	0.0052
6	0	6	←	5	1	5	6485.9895	0.0017
6	1	6	←	5	0	5	6495.8508	0.0031
6	2	4	←	5	3	3	6511.8127	0.0136
5	3	3	←	4	2	2	6726.688	0
6	1	5	←	5	2	4	6730.6266	0.0053
6	2	5	←	5	1	4	6945.1394	0.0005
5	3	2	←	4	2	3	7258.5078	-0.0044
5	4	2	←	4	3	1	7392.2732	-0.0021
5	4	1	←	4	3	2	7449.1163	-0.0014
7	0	7	←	6	1	6	7542.25	-0.0008
6	3	4	←	5	2	3	7682.6741	-0.003
7	2	5	←	6	3	4	7833.6177	0.0087
7	1	6	←	6	2	5	7844.3789	0.0013
5	5	1	←	4	4	0	7877.2213	-0.0023
5	5	0	←	4	4	1	7878.661	0.0019
7	2	6	←	6	1	5	7939.0258	0.0012
2	1	1	←	1	0	1	2699.3527	0.0003
2	2	0	←	1	1	0	2991.1521	0.003
2	2	1	←	1	1	1	3061.0945	0.0047
3	1	2	←	2	0	2	4017.9044	0.0009
3	2	1	←	2	1	1	4159.0373	0.0005
3	3	0	←	2	2	0	4622.7374	0.0013
3	3	1	←	2	2	1	4648.7482	-0.0011
4	2	2	←	3	1	2	5382.6758	-0.0004

4	1	3	←	3	0	3	5405.5054	0.0069
4	2	3	←	3	1	3	5622.8524	0.0039
4	3	1	←	3	2	1	5753.5676	-0.0007
4	3	2	←	3	2	2	5855.1659	0
4	4	0	←	3	3	0	6253.8624	0.0036
4	4	1	←	3	3	1	6259.6792	-0.0012
6	2	4	←	5	3	2	6350.8379	0.0089
5	2	3	←	4	1	3	6676.1524	-0.0014
5	1	4	←	4	0	4	6836.3173	0.0041
5	3	2	←	4	2	2	6887.6552	-0.0029
5	2	4	←	4	1	4	6962.454	-0.0014
5	3	3	←	4	2	3	7097.5403	-0.0018
5	4	1	←	4	3	1	7402.9485	-0.0001
5	4	2	←	4	3	2	7438.4418	-0.0026
5	5	0	←	4	4	0	7877.4218	0.0021
5	5	1	←	4	4	1	7878.4623	-0.0006

Table S18 Observed rotational transitions (Obs.) and residuals (Obs.-Calc.) for the sevoflurane – 2 water complex (in MHz).

J'	Ka'	Kc'	←	J''	Ka''	Kc''	υ_{obs}	υ_{obs}-υ_{cal}
3	1	3	←	2	1	2	2634.85383	-0.00121
3	0	3	←	2	0	2	2691.90493	-0.00178
3	2	2	←	2	2	1	2723.76054	-0.00453
3	2	1	←	2	2	0	2755.61728	0.00282
3	1	2	←	2	1	1	2802.25397	0.00193
4	1	4	←	3	1	3	3505.12518	-0.00027
4	0	4	←	3	0	3	3558.12927	-0.00179
4	2	3	←	3	2	2	3625.22841	0.00217
4	1	3	←	3	1	2	3724.80382	0.0028
5	1	5	←	4	1	4	4370.42469	-0.00013
5	0	5	←	4	0	4	4410.65219	-0.00149
5	2	4	←	4	2	3	4521.28831	0.00463
5	4	2	←	4	4	1	4557.8897	0.00432
5	4	1	←	4	4	0	4558.35544	0.00155
5	3	3	←	4	3	2	4560.78509	0.00508
5	3	2	←	4	3	1	4576.1594	-0.00035
5	1	4	←	4	1	3	4635.29768	0.00305
5	2	3	←	4	2	2	4649.70351	-0.00514
6	1	6	←	5	1	5	5231.30859	0.00021
6	0	6	←	5	0	5	5257.4157	-0.00136
6	2	5	←	5	2	4	5410.90987	0.00249
6	3	4	←	5	3	3	5474.63628	-0.001
6	4	3	←	5	4	2	5474.86372	-0.00175
6	4	2	←	5	4	1	5476.93695	-0.00084
6	3	3	←	5	3	2	5513.22802	-0.00488
6	1	5	←	5	1	4	5528.96377	0.00408
7	1	7	←	6	1	6	6088.75628	0.00245
7	0	7	←	6	0	6	6103.93933	-0.00079
7	2	6	←	6	2	5	6293.47041	0.00144
7	3	5	←	6	3	4	6385.69116	-0.00302
7	4	4	←	6	4	3	6393.86762	-0.00378
7	4	3	←	6	4	2	6400.57956	-0.00207
7	1	6	←	6	1	5	6402.60527	0.00443
8	1	8	←	7	1	7	6943.81323	0.00286
8	0	8	←	7	0	7	6952.02457	0.00189
8	2	7	←	7	2	6	7168.89616	0.00146
8	1	7	←	7	1	6	7257.8421	0.00052
9	1	9	←	8	1	8	7797.35798	-0.00025
9	0	9	←	8	0	8	7801.58611	-0.00035
7	0	7	←	6	1	6	6072.28859	0.0009

7	1	7	←	6	0	6	6120.40359	-0.00267
8	0	8	←	7	1	7	6935.55832	0.00178
8	1	8	←	7	0	7	6960.27675	0.00024
9	0	9	←	8	1	8	7793.33449	0.00186
9	1	9	←	8	0	8	7805.61082	-0.00124
4	1	4	←	3	0	3	3656.12115	0.00012
5	0	5	←	4	1	4	4312.66763	0.00392
4	3	2	←	3	2	1	5030.27634	0.00397
6	1	5	←	5	2	4	5053.44797	-0.00597
4	3	1	←	3	2	2	5075.66667	-0.00059
5	2	4	←	4	1	3	5110.7944	-0.00598
6	0	6	←	5	1	5	5199.65883	0.00287
6	1	6	←	5	0	5	5289.06799	-0.0015
4	4	0	←	3	3	1	5620.53716	-0.00166
4	4	1	←	3	3	0	5619.70842	-0.00401
6	2	5	←	5	1	4	5886.4043	-0.00883
5	3	3	←	4	2	2	5892.36152	0.00379
5	3	2	←	4	2	3	6026.59316	-0.00762
7	1	6	←	6	2	5	6045.14453	-0.00287
8	2	6	←	7	3	5	6496.28089	0.00839
5	4	2	←	4	3	1	6526.94761	0.00184
5	4	1	←	4	3	2	6532.77911	0.002
7	2	6	←	6	1	5	6650.91859	-0.00382
6	3	4	←	5	2	3	6717.29008	0.00373
8	1	7	←	7	2	6	7009.51644	-0.00356
6	3	3	←	5	2	4	7018.55356	0.00356
8	2	7	←	7	1	6	7417.21023	-0.00604
6	4	3	←	5	3	2	7425.65563	0.00415
6	4	2	←	5	3	3	7448.93862	0.00373
7	3	5	←	6	2	4	7504.14836	0.00393
3	3	0	←	2	2	0	4140.54508	0.00658
3	3	1	←	2	2	1	4148.01817	0.00515
4	2	3	←	3	1	3	4649.88998	0.00642
4	3	1	←	3	2	1	5035.58023	0.00415
4	3	2	←	3	2	2	5070.36493	0.00138
5	2	3	←	4	1	3	5352.78232	-0.00263
4	4	0	←	3	3	0	5619.74952	-0.02202
5	2	4	←	4	1	4	5666.04148	-0.00031
5	3	2	←	4	2	2	5913.0427	0.00151
5	3	3	←	4	2	3	6005.9117	-0.00563
5	4	1	←	4	3	1	6527.47434	0.00094
5	4	2	←	4	3	2	6532.25299	0.00351
6	3	3	←	5	2	3	6776.56362	-0.0018
6	3	4	←	5	2	4	6959.26928	-0.00165
6	4	2	←	5	3	2	7428.25535	0.00391
6	4	3	←	5	3	3	7446.33895	0.00401
7	1	6	←	6	0	6	7552.00597	-0.00982

7	3	4	←	6	2	4	7641.66206	-0.00524
7	2	6	←	6	1	6	7768.68581	0.00087
7	3	5	←	6	2	5	7934.06299	0.00526

Table S19 Observed rotational transitions (Obs.) and residuals (Obs.-Calc.) for the C1 sevoflurane – 2 water complex (in MHz).

J’	Ka’	Kc’	←	J’’	Ka’’	Kc’’	υ_{obs}	υ_{obs}-υ_{cal}
3	1	3	←	2	1	2	2627.5712	0.0119
4	0	4	←	3	0	3	3548.5162	-0.0048
4	2	3	←	3	2	2	3615.2501	-0.0063
4	2	2	←	3	2	1	3688.3158	-0.0097
4	1	3	←	3	1	2	3714.6869	0.0082
5	1	5	←	4	1	4	4358.3462	-0.0224
5	0	5	←	4	0	4	4398.7415	-0.0038
5	2	4	←	4	2	3	4508.8795	-0.0034
5	4	2	←	4	4	1	4545.2719	0.0198
5	4	1	←	4	4	0	4545.7326	0.0188
5	3	3	←	4	3	2	4548.1511	-0.0053
5	3	2	←	4	3	1	4563.3724	-0.0087
5	1	4	←	4	1	3	4622.8068	0.0173
5	2	3	←	4	2	2	4636.7395	0.0314
6	0	6	←	5	0	5	5243.1518	-0.006
6	2	5	←	5	2	4	5396.1145	0.0036
6	3	4	←	5	3	3	5459.4992	-0.0066
6	4	2	←	5	4	1	5461.7074	-0.0087
6	3	3	←	5	3	2	5497.7167	-0.0167
6	1	5	←	5	1	4	5514.2117	0.0251
6	2	4	←	5	2	3	5583.3031	-0.0043
9	1	8	←	8	3	5	5885.7424	0.0068
7	1	7	←	6	1	6	6071.9584	-0.0141
7	0	7	←	6	0	6	6087.2802	-0.007
7	2	6	←	6	2	5	6276.3161	0.0061
7	3	5	←	6	3	4	6368.0923	0.0015
7	4	4	←	6	4	3	6376.1141	-0.0028
7	4	3	←	6	4	2	6382.7246	-0.0069
7	3	4	←	6	3	3	6445.6357	-0.0232
7	2	5	←	6	2	4	6520.1554	0.0112
8	1	8	←	7	1	7	6924.6464	-0.0127
8	0	8	←	7	0	7	6932.952	-0.0073
8	2	7	←	7	2	6	7149.4152	0.0168
8	3	6	←	7	3	5	7271.9357	0.0095
8	5	3	←	7	5	2	7284.2306	-0.0002
8	4	5	←	7	4	4	7293.8265	0.0043
8	4	4	←	7	4	3	7311.1947	0.0023
8	3	5	←	7	3	4	7404.3508	-0.0121
9	0	9	←	8	0	8	7780.1167	0.0065

Table S20 Observed rotational transitions (Obs.) and residuals (Obs.-Calc.) for the C2 sevoflurane – 2 water complex (in MHz).

J’	Ka’	Kc’	←	J’’	Ka’’	Kc’’	υ_{obs}	υ_{obs}-υ_{cal}
4	1	4	←	3	1	3	3500.2234	-0.002
4	0	4	←	3	0	3	3553.0337	0.0026
4	2	3	←	3	2	2	3621.9524	-0.0006
4	2	2	←	3	2	1	3697.4488	-0.0022
4	1	3	←	3	1	2	3722.5014	0.0032
5	1	5	←	4	1	4	4364.0275	0
5	0	5	←	4	0	4	4403.7002	0.0044
5	2	4	←	4	2	3	4516.879	0.0058
5	3	3	←	4	3	2	4557.4977	0.0092
5	3	2	←	4	3	1	4573.6293	0.0019
5	1	4	←	4	1	3	4631.6802	0.009
5	2	3	←	4	2	2	4648.3834	-0.0066
6	1	6	←	5	1	5	5223.3208	-0.0323
6	0	6	←	5	0	5	5248.8275	0.0101
6	2	5	←	5	2	4	5405.1835	0.0093
6	3	4	←	5	3	3	5470.5764	-0.0265
6	4	3	←	5	4	2	5471.1079	0.0191
6	4	2	←	5	4	1	5473.2871	-0.0194
6	3	3	←	5	3	2	5511.0008	-0.0022
6	1	5	←	5	1	4	5523.4521	0.0166
6	2	4	←	5	2	3	5596.9541	-0.0036
7	1	7	←	6	1	6	6079.2265	0.0026
7	0	7	←	6	0	6	6093.8843	0.0063
7	2	6	←	6	2	5	6286.2445	0.0113
7	3	5	←	6	3	4	6380.7477	0.0083
7	1	6	←	6	1	5	6394.6453	-0.0292
7	4	3	←	6	4	2	6396.7918	0.0079
7	3	4	←	6	3	3	6462.2907	-0.0131
7	2	5	←	6	2	4	6535.0849	0.0034
8	1	8	←	7	1	7	6932.7227	0.0049
8	0	8	←	7	0	7	6940.5663	0.0042
8	1	7	←	7	1	6	7247.4209	-0.0136
8	7	2	←	7	7	1	7283.8983	0
8	3	6	←	7	3	5	7285.8552	0.0047
8	3	5	←	7	3	4	7424.1586	-0.0013
8	2	6	←	7	2	5	7457.609	0.0114
9	0	9	←	8	0	8	7788.7244	-0.0017

Table S21 Observed rotational transitions (Obs.) and residuals (Obs.-Calc.) for the C3 sevoflurane – 2 water complex (in MHz).

J'	Ka'	Kc'	←	J''	Ka''	Kc''	υ_{obs}	υ_{obs}-υ_{cal}
4	2	2	←	3	2	1	3698.5572	-0.0056
5	2	4	←	4	2	3	4521.1798	0.0208
5	3	3	←	4	3	2	4560.6356	-0.0011
5	3	2	←	4	3	1	4576.0067	0.0019
5	1	4	←	4	1	3	4635.1572	0.001
5	2	3	←	4	2	2	4649.5364	-0.0028
6	1	6	←	5	1	5	5231.1888	-0.0062
6	2	5	←	5	2	4	5410.7617	0
6	3	4	←	5	3	3	5474.4649	-0.0004
6	4	2	←	5	4	1	5476.7605	0.0067
6	1	5	←	5	1	4	5528.8089	0.0005
6	2	4	←	5	2	3	5598.6306	-0.0044
7	1	7	←	6	1	6	6088.615	-0.0037
7	0	7	←	6	0	6	6103.8084	-0.0019
7	2	6	←	6	2	5	6293.2987	-0.0045
7	3	5	←	6	3	4	6385.4923	-0.0033
7	4	4	←	6	4	3	6393.6381	-0.0177
7	4	3	←	6	4	2	6400.3675	0.0096
7	1	6	←	6	1	5	6402.4423	0
7	3	4	←	6	3	3	6463.691	0.0018
7	2	5	←	6	2	4	6537.8588	-0.0001
8	1	8	←	7	1	7	6943.6551	0.005
8	0	8	←	7	0	7	6951.8672	0.0014
8	2	7	←	7	2	6	7168.7123	0.004
8	1	7	←	7	1	6	7257.6689	-0.0053
8	3	6	←	7	3	5	7291.7328	-0.0003
8	4	5	←	7	4	4	7313.8907	0.0089
8	4	4	←	7	4	3	7331.467	-0.0074
8	2	6	←	7	2	5	7462.0961	0.0045

Table S22 Observed rotational transitions (Obs.) and residuals (Obs.-Calc.) for the C4 sevoflurane – 2 water complex (in MHz).

J'	Ka'	Kc'	←	J''	Ka''	Kc''	υ_{obs}	υ_{obs}-υ_{cal}
3	1	3	←	2	1	2	2627.5705	-0.0052
4	1	4	←	3	1	3	3495.4988	0.0014
4	0	4	←	3	0	3	3547.9505	-0.0065
4	2	2	←	3	2	1	3687.1332	-0.0076
4	1	3	←	3	1	2	3712.9873	0.0234
5	1	5	←	4	1	4	4358.5005	0.0042
5	0	5	←	4	0	4	4398.3013	-0.0029
5	2	4	←	4	2	3	4507.8423	0.0009
5	3	3	←	4	3	2	4546.951	0.0006
5	3	2	←	4	3	1	4562.1815	-0.0015
5	1	4	←	4	1	3	4620.6905	-0.0058
5	2	3	←	4	2	2	4634.9867	-0.0105
6	1	6	←	5	1	5	5217.1296	0.0069
6	0	6	←	5	0	5	5242.954	0.0002
6	2	5	←	5	2	4	5394.9168	0
6	3	4	←	5	3	3	5458.0238	0.0042
6	4	3	←	5	4	2	5458.2494	0.0003
6	4	2	←	5	4	1	5460.3003	-0.0015
6	3	3	←	5	3	2	5496.2444	0.0001
6	1	5	←	5	1	4	5511.7633	0.0005
6	2	4	←	5	2	3	5580.9711	-0.006
7	2	6	←	6	2	5	6275.0032	0.0047
7	3	5	←	6	3	4	6366.3107	-0.0022
7	4	4	←	6	4	3	6374.4143	-0.0013
7	4	3	←	6	4	2	6381.0627	0.0002
7	1	6	←	6	1	5	6383.0009	-0.0013
7	3	4	←	6	3	3	6443.8078	0.0089
7	2	5	←	6	2	4	6517.1671	-0.0012
5	3	2	←	4	1	3	6581.0809	0.0006
8	1	7	←	7	1	6	7236.0198	-0.0079
8	3	6	←	7	3	5	7269.8568	0.0015
8	2	6	←	7	2	5	7438.505	0.0015

Table S23 Observed rotational transitions (Obs.) and residuals (Obs.-Calc.) for the O1 sevoflurane – 2 water complex (in MHz).

J’	Ka’	Kc’	←	J’’	Ka’’	Kc’’	υ_{obs}	υ_{obs}-υ_{cal}
3	1	3	←	2	1	2	2572.9767	0.002
3	0	3	←	2	0	2	2628.7374	0.001
3	2	1	←	2	2	0	2683.6627	0.0012
3	1	2	←	2	1	1	2730.5089	-0.0008
4	1	4	←	3	1	3	3423.6508	0.0021
4	0	4	←	3	0	3	3477.4432	0.0002
4	2	3	←	3	2	2	3536.0727	0.0037
4	3	2	←	3	3	1	3554.1285	0.012
4	3	1	←	3	3	0	3557.6674	0.0042
4	2	2	←	3	2	1	3600.1566	-0.0028
4	1	3	←	3	1	2	3630.941	0.0017
5	1	5	←	4	1	4	4269.8548	0.0045
5	0	5	←	4	0	4	4312.7094	-0.0063
5	2	4	←	4	2	3	4411.287	0.0026
5	3	3	←	4	3	2	4445.6328	0.0003
5	3	2	←	4	3	1	4457.7196	-0.0056
5	1	4	←	4	1	3	4521.2864	-0.0037
5	2	3	←	4	2	2	4525.1705	-0.0078
6	1	6	←	5	1	5	5111.9508	0.0065
6	0	6	←	5	0	5	5141.2795	0.0042
6	2	5	←	5	2	4	5280.9225	0.0039
6	4	3	←	5	4	2	5335.9295	-0.0009
6	3	4	←	5	3	3	5336.7771	-0.0019
6	4	2	←	5	4	1	5337.4065	-0.0014
6	3	3	←	5	3	2	5367.4559	-0.0065
6	1	5	←	5	1	4	5397.4647	0.0046
6	2	4	←	5	2	3	5450.4504	-0.0077
7	1	7	←	6	1	6	5950.7131	0.008
7	0	7	←	6	0	6	5968.6819	0.0064
7	2	6	←	6	2	5	6144.3403	0.0041
7	3	5	←	6	3	4	6225.9005	-0.0035
7	4	4	←	6	4	3	6231.0751	-0.0026
7	4	3	←	6	4	2	6235.8795	-0.0068
7	1	6	←	6	1	5	6256.107	0.0081
7	3	4	←	6	3	3	6289.2695	-0.0035
8	1	8	←	7	1	7	6787.0472	0.0091
8	0	8	←	7	0	7	6797.257	0.0077
8	2	7	←	7	2	6	7001.3291	0.0031
8	1	7	←	7	1	6	7097.1509	0.0081
8	3	6	←	7	3	5	7111.2653	-0.0043
8	4	5	←	7	4	4	7127.6096	-0.0023

8	4	4	←	7	4	3	7140.3389	-0.0044
8	3	5	←	7	3	4	7222.1766	0.0015
8	2	6	←	7	2	5	7274.2572	-0.0004
9	1	9	←	8	1	8	7621.7619	0.0042
9	0	9	←	8	0	8	7627.2757	0.005
9	2	8	←	8	2	7	7852.1749	0.0027
9	1	8	←	8	1	7	7925.3429	0.0095
4	0	4	←	3	1	3	3312.0232	-0.0036
4	1	4	←	3	0	3	3589.0693	0.0044
6	2	5	←	5	3	2	3742.2257	-0.0288
5	1	4	←	4	2	3	3890.0107	0.0103
3	3	1	←	2	2	0	4119.1636	-0.0052
3	3	0	←	2	2	1	4126.8299	-0.0029
9	4	5	←	9	1	8	4173.1293	0.0061
5	0	5	←	4	1	4	4201.0995	0.0054
5	1	5	←	4	0	4	4381.472	0
6	1	5	←	5	2	4	4876.164	-0.012
4	3	2	←	3	2	1	4989.6231	-0.0007
4	3	1	←	3	2	2	5028.2815	-0.0107
5	2	4	←	4	1	3	5042.568	-0.0062
6	0	6	←	5	1	5	5072.5267	0.0077
6	1	6	←	5	0	5	5180.7021	0.0016
6	2	5	←	5	1	4	5802.1978	-0.0048
5	3	3	←	4	2	2	5835.0945	-0.0024
7	0	7	←	6	1	6	5929.2597	0.0094
7	1	7	←	6	0	6	5990.1311	0.0007
5	4	2	←	4	3	1	6479.5787	-0.0018
5	4	1	←	4	3	2	6484.1021	0.0014
6	3	4	←	5	2	3	6646.7005	0.0029
8	0	8	←	7	1	7	6775.8048	0.0104
8	1	7	←	7	2	6	6804.1666	0.0037
8	1	8	←	7	0	7	6808.4974	0.0044
6	3	3	←	5	2	4	6906.1181	-0.0084
8	2	7	←	7	1	6	7294.3089	0.0029
6	4	3	←	5	3	2	7357.7806	-0.0051
6	4	2	←	5	3	3	7375.8769	0.0008
9	0	9	←	8	1	8	7615.9956	-0.0314
9	1	9	←	8	0	8	7632.9847	-0.0167
3	3	1	←	2	2	1	4126.225	-0.0094
4	2	2	←	3	1	2	4360.8355	-0.0072
5	1	4	←	4	0	4	5155.9008	0.0251

Table S24 Observed rotational transitions (Obs.) and residuals (Obs.-Calc.) for the O2 sevoflurane – 2 water complex (in MHz).

J'	Ka'	Kc'	←	J''	Ka''	Kc''	υ_{obs}	υ_{obs}-υ_{cal}
3	1	3	←	2	1	2	2606.1475	0.0006
3	0	3	←	2	0	2	2661.8538	-0.0035
3	2	2	←	2	2	1	2692.8884	-0.0058
3	2	1	←	2	2	0	2723.9185	-0.0037
3	1	2	←	2	1	1	2769.4834	-0.0055
4	1	4	←	3	1	3	3467.0535	-0.0002
4	0	4	←	3	0	3	3518.8499	-0.0007
4	2	3	←	3	2	2	3584.2319	-0.0005
4	3	2	←	3	3	1	3604.5918	0.0086
4	3	1	←	3	3	0	3608.9952	0.0008
4	2	2	←	3	2	1	3655.8172	-0.0049
4	1	3	←	3	1	2	3681.4157	-0.0024
5	1	5	←	4	1	4	4323.1173	0.0028
5	0	5	←	4	0	4	4362.4616	-0.0009
5	2	4	←	4	2	3	4470.3074	0.0007
5	4	2	←	4	4	1	4505.9633	0.0048
5	4	1	←	4	4	0	4506.4192	0.0059
5	3	3	←	4	3	2	4508.7896	0.0003
5	3	2	←	4	3	1	4523.7428	-0.0033
5	1	4	←	4	1	3	4581.6079	-0.0015
5	2	3	←	4	2	2	4595.4753	-0.0102
6	1	6	←	5	1	5	5174.8706	0.0025
6	0	6	←	5	0	5	5200.4322	0.0015
6	2	5	←	5	2	4	5350.1149	0.0021
6	3	4	←	5	3	3	5412.2143	-0.0015
6	4	3	←	5	4	2	5412.4164	0.0022
6	4	2	←	5	4	1	5414.4211	-0.0044
6	3	3	←	5	3	2	5449.7516	-0.0063
6	1	5	←	5	1	4	5465.4146	0.0012
6	2	4	←	5	2	3	5533.3385	-0.009
7	1	7	←	6	1	6	6023.2692	0.003
7	0	7	←	6	0	6	6038.1494	0.0001
7	2	6	←	6	2	5	6223.0399	0.0029
7	3	5	←	6	3	4	6312.919	-0.0033
7	4	4	←	6	4	3	6320.847	0.0007
7	4	3	←	6	4	2	6327.3549	-0.0049
7	1	6	←	6	1	5	6329.706	0.0028
7	3	4	←	6	3	3	6389.0526	-0.0045
7	2	5	←	6	2	4	6461.6049	-0.0033
8	1	8	←	7	1	7	6869.333	0.0017
8	0	8	←	7	0	7	6877.3869	-0.0001

8	2	7	←	7	2	6	7089.0056	0.0011
8	1	7	←	7	1	6	7176.0274	-0.0014
8	3	6	←	7	3	5	7208.9639	-0.0017
8	5	4	←	7	5	3	7220.3021	0.0031
8	5	3	←	7	5	2	7221.1418	-0.0016
8	4	5	←	7	4	4	7230.5047	-0.0015
8	4	4	←	7	4	3	7247.6051	-0.0007
8	3	5	←	7	3	4	7338.8595	0.0021
8	2	6	←	7	2	5	7375.2745	0.0037
9	1	9	←	8	1	8	7713.9187	-0.0037
9	0	9	←	8	0	8	7718.0679	-0.0059
4	0	4	←	3	1	3	3371.0995	0.0056
4	1	4	←	3	0	3	3614.8134	0.0029
5	0	5	←	4	1	4	4266.5091	0.0064
5	1	5	←	4	0	4	4419.0763	0.0019
6	1	5	←	5	2	4	5000.0903	0.0071
6	0	6	←	5	1	5	5143.8238	0.0049
6	1	6	←	5	0	5	5231.4826	0.0026
7	1	6	←	6	2	5	5979.6787	0.0051
7	0	7	←	6	1	6	6007.1042	0.0043
10	5	5	←	9	6	4	6047.322	0.003
7	1	7	←	6	0	6	6054.3144	-0.0011
7	2	6	←	6	1	5	6573.0499	-0.0165
10	4	7	←	9	5	4	6597.4574	-0.0036
9	3	6	←	8	4	5	6597.657	-0.0017
8	0	8	←	7	1	7	6861.2238	0.0029
8	1	8	←	7	0	7	6885.4969	-0.0005
8	1	7	←	7	2	6	6932.6762	0.0106
9	0	9	←	8	1	8	7709.9679	0.0044
9	1	9	←	8	0	8	7722.0331	0.0002
7	1	6	←	6	2	4	5560.603	-0.0106
6	1	5	←	5	0	5	6322.3167	-0.0035
7	3	4	←	6	2	4	7540.7534	0.0126

Table S25 Observed rotational transitions (Obs.) and residuals (Obs.-Calc.) for the sevoflurane – 3 water complex (in MHz).

J’	Ka’	Kc’	←	J’’	Ka’’	Kc’’	υ_{obs}	υ_{obs}-υ_{cal}
3	1	3	←	2	1	2	2034.7077	0.0009
3	0	3	←	2	0	2	2082.2422	0.0003
3	2	2	←	2	2	1	2103.4667	-0.0031
3	2	1	←	2	2	0	2124.6925	-0.0009
3	1	2	←	2	1	1	2165.3732	-0.0022
4	1	4	←	3	1	3	2707.5005	0.0001
4	0	4	←	3	0	3	2754.6263	0
4	2	3	←	3	2	2	2800.366	-0.0023
4	3	2	←	3	3	1	2814.3334	-0.0042
4	3	1	←	3	3	0	2816.8685	-0.0063
4	2	2	←	3	2	1	2850.3195	-0.0047
4	1	3	←	3	1	2	2879.7514	-0.0043
5	1	5	←	4	1	4	3376.7283	0.0007
5	0	5	←	4	0	4	3415.6576	0.0008
5	2	4	←	4	2	3	3493.678	-0.0019
5	4	2	←	4	4	1	3517.8436	-0.0023
5	4	1	←	4	4	0	3518.063	-0.0038
5	3	3	←	4	3	2	3520.3988	-0.0031
5	3	2	←	4	3	1	3529.0815	-0.0043
5	2	3	←	4	2	2	3583.5411	-0.0061
5	1	4	←	4	1	3	3586.5251	-0.0041
6	1	6	←	5	1	5	4042.5991	0.001
6	0	6	←	5	0	5	4070.3262	0.0017
6	2	5	←	5	2	4	4182.6597	-0.002
6	5	2	←	5	5	1	4220.618	0.0054
6	5	1	←	5	5	0	4220.618	-0.0108
6	4	3	←	5	4	2	4225.0899	-0.0034
6	4	2	←	5	4	1	4226.0703	-0.0049
6	3	4	←	5	3	3	4226.3762	-0.0039
6	3	3	←	5	3	2	4248.5728	-0.0051
6	1	5	←	5	1	4	4282.5612	-0.0027
6	2	4	←	5	2	3	4318.2171	-0.0064
7	1	7	←	6	1	6	4705.6621	0.0014
7	0	7	←	6	0	6	4723.3423	0.0023
7	2	6	←	6	2	5	4866.7711	-0.0001
7	6	2	←	6	6	1	4923.4052	-0.0012
7	5	3	←	6	5	2	4927.4571	-0.0027
7	5	2	←	6	5	1	4927.545	-0.0034
7	3	5	←	6	3	4	4931.0421	-0.0023
7	4	4	←	6	4	3	4933.8944	-0.0027

7	4	3	←	6	4	2	4937.1011	-0.0033
7	1	6	←	6	1	5	4965.0136	-0.001
7	3	4	←	6	3	3	4977.4655	-0.006
7	2	5	←	6	2	4	5048.1949	-0.0057
8	1	8	←	7	1	7	5366.6064	0.0015
8	0	8	←	7	0	7	5377.0438	0.0021
8	2	7	←	7	2	6	5545.7623	0
8	7	2	←	7	7	1	5626.2507	-0.0011
8	6	3	←	7	6	2	5629.8441	0.0017
8	6	2	←	7	6	1	5629.8441	-0.0052
8	1	7	←	7	1	6	5633.0489	0.0021
8	5	4	←	7	5	3	5635.836	-0.0023
8	5	3	←	7	5	2	5636.184	-0.0042
8	4	5	←	7	4	4	5643.9445	-0.0024
8	4	4	←	7	4	3	5652.4839	-0.0041
8	3	5	←	7	3	4	5715.8108	-0.0067
8	2	6	←	7	2	5	5769.2383	-0.0052
9	1	9	←	8	1	8	6026.087	0.0005
9	0	9	←	8	0	8	6031.9316	0.001
9	2	8	←	8	2	7	6219.7495	0.0005
9	1	8	←	8	1	7	6289.3642	0.0026
9	8	1	←	8	8	0	6329.1302	0.0016
9	3	7	←	8	3	6	6331.1577	-0.0025
9	7	3	←	8	7	2	6332.3901	-0.0009
9	6	4	←	8	6	3	6337.5593	0.0345
9	6	3	←	8	6	2	6337.5593	0.0024
9	5	5	←	8	5	4	6345.8687	-0.0023
9	5	4	←	8	5	3	6346.9842	-0.0026
9	4	6	←	8	4	5	6354.5677	-0.0018
9	4	5	←	8	4	4	6374.078	-0.0052
9	3	6	←	8	3	5	6460.0011	-0.0072
9	2	7	←	8	2	6	6478.4143	-0.0036
10	1	10	←	9	1	9	6684.6293	-0.0009
10	0	10	←	9	0	9	6687.7821	-0.001
10	2	9	←	9	2	8	6889.1997	0.0005
10	1	9	←	9	1	8	6939.3333	0.0025
10	3	8	←	9	3	7	7024.37	-0.0008
10	9	1	←	9	9	0	7032.0262	0.0025
10	8	2	←	9	8	1	7035.05	0.0004
10	8	3	←	9	8	2	7035.05	0.0005
10	7	4	←	9	7	3	7039.5496	0.0006
10	6	5	←	9	6	4	7046.6108	-0.0006
10	6	4	←	9	6	3	7046.7267	-0.0035
10	5	6	←	9	5	5	7057.5553	-0.0026
10	5	5	←	9	5	4	7060.5954	-0.0025
10	4	7	←	9	4	6	7064.7357	-0.0027
10	4	6	←	9	4	5	7104.0087	-0.0058

10	2	8	←	9	2	7	7173.2191	-0.0007
10	3	7	←	9	3	6	7203.5389	-0.0075
11	1	11	←	10	1	10	7342.6033	-0.0028
11	0	11	←	10	0	10	7344.2587	-0.0027
11	2	10	←	10	2	9	7554.8469	0.0007
11	1	10	←	10	1	9	7587.9948	0.0017
11	3	9	←	10	3	8	7712.0373	-0.0012
11	9	2	←	10	9	1	7737.7789	0.0032
11	8	4	←	10	8	3	7741.8278	0.0025
11	7	5	←	10	7	4	7747.8557	0.0071
11	7	4	←	10	7	3	7747.8557	-0.0039
11	6	6	←	10	6	5	7757.2457	0.0001
11	6	5	←	10	6	4	7757.6178	-0.0011
11	5	7	←	10	5	6	7770.6992	-0.0005
11	4	8	←	10	4	7	7773.1716	-0.0027
11	5	6	←	10	5	5	7778.0147	-0.0021
11	4	7	←	10	4	6	7843.7111	-0.0054
11	2	9	←	10	2	8	7851.9001	0.002
11	3	8	←	10	3	7	7939.9134	-0.0037
11	10	1	←	10	10	0	7734.9323	0.0027
8	3	5	←	7	1	6	7545.2752	-0.0096
4	0	4	←	3	1	3	2600.3734	-0.0011
3	2	2	←	2	1	1	2823.6047	0.003
4	1	4	←	3	0	3	2861.7554	0.0032
5	1	4	←	4	2	3	3007.6778	-0.0125
5	0	5	←	4	1	4	3308.5286	-0.0022
3	3	1	←	2	2	0	3410.6942	-0.0024
3	3	0	←	2	2	1	3416.5644	0.0001
4	2	3	←	3	1	2	3458.5959	0.0012
5	1	5	←	4	0	4	3483.8566	0.0031
6	1	5	←	5	2	4	3796.5637	-0.0105
6	0	6	←	5	1	5	4002.1262	-0.0014
5	2	4	←	4	1	3	4072.5271	0.0081
4	3	2	←	3	2	1	4100.341	0.0002
6	1	6	←	5	0	5	4110.8034	0.0085
4	3	1	←	3	2	2	4129.9678	-0.0013
7	1	6	←	6	2	5	4578.9185	-0.0087
4	4	0	←	3	3	1	4638.8951	-0.0014
4	4	1	←	3	3	0	4638.4438	0.0022
6	2	5	←	5	1	4	4668.6586	0.0072
7	0	7	←	6	1	6	4682.8737	0.0042
7	1	7	←	6	0	6	4746.1348	0.0036
5	3	3	←	4	2	2	4770.421	0.0024
9	3	6	←	8	4	5	4788.7261	0.1149
8	2	6	←	7	3	5	4791.3555	-0.0164
5	3	2	←	4	2	3	4858.6856	-0.0011
7	2	6	←	6	1	5	5252.8676	0.0089

5	4	2	←	4	3	1	5339.4152	0.0025
5	4	1	←	4	3	2	5342.6282	0.0023
8	1	7	←	7	2	6	5345.1941	-0.0086
8	0	8	←	7	1	7	5354.2513	0.0009
8	1	8	←	7	0	7	5389.3992	0.0031
6	3	4	←	5	2	3	5413.2571	0.0057
6	3	3	←	5	2	4	5613.5791	-0.0056
8	2	7	←	7	1	6	5833.6213	0.015
9	0	9	←	8	1	8	6019.5767	0.0005
6	4	3	←	5	3	2	6035.4235	0.0033
9	1	9	←	8	0	8	6038.442	0.001
6	4	2	←	5	3	3	6048.2991	0
7	3	4	←	6	2	5	6408.385	-0.0094
9	2	8	←	8	1	7	6420.3192	0.0106
10	2	8	←	9	3	7	6478.786	-0.0106
6	5	2	←	5	4	1	6566.1128	-0.0042
6	5	1	←	5	4	2	6566.3956	0.012
8	3	6	←	7	2	5	6610.9372	0.0126
7	2	5	←	6	1	6	6620.1899	0.064
10	0	10	←	9	1	9	6681.2712	-0.0015
10	1	10	←	9	0	9	6691.1389	-0.0017
7	4	4	←	6	3	3	6720.743	0.0036
7	4	3	←	6	3	4	6759.0252	0.0017
10	1	9	←	9	2	8	6808.3816	-0.0022
10	2	9	←	9	1	8	7020.1575	0.0115
8	3	5	←	7	2	6	7257.4219	-0.0189
11	2	9	←	10	3	8	7306.3253	0.0015
11	0	11	←	10	1	10	7340.9002	-0.0037
11	1	11	←	10	0	10	7345.9612	-0.0024
8	4	5	←	7	3	4	7387.2233	0.0085
8	4	4	←	7	3	5	7480.4665	-0.0004
11	1	10	←	10	2	9	7507.1746	-0.003
11	2	10	←	10	1	9	7635.6661	0.0046
3	3	0	←	2	2	0	3411.1242	0.0002
3	3	1	←	2	2	1	3416.136	-0.001
4	2	2	←	3	1	2	3535.2139	-0.0004
6	1	5	←	5	2	3	3630.0846	-0.0025
4	2	3	←	3	1	3	3720.3599	-0.0007
4	3	1	←	3	2	1	4103.3061	0.0008
4	3	2	←	3	2	2	4127.0027	-0.0019
5	2	3	←	4	1	3	4239.0039	-0.0021
7	1	6	←	6	2	4	4276.8855	0.0072
5	2	4	←	4	1	4	4506.5316	-0.0086
5	3	2	←	4	2	2	4782.0684	0.0014
5	3	3	←	4	2	3	4847.0369	-0.0014
8	1	7	←	7	2	5	4861.7094	-0.015
6	2	4	←	5	1	4	4970.6945	-0.0057

6	2	5	←	5	1	5	5312.4668	-0.0076
5	4	1	←	4	3	1	5339.6639	0.0025
5	4	2	←	4	3	2	5342.3785	0.0013
6	3	3	←	5	2	3	5447.1018	0.0041
6	3	4	←	5	2	4	5579.7357	-0.0027
7	2	5	←	6	1	5	5736.3292	-0.0077
6	4	2	←	5	3	2	6036.6546	0.0039
6	4	3	←	5	3	3	6047.0701	0.0015
7	3	4	←	6	2	4	6106.3497	0.0042
7	2	6	←	6	1	6	6136.6488	0.0012
10	2	8	←	9	3	6	6186.9058	-0.0047
7	3	5	←	6	2	5	6328.1167	-0.0045
8	2	6	←	7	1	6	6540.5522	-0.0136
7	4	3	←	6	3	3	6725.1808	0.0036
7	4	4	←	6	3	4	6754.6146	0.029
8	3	5	←	7	2	5	6773.9619	-0.0005
8	2	7	←	7	1	7	6976.7395	-0.0097
8	3	6	←	7	2	6	7094.3981	-0.0048
7	5	2	←	6	4	2	7267.6167	0.0086
7	5	3	←	6	4	3	7268.7346	0.0024
9	2	7	←	8	1	7	7385.9178	-0.0192
9	3	6	←	8	2	6	7464.7197	-0.0075
8	4	5	←	7	3	5	7467.4898	0.0017
9	3	7	←	8	2	7	7879.7888	-0.0119
8	5	3	←	7	4	3	7966.698	0.0061

Table S26 Observed rotational transitions (Obs.) and residuals (Obs.-Calc.) for the C1 sevoflurane – 3 water complex (in MHz).

J’	Ka’	Kc’	←	J’’	Ka’’	Kc’’	υ_{obs}	υ_{obs}-υ_{cal}
5	0	5	←	4	0	4	3415.0914	0.0058
5	1	5	←	4	1	4	3376.1313	-0.0032
5	2	4	←	4	2	3	3493.009	0.0039
5	1	4	←	4	1	3	3585.8141	-0.0005
6	1	6	←	5	1	5	4041.8976	-0.0002
6	0	6	←	5	0	5	4069.6568	0.0018
6	1	5	←	5	1	4	4281.7476	0.0002
6	2	4	←	5	2	3	4317.2684	0
7	1	7	←	6	1	6	4704.8572	0.0024
7	0	7	←	6	0	6	4722.5624	-0.001
7	2	6	←	6	2	5	4865.8685	0.003
7	3	5	←	6	3	4	4930.0501	0.0004
7	1	6	←	6	1	5	4964.1182	-0.0007
7	3	4	←	6	3	3	4976.3614	-0.0029
7	2	5	←	6	2	4	5047.114	0.0041
8	1	8	←	7	1	7	5365.6875	-0.0056
8	0	8	←	7	0	7	5376.1541	0.0012
8	3	6	←	7	3	5	5631.9313	0.0015
8	1	7	←	7	1	6	5632.0882	0.0025
9	1	9	←	8	1	8	6025.0691	0.0007
9	0	9	←	8	0	8	6030.932	0.0038
9	2	8	←	8	2	7	6218.6315	-0.0057
9	1	8	←	8	1	7	6288.3263	-0.0042
9	3	7	←	8	3	6	6329.9206	0.0027
10	1	10	←	9	1	9	6683.5043	-0.0004
10	0	10	←	9	0	9	6686.6632	-0.0044
10	2	9	←	9	2	8	6887.9854	-0.004
10	1	9	←	9	1	8	6938.212	0.0009
10	2	8	←	9	2	7	7171.8607	-0.0061
11	2	10	←	10	2	9	7553.5425	0.0037
11	1	10	←	10	1	9	7586.7702	0.0042

Table S27 Observed rotational transitions (Obs.) and residuals (Obs.-Calc.) for the C2 sevoflurane – 3 water complex (in MHz).

J’	Ka’	Kc’	←	J’’	Ka’’	Kc’’	υ_{obs}	υ_{obs}-υ_{cal}
5	1	5	←	4	1	4	3368.2103	0.0026
5	2	4	←	4	2	3	3485.1338	-0.0022
5	2	3	←	4	2	2	3574.9214	-0.0065
5	1	4	←	4	1	3	3577.9928	0.0029
6	1	6	←	5	1	5	4032.3769	-0.0039
6	0	6	←	5	0	5	4060.1802	0.0315
6	2	5	←	5	2	4	4172.4162	-0.0033
6	1	5	←	5	1	4	4272.346	0.0004
6	2	4	←	5	2	3	4307.8968	0.0058
7	1	7	←	6	1	6	4693.7418	-0.0042
7	0	7	←	6	0	6	4711.4797	0.0187
7	2	6	←	6	2	5	4854.8373	0.0018
7	1	6	←	6	1	5	4953.1367	0.0041
8	0	8	←	7	0	7	5363.4509	-0.004
8	2	7	←	7	2	6	5532.134	-0.0026
8	3	6	←	7	3	5	5619.3635	0.002
8	1	7	←	7	1	6	5619.5016	-0.0058
9	1	9	←	8	1	8	6010.7501	-0.0238
9	0	9	←	8	0	8	6016.6262	-0.0092
9	1	8	←	8	1	7	6274.1545	-0.0011
10	1	10	←	9	1	9	6667.6104	-0.0063
10	0	10	←	9	0	9	6670.7739	-0.0065
10	1	9	←	9	1	8	6922.438	0.0004
11	1	11	←	10	1	10	7323.9164	0.0257
11	1	10	←	10	1	9	7569.3873	-0.0071

Table S28 Observed rotational transitions (Obs.) and residuals (Obs.-Calc.) for the C3 sevoflurane – 3 water complex (in MHz).

J’	Ka’	Kc’	←	J’’	Ka’’	Kc’’	υ_{obs}	υ_{obs}-υ_{cal}
5	1	5	←	4	1	4	3372.0327	0.0078
5	2	3	←	4	2	2	3581.1882	-0.0002
5	1	4	←	4	1	3	3583.1963	-0.002
6	1	6	←	5	1	5	4036.8213	-0.0016
6	0	6	←	5	0	5	4064.1911	0.0006
6	2	5	←	5	2	4	4177.9485	0
6	1	5	←	5	1	4	4278.0818	-0.0104
6	2	4	←	5	2	3	4315.3574	0.0019
7	2	6	←	6	2	5	4861.0294	-0.0073
7	6	2	←	6	6	1	4918.7617	0.0053
7	3	5	←	6	3	4	4926.3261	-0.0059
7	4	3	←	6	4	2	4932.7699	0.0004
7	1	6	←	6	1	5	4959.1496	0.0068
7	3	4	←	6	3	3	4974.0632	0.0004
7	2	5	←	6	2	4	5044.5725	-0.0027
8	1	8	←	7	1	7	5358.6541	-0.0004
8	0	8	←	7	0	7	5368.8221	-0.0067
8	2	7	←	7	2	6	5538.9349	0.0018
8	1	7	←	7	1	6	5625.6185	-0.0008
8	3	6	←	7	3	5	5627.5134	0.0052
8	4	5	←	7	4	4	5638.8914	-0.0005
8	2	6	←	7	2	5	5764.6037	0.0021
9	1	9	←	8	1	8	6017.0553	-0.0022
9	0	9	←	8	0	8	6022.72	-0.0016
9	2	8	←	8	2	7	6211.7704	-0.0001
9	1	8	←	8	1	7	6280.4423	0.0044
9	3	7	←	8	3	6	6324.6741	-0.0007
10	1	10	←	9	1	9	6674.5356	0.0015
10	0	10	←	9	0	9	6677.5605	-0.0122
11	1	11	←	10	1	10	7331.4672	0.0139

Table S29 Observed rotational transitions (Obs.) and residuals (Obs.-Calc.) for the C4 sevoflurane – 3 water complex (in MHz).

J’	Ka’	Kc’	←	J’’	Ka’’	Kc’’	υ_{obs}	υ_{obs}-υ_{cal}
5	1	5	←	4	1	4	3372.9731	0.0051
5	0	5	←	4	0	4	3411.6044	0.0023
5	2	3	←	4	2	2	3573.1834	-0.0106
6	1	6	←	5	1	5	4038.4257	0.004
6	0	6	←	5	0	5	4066.1812	0.0015
6	2	5	←	5	2	4	4174.7307	0.0053
6	2	4	←	5	2	3	4305.5454	0.0049
7	1	7	←	6	1	6	4701.1361	0.0008
7	0	7	←	6	0	6	4718.9894	-0.0018
7	2	6	←	6	2	5	4858.0439	0.0003
7	3	5	←	6	3	4	4919.8732	-0.0013
7	4	3	←	6	4	2	4925.3325	-0.0076
7	4	4	←	6	4	3	4922.3828	0.0147
7	1	6	←	6	1	5	4954.8193	-0.0033
7	2	5	←	6	2	4	5033.5975	-0.0013
8	1	8	←	7	1	7	5361.7693	0.0036
8	0	8	←	7	0	7	5372.3965	0.0001
9	1	9	←	8	1	8	6020.9484	0.0023
9	0	9	←	8	0	8	6026.943	-0.0039
9	2	8	←	8	2	7	6209.9646	-0.0006
9	1	8	←	8	1	7	6279.7001	-0.0022
10	1	10	←	9	1	9	6679.1813	-0.0069
10	0	10	←	9	0	9	6682.4504	-0.0008
10	2	9	←	9	2	8	6879.0787	-0.003
10	1	9	←	9	1	8	6929.8201	0.0037

Table S30 Observed rotational transitions (Obs.) and residuals (Obs.-Calc.) for the O1 sevoflurane – 3 water complex (in MHz).

J'	Ka'	Kc'	←	J''	Ka''	Kc''	υ_{obs}	υ_{obs}-υ_{cal}
3	0	3	←	2	0	2	2032.8227	-0.0047
3	1	2	←	2	1	1	2110.5403	-0.0061
4	1	4	←	3	1	3	2644.0429	-0.0034
4	0	4	←	3	0	3	2690.8932	-0.0047
4	2	3	←	3	2	2	2731.7507	-0.007
4	2	2	←	3	2	1	2776.3353	-0.0048
4	1	3	←	3	1	2	2807.5753	-0.0047
5	1	5	←	4	1	4	3298.1605	-0.0044
5	0	5	←	4	0	4	3338.1203	-0.0029
5	2	4	←	4	2	3	3408.697	-0.0046
5	3	3	←	4	3	2	3432.5241	-0.0042
5	3	2	←	4	3	1	3439.6933	-0.0089
5	2	3	←	4	2	2	3489.7619	-0.0069
5	1	4	←	4	1	3	3498.0111	-0.0044
6	1	6	←	5	1	5	3949.1544	-0.005
6	0	6	←	5	0	5	3978.6696	-0.0007
6	2	5	←	5	2	4	4081.7896	-0.0057
6	4	3	←	5	4	2	4119.3373	-0.006
6	3	4	←	5	3	3	4120.9723	-0.0068
6	3	3	←	5	3	2	4139.4177	-0.0082
6	1	5	←	5	1	4	4179.1219	-0.0051
6	2	4	←	5	2	3	4205.5723	-0.0047
7	1	7	←	6	1	6	4597.4677	-0.0023
7	0	7	←	6	0	6	4616.9865	-0.005
7	2	6	←	6	2	5	4750.517	-0.0036
7	3	5	←	6	3	4	4808.4642	-0.0065
7	1	6	←	6	1	5	4848.2529	0.0026
7	2	5	←	6	2	4	4918.0918	-0.0055
8	1	8	←	7	1	7	5243.6875	-0.0038
8	0	8	←	7	0	7	5255.6268	-0.0038
8	2	7	←	7	2	6	5414.595	-0.0009
8	3	6	←	7	3	5	5493.8235	-0.0012
8	5	4	←	7	5	3	5494.4871	-0.0162
8	5	3	←	7	5	2	5494.7378	-0.016
8	4	5	←	7	4	4	5502.025	-0.0049
8	1	7	←	7	1	6	5504.0859	-0.0026
8	4	4	←	7	4	3	5508.6322	-0.0038
8	2	6	←	7	2	5	5623.2109	0.0017
8	3	5	←	7	3	4	5564.3965	0.0014
9	1	9	←	8	1	8	5888.4182	-0.0021
9	0	9	←	8	0	8	5895.3338	-0.0017

9	2	8	←	8	2	7	6074.0394	0.0015
9	1	8	←	8	1	7	6148.1953	0.0039
9	3	7	←	8	3	6	6175.9089	0.0034
9	5	5	←	8	5	4	6186.242	-0.008
9	5	4	←	8	5	3	6187.0509	0.0001
9	4	6	←	8	4	5	6194.7076	0.0003
9	4	5	←	8	4	4	6209.9103	0.0006
9	2	7	←	8	2	6	6318.1378	0.0074
10	1	10	←	9	1	9	6532.166	0.0085
10	0	10	←	9	0	9	6536.0108	-0.0012
10	2	9	←	9	2	8	6729.1791	0.006
10	1	9	←	9	1	8	6784.877	0.0021
10	3	8	←	9	3	7	6853.7675	0.007
10	6	4	←	9	6	3	6869.7609	-0.0102
10	5	6	←	9	5	5	6879.5247	0.0048
10	5	5	←	9	5	4	6881.703	-0.0069
10	4	7	←	9	4	6	6887.2865	0.0041
10	4	6	←	9	4	5	6918.2282	0.0005
10	2	8	←	9	2	7	7000.5715	0.0065
10	3	7	←	9	3	6	7012.861	0.014
11	1	11	←	10	1	10	7175.2741	0.0016
11	0	11	←	10	0	10	7177.3626	0.0012
11	2	10	←	10	2	9	7380.5903	0.0054
11	1	10	←	10	1	9	7419.0052	0.0097
11	3	9	←	10	3	8	7526.7167	0.0037
11	5	7	←	10	5	6	7574.2016	0.0007
11	4	8	←	10	4	7	7578.6903	0.0052
11	4	7	←	10	4	6	7635.2125	0.0101
11	2	9	←	10	2	8	7668.6511	0.0131
11	3	8	←	10	3	7	7733.0763	0.015
12	1	12	←	11	1	11	7818.0113	-0.0016
12	0	12	←	11	0	11	7819.1335	0.0123
7	0	7	←	6	1	6	4570.3825	-0.0025
7	1	7	←	6	0	6	4644.0915	0.0149
8	0	8	←	7	1	7	5228.5506	0.0049
8	1	8	←	7	0	7	5270.7682	-0.008
9	0	9	←	8	1	8	5880.1851	-0.0049
9	1	9	←	8	0	8	5903.56	-0.0059
5	3	3	←	4	0	4	5909.6745	0.0196
9	1	8	←	8	2	7	5919.7763	0.0139
6	4	3	←	5	3	2	5964.1166	-0.0038
6	4	2	←	5	3	3	5974.6663	-0.0135
11	3	8	←	10	4	7	6226.9023	-0.0076
10	0	10	←	9	1	9	6527.7769	-0.0048
10	1	10	←	9	0	9	6540.3889	0.001
5	0	5	←	4	1	4	3222.0503	0.0029
4	3	2	←	3	2	1	4055.2668	-0.0104

4	3	1	←	3	2	2	4081.3185	-0.0123
5	3	3	←	4	2	2	4711.4595	-0.006
5	3	2	←	4	2	3	4789.2732	-0.0021
6	3	3	←	5	2	4	5520.0043	0.0046
5	5	1	←	4	4	0	5817.4618	0.0055
7	3	5	←	6	2	4	5945.5655	-0.004
7	4	4	←	6	3	3	6634.7494	-0.017
6	6	0	←	5	5	1	7034.0125	-0.0025
8	5	4	←	7	4	3	7868.6974	0.014
5	5	0	←	4	4	0	5817.4618	0.0043
6	6	1	←	5	5	1	7034.0125	-0.0025

Table S31 Observed rotational transitions (Obs.) and residuals (Obs.-Calc.) for the O2 sevoflurane – 3 water complex (in MHz).

J'	Ka'	Kc'	←	J''	Ka''	Kc''	υ_{obs}	υ_{obs}-υ_{cal}
3	1	2	←	2	1	1	2128.1051	0.0063
4	1	4	←	3	1	3	2660.9401	0.0015
4	0	4	←	3	0	3	2708.248	-0.0003
4	2	3	←	3	2	2	2752.104	-0.0038
4	2	2	←	3	2	1	2799.995	0.0066
4	1	3	←	3	1	2	2830.4218	-0.0022
5	1	5	←	4	1	4	3318.8122	0.0001
5	0	5	←	4	0	4	3358.4382	-0.0006
5	2	4	←	4	2	3	3433.6621	-0.0004
5	3	3	←	4	3	2	3459.2602	-0.0027
5	3	2	←	4	3	1	3467.3076	-0.0084
5	2	3	←	4	2	2	3520.1998	-0.0002
5	1	4	←	4	1	3	3525.5453	-0.0027
6	1	6	←	5	1	5	3973.4072	-0.0019
6	0	6	←	5	0	5	4002.1227	0.0444
6	2	5	←	5	2	4	4111.0731	-0.0008
6	4	3	←	5	4	2	4151.5698	-0.0214
6	4	2	←	5	4	1	4152.4762	0.0023
6	3	4	←	5	3	3	4153.0537	0.0022
6	3	3	←	5	3	2	4173.6893	-0.0019
6	1	5	←	5	1	4	4210.4914	-0.0076
6	2	4	←	5	2	3	4242.2949	-0.0021
7	1	7	←	6	1	6	4625.2325	-0.0012
7	0	7	←	6	0	6	4643.8018	-0.0034
7	2	6	←	6	2	5	4783.8065	0.0006
7	3	5	←	6	3	4	4845.6804	0.0015
7	4	4	←	6	4	3	4847.9772	0.0016
7	4	3	←	6	4	2	4850.8631	0.001
7	1	6	←	6	1	5	4882.4845	-0.0005
7	3	4	←	6	3	3	4889.0428	-0.0018
7	2	5	←	6	2	4	4960.2925	-0.0001
8	1	8	←	7	1	7	5274.9379	0.0001
8	0	8	←	7	0	7	5286.0687	-0.0003
8	2	7	←	7	2	6	5451.5952	0.0019
8	3	6	←	7	3	5	5535.8704	0.0031
8	5	4	←	7	5	3	5537.6987	-0.0011
8	5	3	←	7	5	2	5538.0118	0.0066
8	1	7	←	7	1	6	5540.4377	-0.001
8	4	5	←	7	4	4	5545.6116	0.0017
8	4	4	←	7	4	3	5553.3123	0.0001
8	3	5	←	7	3	4	5613.7042	0.0015
8	2	6	←	7	2	5	5669.9751	0.0005
9	1	9	←	8	1	8	5923.1497	-0.0067

9	0	9	←	8	0	8	5929.4774	-0.0029
9	2	8	←	8	2	7	6114.5054	-0.0002
9	1	8	←	8	1	7	6186.5565	0.002
9	3	7	←	8	3	6	6222.4114	0.0014
9	5	5	←	8	5	4	6235.1993	-0.026
9	5	4	←	8	5	3	6236.2038	0.0039
9	4	6	←	8	4	5	6243.8984	0.0071
9	4	5	←	8	4	4	6261.5513	0.0057
9	3	6	←	8	3	5	6344.6244	0.0018
10	1	10	←	9	1	9	6570.4064	-0.0011
10	0	10	←	9	0	9	6573.8635	-0.004
10	2	9	←	9	2	8	6772.9495	-0.0006
10	1	9	←	9	1	8	6825.7899	-0.0001
10	3	8	←	9	3	7	6904.3274	0.0048
10	4	7	←	9	4	6	6941.8857	0.0064
10	4	6	←	9	4	5	6977.6009	0.0081
10	2	8	←	9	2	7	7053.3297	0.0028
10	3	7	←	9	3	6	7075.8106	0.0069
11	1	11	←	10	1	10	7217.059	-0.005
11	0	11	←	10	0	10	7218.8996	-0.0059
11	2	10	←	10	2	9	7427.6034	0.0053
11	1	10	←	10	1	9	7463.1726	0.0017
11	3	9	←	10	3	8	7580.9281	-0.0104
11	5	7	←	10	5	6	7634.9445	0.0015
4	2	3	←	3	1	2	3422.7511	-0.0092
6	1	5	←	5	2	4	3710.0549	0.0068
6	0	6	←	5	1	5	3930.1383	0.0022
6	1	6	←	5	0	5	4045.3493	-0.0019
4	3	2	←	3	2	1	4069.8167	-0.0084
4	3	1	←	3	2	2	4098.0323	-0.0074
7	0	7	←	6	1	6	4600.5345	0.0023
8	1	7	←	7	2	6	5238.0813	-0.0107
8	0	8	←	7	1	7	5261.3653	-0.0021
9	0	9	←	8	1	8	5915.9071	-0.0029
9	1	9	←	8	0	8	5936.729	0.0022
7	3	5	←	6	2	4	5965.3737	0.0407
9	1	8	←	8	2	7	5973.0605	0.0074
6	4	3	←	5	3	2	5987.3752	0.0024
6	4	2	←	5	3	3	5999.2852	0.0082
10	0	10	←	9	1	9	6566.6104	-0.0107
10	1	10	←	9	0	9	6577.6495	-0.0043
10	1	9	←	9	2	8	6684.3281	-0.0093
9	4	6	←	8	3	5	7948.4124	0.0014
5	5	0	←	4	4	0	5834.106	-0.0052
5	5	1	←	4	4	1	5834.106	-0.0288
6	4	2	←	5	3	2	5988.4727	-0.0059

Table S32 Observed rotational transitions (Obs.) and residuals (Obs.-Calc.) for the O3 sevoflurane – 3 water complex (in MHz).

J'	Ka'	Kc'	←	J''	Ka''	Kc''	υ_{obs}	υ_{obs}-υ_{cal}
3	0	3	←	2	0	2	2066.2468	0.0036
4	1	4	←	3	1	3	2684.7917	-0.0015
4	0	4	←	3	0	3	2731.6815	-0.0016
4	2	3	←	3	2	2	2781.1583	-0.0047
4	2	2	←	3	2	1	2835.2458	0.0023
4	1	3	←	3	1	2	2862.789	-0.003
5	1	5	←	4	1	4	3347.7732	-0.002
5	0	5	←	4	0	4	3385.5234	0.003
5	2	4	←	4	2	3	3469.0521	-0.0036
5	3	3	←	4	3	2	3498.0117	-0.0041
5	3	2	←	4	3	1	3507.9876	-0.0045
5	1	4	←	4	1	3	3563.9391	-0.0061
5	2	3	←	4	2	2	3565.4943	-0.0015
6	1	6	←	5	1	5	4007.2428	-0.0017
6	0	6	←	5	0	5	4033.3606	-0.0019
6	2	5	←	5	2	4	4152.239	-0.0077
6	4	3	←	5	4	2	4198.5042	-0.007
6	3	4	←	5	3	3	4199.4244	-0.0045
6	4	2	←	5	4	1	4199.6861	-0.0183
6	3	3	←	5	3	2	4224.7926	-0.0057
6	1	5	←	5	1	4	4253.2249	-0.0027
6	2	4	←	5	2	3	4296.3363	-0.0041
7	1	7	←	6	1	6	4663.8375	-0.0013
7	0	7	←	6	0	6	4680.0166	-0.0021
7	2	6	←	6	2	5	4830.1823	-0.0025
7	3	5	←	6	3	4	4899.2173	-0.001
7	4	3	←	6	4	2	4907.0985	-0.0044
7	1	6	←	6	1	5	4927.7304	-0.0002
7	3	4	←	6	3	3	4951.8045	-0.0036
7	2	5	←	6	2	4	5021.3338	0.0012
8	1	8	←	7	1	7	5318.3205	0.0026
8	0	8	←	7	0	7	5327.6115	0.0023
8	2	7	←	7	2	6	5502.6672	0.0008
8	1	7	←	7	1	6	5587.1516	0.0007
8	6	3	←	7	6	2	5594.1302	-0.0078
8	6	2	←	7	6	1	5594.1302	-0.0173
8	3	6	←	7	3	5	5595.9209	0.0009
8	5	4	←	7	5	3	5600.669	-0.0025
8	5	3	←	7	5	2	5601.1088	-0.0113
8	4	5	←	7	4	4	5609.1462	0.0005
8	4	4	←	7	4	3	5619.4589	0.001
8	3	5	←	7	3	4	5688.4532	0.0007
8	2	6	←	7	2	5	5736.1788	-0.0027

9	1	9	←	8	1	8	5971.3795	0.0037
9	0	9	←	8	0	8	5976.446	0.0032
9	2	8	←	8	2	7	6169.9024	0.005
9	1	8	←	8	1	7	6235.1993	-0.0016
9	3	7	←	8	3	6	6288.2205	0.0051
9	5	5	←	8	5	4	6306.7793	-0.0044
9	5	4	←	8	5	3	6308.2021	-0.0086
9	4	6	←	8	4	5	6315.4839	-0.002
9	4	5	←	8	4	4	6338.8955	0.0002
9	3	6	←	8	3	5	6429.9943	0.0021
9	2	7	←	8	2	6	6437.8203	0.0011
10	1	10	←	9	1	9	6623.5583	0.0103
10	0	10	←	9	0	9	6626.2206	0.008
10	2	9	←	9	2	8	6832.4722	0.0042
10	1	9	←	9	1	8	6877.9556	0.0092
10	3	8	←	9	3	7	6975.1083	0.0121
10	7	4	←	9	7	3	6995.13	-0.0083
10	7	3	←	9	7	2	6995.13	-0.012
10	4	7	←	9	4	6	7021.0431	0.0049
10	4	6	←	9	4	5	7067.6915	0.0077
10	2	8	←	9	2	7	7123.6273	0.0106
11	1	11	←	10	1	10	7275.2044	0.0057
11	0	11	←	10	0	10	7276.5729	0.0099
11	1	10	←	10	1	9	7520.3417	0.0087
11	2	9	←	10	2	8	7792.1105	0.004
5	0	5	←	4	1	4	3285.7434	-0.0091
6	1	6	←	5	0	5	4069.263	-0.0043
9	2	8	←	8	3	5	4444.9364	-0.0113
7	1	6	←	6	2	5	4566.5464	-0.0079
7	0	7	←	6	1	6	4644.0914	-0.0226
7	1	7	←	6	0	6	4699.7471	0.0035
5	3	3	←	4	2	2	4717.4884	-0.0299
5	4	2	←	4	3	1	5280.1067	-0.0138
8	0	8	←	7	1	7	5307.8863	0.0019
8	1	8	←	7	0	7	5338.0402	-0.0023
9	0	9	←	8	1	8	5966.01	0.0007
9	1	9	←	8	0	8	5981.8161	0.0068
6	4	2	←	5	3	3	5985.53	0.0028
10	1	10	←	9	0	9	6628.916	0.0016
7	4	4	←	6	3	3	6649.0758	0.0194
10	1	9	←	9	2	8	6764.1115	0.0076
10	2	9	←	9	1	8	6946.3069	-0.0035
8	3	5	←	7	2	6	7227.6335	0.0174
11	0	11	←	10	1	10	7273.8603	-0.0008
4	3	2	←	3	2	2	4083.7889	-0.011
8	2	7	←	7	1	7	6941.234	-0.0006

Table S33 Observed rotational transitions (Obs.) and residuals (Obs.-Calc.) for the sevoflurane – 4 water Clockwise complex (in MHz).

J’	Ka’	Kc’	←	J’’	Ka’’	Kc’’	υ_{obs}	υ_{obs}-υ_{cal}
5	0	5	←	4	1	4	3342.449	-0.0093
5	1	5	←	4	1	4	3357.663	0.0016
5	0	5	←	4	0	4	3373.733	0.0013
5	1	5	←	4	0	4	3388.938	0.0029
5	2	4	←	4	2	3	3467.392	0
5	4	2	←	4	4	1	3504.643	0.0043
5	4	1	←	4	4	0	3505.608	0.0012
5	3	2	←	4	3	1	3526.503	-0.0018
4	3	2	←	3	2	2	3527.301	-0.0005
4	3	1	←	3	2	2	3534.946	-0.0004
5	1	4	←	4	1	3	3538.63	0.0007
5	2	3	←	4	2	2	3578.691	-0.0013
4	4	0	←	3	3	1	3798.315	0.0027
4	4	1	←	3	3	1	3798.185	-0.0036
4	4	1	←	3	3	0	3797.067	-0.0009
4	4	0	←	3	3	0	3797.193	0.0015
6	0	6	←	5	1	5	4011.505	0.0014
6	1	6	←	5	1	5	4018.37	0.0018
6	0	6	←	5	0	5	4026.708	0.0017
6	1	5	←	5	2	4	4032.87	-0.0086
6	1	6	←	5	0	5	4033.572	0.0005
5	3	3	←	4	2	2	4141.928	-0.0021
6	2	5	←	5	2	4	4146.442	0.0009
5	3	2	←	4	2	2	4170.923	0.0005
6	3	4	←	5	3	3	4205.208	-0.0007
6	4	3	←	5	4	2	4210.516	0.0003
6	1	5	←	5	1	4	4210.962	0.0012
6	4	2	←	5	4	1	4214.72	-0.0014
5	3	3	←	4	2	3	4250.251	0
6	3	3	←	5	3	2	4255.289	-0.001
5	3	2	←	4	2	3	4279.243	-0.0004
6	2	4	←	5	2	3	4298.703	-0.001
5	4	2	←	4	3	1	4492.376	-0.0001
5	4	1	←	4	3	1	4493.469	0.001
5	4	2	←	4	3	2	4500.022	0.0008
5	4	1	←	4	3	2	4501.114	0.0004
7	0	7	←	6	1	6	4674.155	-0.0054
7	1	7	←	6	1	6	4677.112	0.0016
7	0	7	←	6	0	6	4681.027	0.0019
7	1	7	←	6	0	6	4683.977	0.0017

6	2	4	←	5	1	4	4696.404	-0.0021
7	1	6	←	6	2	5	4754.547	-0.001
7	2	6	←	6	2	5	4819.299	0.0009
7	1	6	←	6	1	5	4868.112	0.0017
7	3	5	←	6	3	4	4900.897	0.0001
7	6	1	←	6	6	0	4904.433	-0.0032
7	6	2	←	6	6	1	4904.433	0.0104
7	5	3	←	6	5	2	4910.614	-0.0008
7	5	2	←	6	5	1	4911.262	-0.0013
7	4	4	←	6	4	3	4917.409	0.0003
7	4	3	←	6	4	2	4930.581	-0.0001
7	2	6	←	6	1	5	4932.858	-0.0028
6	3	4	←	5	2	4	4988.069	0.0014
7	3	4	←	6	3	3	4992.509	-0.0012
7	2	5	←	6	2	4	5005.425	-0.0002
6	4	3	←	5	3	2	5176.391	0.0034
6	4	2	←	5	3	2	5181.686	0.001
6	4	3	←	5	3	3	5205.383	0.003
6	4	2	←	5	3	3	5210.678	0.0006
8	0	8	←	7	1	7	5333.52	0.0001
8	1	8	←	7	1	7	5334.746	0.0019
8	0	8	←	7	0	7	5336.471	0.0015
8	1	8	←	7	0	7	5337.692	-0.002
8	1	7	←	7	2	6	5453.004	0.0009
6	5	2	←	5	4	1	5481.755	-0.001
6	5	1	←	5	4	1	5481.89	0.0008
6	5	2	←	5	4	2	5482.846	-0.0016
6	5	1	←	5	4	2	5482.982	0.0013
8	2	7	←	7	2	6	5486.644	0.0013
8	1	7	←	7	1	6	5517.756	0.0029
7	3	4	←	6	2	4	5541.327	0.0012
8	2	7	←	7	1	6	5551.396	0.0028
8	3	6	←	7	3	5	5590.596	0
8	7	1	←	7	7	0	5604.276	-0.0015
8	7	2	←	7	7	1	5604.276	-0.0001
8	6	3	←	7	6	2	5609.934	0.0021
8	6	2	←	7	6	1	5610.017	-0.0013
8	5	4	←	7	5	3	5618.654	0.0001
8	5	3	←	7	5	2	5621.151	-0.0008
8	4	5	←	7	4	4	5623.766	-0.0015
8	4	4	←	7	4	3	5656.4	-0.0015
8	2	6	←	7	2	5	5694.936	0
8	3	5	←	7	3	4	5728.817	-0.0023
7	3	5	←	6	2	5	5742.522	-0.0011

6	6	0	←	5	5	1	5767.889	-0.0065
6	6	1	←	5	5	0	5767.889	0.0069
6	6	1	←	5	5	1	5767.889	-0.0053
6	6	0	←	5	5	0	5767.889	0.0058
7	4	4	←	6	3	3	5838.512	0.0056
7	4	3	←	6	3	3	5856.978	0.0015
7	4	4	←	6	3	4	5917.582	0.0019
7	4	3	←	6	3	4	5936.048	-0.0023
8	3	6	←	7	2	5	5955.808	-0.0019
9	0	9	←	8	1	8	5991.318	0.0007
9	1	9	←	8	1	8	5991.813	0.0012
9	0	9	←	8	0	8	5992.543	0.0015
9	1	9	←	8	0	8	5993.038	0.002
9	1	8	←	8	2	7	6133.372	-0.0007
9	2	8	←	8	2	7	6149.684	0.0012
9	1	8	←	8	1	7	6167.014	0.0011
7	5	3	←	6	4	2	6177.635	-0.0128
7	5	2	←	6	4	2	6178.432	0.0015
7	5	3	←	6	4	3	6182.946	-0.0002
9	2	8	←	8	1	7	6183.321	-0.0019
7	5	2	←	6	4	3	6183.728	0.0002
8	3	5	←	7	2	5	6264.722	0.0018
9	3	7	←	8	3	6	6273.39	0.0001
9	8	1	←	8	8	0	6304.175	0.0005
9	8	2	←	8	8	1	6304.175	0.0007
9	7	2	←	8	7	1	6309.35	-0.004
9	7	3	←	8	7	2	6309.35	0.0064
9	6	4	←	8	6	3	6317.377	0.0002
9	6	3	←	8	6	2	6317.769	-0.0005
9	4	6	←	8	4	5	6327.537	-0.0002
9	5	5	←	8	5	4	6328.371	0.0004
9	5	4	←	8	5	3	6336.029	-0.0014
9	2	7	←	8	2	6	6365.067	-0.0003
9	3	6	←	8	3	5	6454.722	-0.0005
8	4	5	←	7	3	4	6469.759	-0.0043
8	3	6	←	7	2	6	6513.818	-0.0023
8	4	4	←	7	3	4	6520.868	-0.0002
9	3	7	←	8	2	6	6534.268	0.0045
8	4	5	←	7	3	5	6640.451	0.0007
10	0	10	←	9	1	9	6648.41	-0.0024
10	1	10	←	9	1	9	6648.609	0.0009
10	0	10	←	9	0	9	6648.908	0.0009
10	1	10	←	9	0	9	6649.096	-0.0068
8	4	4	←	7	3	5	6691.556	0.0009

7	7	0	←	6	6	1	6752.934	-0.0017
7	7	1	←	6	6	0	6752.934	-0.0005
7	7	1	←	6	6	1	6752.934	-0.0016
7	7	0	←	6	6	0	6752.934	-0.0006
10	2	9	←	9	2	8	6809.771	0.0014
10	1	9	←	9	1	8	6818.56	0.0021
10	2	9	←	9	1	8	6826.08	0.0001
10	1	9	←	9	2	8	6802.25	0.0028
10	2	8	←	9	3	7	6849.572	-0.0035
8	5	3	←	7	4	3	6869.003	0.0018
8	5	4	←	7	4	4	6884.193	0.0022
8	5	3	←	7	4	4	6887.474	0.0026
10	3	8	←	9	3	7	6949.189	0.0003
10	9	1	←	9	9	0	7004.098	-0.002
10	9	2	←	9	9	1	7004.098	-0.002
10	8	2	←	9	8	1	7008.909	0.0012
10	8	3	←	9	8	2	7008.909	0.0024
10	2	8	←	9	2	7	7018.772	0.0018
10	4	7	←	9	4	6	7026.607	0
10	6	5	←	9	6	4	7026.887	0.0012
10	6	4	←	9	6	3	7028.301	0.0003
10	5	6	←	9	5	5	7038.811	0.0003
10	5	5	←	9	5	4	7058.459	0.0002
9	4	6	←	8	3	5	7068.456	-0.0248
10	3	8	←	9	2	7	7118.385	0.0007
10	4	6	←	9	4	5	7139.003	-0.0014
10	3	7	←	9	3	6	7164.212	-0.0004
8	6	3	←	7	5	2	7166.235	-0.0018
8	6	2	←	7	5	2	7166.339	0.0013
8	6	3	←	7	5	3	7167.015	-0.0037
8	6	2	←	7	5	3	7167.119	-0.0007
9	4	5	←	8	3	5	7185.883	0.0037
11	0	11	←	10	1	10	7305.192	-0.0062
11	1	11	←	10	1	10	7305.275	0.0006
11	0	11	←	10	0	10	7305.392	-0.0015
8	7	1	←	7	6	2	7452.785	-0.0051
8	7	2	←	7	6	1	7452.785	0.0111
8	7	2	←	7	6	2	7452.785	-0.0036
8	7	1	←	7	6	1	7452.785	0.0096
11	1	10	←	10	2	9	7464.72	0.0043
11	2	10	←	10	2	9	7468.058	0.0012
11	1	10	←	10	1	9	7472.237	0
11	2	10	←	10	1	9	7475.581	0.0017
9	5	5	←	8	4	4	7537.695	0.0052

9	5	4	←	8	4	4	7548.627	-0.003
11	2	9	←	10	3	8	7564.744	-0.0023
9	5	4	←	8	4	5	7599.74	0.0055
11	3	9	←	10	3	8	7618.697	0.0027
11	2	9	←	10	2	8	7664.363	0.0036
11	9	2	←	10	9	1	7708.574	-0.0015
11	9	3	←	10	9	2	7708.574	-0.0013
11	8	3	←	10	8	2	7715.025	-0.0027
11	8	4	←	10	8	3	7715.025	0.0042
11	4	8	←	10	4	7	7719.271	0.0011
11	7	5	←	10	7	4	7724.592	0.0033
11	7	4	←	10	7	3	7724.81	-0.0029
8	8	0	←	7	7	1	7737.957	-0.0007
8	8	1	←	7	7	0	7737.957	-0.0006
8	8	1	←	7	7	1	7737.957	-0.0007
8	8	0	←	7	7	0	7737.957	-0.0006
11	6	6	←	10	6	5	7738.362	-0.0015
11	6	5	←	10	6	4	7742.647	-0.0007
11	5	7	←	10	5	6	7748.378	-0.0004
11	5	6	←	10	5	5	7791.46	0.0002
11	3	8	←	10	3	7	7853.576	0.0002
9	6	4	←	8	5	3	7862.461	-0.0011
9	6	4	←	8	5	4	7865.744	0.0022
9	6	3	←	8	5	4	7866.234	-0.0019
10	4	6	←	9	3	6	7870.163	0.0024
11	4	7	←	10	4	6	7882.089	-0.0021
9	5	5	←	8	4	5	7588.794	-0.0007

Table S34 Observed rotational transitions (Obs.) and residuals (Obs.-Calc.) for the sevoflurane – 4 water CounterClockwise complex (in MHz).

J'	Ka'	Kc'	←	J''	Ka''	Kc''	υ_{obs}	υ_{obs}-υ_{cal}
4	1	4	←	3	1	3	2600.891	-0.0002
4	0	4	←	3	0	3	2632.05	0
4	1	4	←	3	0	3	2673.205	-0.0046
4	2	3	←	3	2	2	2694.625	0.0008
4	3	2	←	3	3	1	2715.012	-0.0155
4	3	1	←	3	3	0	2720.919	-0.0121
4	2	2	←	3	2	1	2763.569	-0.0007
4	1	3	←	3	1	2	2767.568	0.0002
5	0	5	←	4	1	4	3220.159	0.0025
5	1	5	←	4	1	4	3241.194	-0.0002
5	0	5	←	4	0	4	3261.315	-0.0006
5	1	5	←	4	0	4	3282.359	0.0054
5	2	4	←	4	2	3	3358.071	0
5	4	1	←	4	4	0	3395.455	-0.0107
5	3	3	←	4	3	2	3395.794	-0.0008
5	4	2	←	4	4	1	3394.671	0.0079
5	3	2	←	4	3	1	3415.324	-0.0046
5	1	4	←	4	1	3	3437.378	-0.0009
5	2	3	←	4	2	2	3472.302	-0.0005
4	4	1	←	3	3	0	3871.094	-0.0019
4	4	0	←	3	3	0	3871.199	0.0012
4	4	1	←	3	3	1	3872.105	0.0005
4	4	0	←	3	3	1	3872.205	-0.0015
6	0	6	←	5	1	5	3868.176	0.0025
6	1	6	←	5	1	5	3878.158	-0.0001
6	1	5	←	5	2	4	3864.967	0.0007
6	0	6	←	5	0	5	3889.212	0.0002
6	2	5	←	5	2	4	4015.367	-0.0003
6	3	4	←	5	3	3	4074.647	0.0013
6	4	3	←	5	4	2	4078.618	-0.0002
6	4	2	←	5	4	1	4082.124	-0.001
6	1	5	←	5	1	4	4090.157	-0.0004
6	3	3	←	5	3	2	4121.433	-0.0005
6	2	4	←	5	2	3	4174.224	-0.0108
5	3	3	←	4	2	3	4256.136	-0.0006
7	0	7	←	6	1	6	4508.35	-0.002
7	1	7	←	6	1	6	4512.858	0
7	0	7	←	6	0	6	4518.337	0.0005
7	1	7	←	6	0	6	4522.844	0.0013
5	4	2	←	4	3	1	4544.826	-0.0002

5	4	1	←	4	3	1	4545.732	0.0007
5	4	2	←	4	3	2	4551.736	-0.0037
5	4	1	←	4	3	2	4552.644	-0.0008
7	2	6	←	6	2	5	4666.295	0.0001
7	1	6	←	6	1	5	4726.234	-0.003
7	3	5	←	6	3	4	4749.527	-0.0002
6	3	4	←	5	2	3	4750.084	0.007
7	6	1	←	6	6	0	4750.397	-0.0041
7	6	2	←	6	6	1	4750.397	0.0054
7	5	3	←	6	5	2	4756.505	0.0036
7	5	2	←	6	5	1	4756.995	-0.0017
7	4	4	←	6	4	3	4763.865	-0.0016
7	4	3	←	6	4	2	4774.967	-0.0005
7	2	6	←	6	1	5	4816.706	0.0094
6	3	3	←	5	2	3	4823.306	-0.0057
7	3	4	←	6	3	3	4837.682	0.0009
7	2	5	←	6	2	4	4863.653	-0.0003
5	5	0	←	4	4	0	4880.998	0.0026
5	5	1	←	4	4	0	4880.998	0.0119
5	5	1	←	4	4	1	4881.094	0.0066
5	5	0	←	4	4	1	4881.094	-0.0027
8	2	6	←	7	3	5	5074.793	0.0041
12	3	10	←	11	5	7	5099.732	-0.0035
8	0	8	←	7	1	7	5144.244	0.0016
8	1	8	←	7	1	7	5146.205	-0.0002
8	0	8	←	7	0	7	5148.749	0.0005
8	1	8	←	7	0	7	5150.713	0.0024
6	4	3	←	5	3	2	5208.11	-0.0064
6	4	2	←	5	3	2	5212.526	-0.0017
6	4	2	←	5	3	3	5238.974	-0.001
8	1	7	←	7	2	6	5261.692	0.0012
8	2	7	←	7	2	6	5311.337	-0.0007
8	1	7	←	7	1	6	5352.151	0.0001
8	3	6	←	7	3	5	5418.702	-0.0001
8	7	1	←	7	7	0	5428.255	0.009
8	7	2	←	7	7	1	5428.255	0.01
8	5	4	←	7	5	3	5442.507	0.0009
8	5	3	←	7	5	2	5444.426	0.0007
8	4	5	←	7	4	4	5449.071	0.0015
8	4	4	←	7	4	3	5477.096	0.0001
8	2	6	←	7	2	5	5536.505	0
8	3	5	←	7	3	4	5556.018	-0.0011
9	0	9	←	8	1	8	5777.985	-0.0009
9	1	9	←	8	1	8	5778.82	0.0013

9	0	9	←	8	0	8	5779.95	0.0008
9	1	9	←	8	0	8	5780.783	0.0012
9	2	7	←	8	3	6	5845.921	0.0072
7	4	3	←	6	3	3	5866.063	0.0011
7	3	4	←	6	2	5	5868.266	0.0064
6	6	1	←	5	5	1	5890.379	-0.0041
6	6	0	←	5	5	0	5890.379	0.0044
6	6	0	←	5	5	1	5890.379	-0.0049
6	6	1	←	5	5	0	5890.379	0.0052
7	4	4	←	6	3	4	5923.781	-0.0028
9	1	8	←	8	2	7	5926.165	0.0022
9	2	8	←	8	2	7	5951.554	0.0007
9	1	8	←	8	1	7	5975.81	0.001
9	2	8	←	8	1	7	6001.199	-0.0005
9	3	7	←	8	3	6	6081.046	-0.0009
9	8	1	←	8	8	0	6106.144	0.0057
9	8	2	←	8	8	1	6106.144	0.0058
9	7	2	←	8	7	1	6111.194	-0.0029
9	7	3	←	8	7	2	6111.194	0.0038
9	6	4	←	8	6	3	6119.078	0.0062
9	6	3	←	8	6	2	6119.348	0.0002
9	5	5	←	8	5	4	6130.383	-0.0041
9	4	6	←	8	4	5	6132.324	-0.0017
9	5	4	←	8	5	3	6136.33	-0.0002
9	2	7	←	8	2	6	6189.827	-0.0007
9	4	5	←	8	4	4	6191.003	0
7	5	3	←	6	4	2	6232.486	0.0002
7	5	2	←	6	4	2	6233.078	-0.0035
7	5	3	←	6	4	3	6236.896	-0.001
7	5	2	←	6	4	3	6237.494	0.0005
9	3	6	←	8	3	5	6266.445	-0.0009
10	0	10	←	9	1	9	6410.71	-0.0029
10	1	10	←	9	1	9	6411.06	0.0018
10	0	10	←	9	0	9	6411.546	0.0009
10	1	10	←	9	0	9	6411.893	0.0019
9	3	7	←	8	2	6	6424.961	0.0009
8	2	7	←	7	1	7	6447.895	0.0002
8	4	5	←	7	3	4	6461.932	-0.006
8	4	4	←	7	3	4	6505.48	0.0023
10	1	9	←	9	2	8	6575.926	-0.0019
10	2	9	←	9	2	8	6588.258	0.0005
10	1	9	←	9	1	8	6601.319	0.0008
10	3	8	←	9	3	7	6736.191	-0.0004
10	9	1	←	9	9	0	6784.036	-0.0169

10	9	2	←	9	9	1	6784.036	-0.0168
10	8	2	←	9	8	1	6788.754	0.0042
10	8	3	←	9	8	2	6788.754	0.0049
10	6	5	←	9	6	4	6806.437	0.0047
10	6	4	←	9	6	3	6807.434	0.0001
10	4	7	←	9	4	6	6811.536	0.0006
10	5	6	←	9	5	5	6819.421	-0.0001
10	2	8	←	9	2	7	6824.439	-0.0002
10	5	5	←	9	5	4	6834.911	-0.0013
7	7	1	←	6	6	1	6899.685	-0.0002
7	7	0	←	6	6	0	6899.685	0.0005
7	7	0	←	6	6	1	6899.685	-0.0003
7	7	1	←	6	6	0	6899.685	0.0006
8	5	4	←	7	4	3	6900.025	0.0013
8	5	3	←	7	4	3	6902.531	-0.0084
10	4	6	←	9	4	5	6915.122	-0.0074
10	3	7	←	9	3	6	6961.903	-0.001
11	0	11	←	10	1	10	7042.964	-0.0049
11	1	11	←	10	1	10	7043.111	0.0011
11	0	11	←	10	0	10	7043.313	-0.0011
11	2	10	←	10	2	9	7222.68	0.0005
11	1	10	←	10	1	9	7229.25	0.0012
8	6	3	←	7	5	2	7244.866	-0.0022
8	6	2	←	7	5	2	7244.942	0.0036
8	6	3	←	7	5	3	7245.463	-0.0012
8	6	2	←	7	5	3	7245.537	0.0013
9	4	6	←	8	3	6	7336.947	-0.0041
11	3	9	←	10	3	8	7384.54	-0.0006
11	2	9	←	10	2	8	7446.964	0.0008
11	9	2	←	10	9	1	7466.409	0.0032
11	9	3	←	10	9	2	7466.409	0.0033
11	8	3	←	10	8	2	7472.702	-0.0015
11	8	4	←	10	8	3	7472.702	0.0026
11	7	5	←	10	7	4	7482.055	0.0034
11	7	4	←	10	7	3	7482.19	-0.0065
11	4	8	←	10	4	7	7484.834	0.0036
11	6	6	←	10	6	5	7495.867	-0.0015
11	6	5	←	10	6	4	7498.936	0.0074
11	5	7	←	10	5	6	7508.275	-0.001
11	5	6	←	10	5	5	7543.121	-0.0002
9	5	5	←	8	4	5	7596.857	0.0028
9	5	4	←	8	4	5	7605.316	0.0032
11	3	8	←	10	3	7	7638.069	-0.0036
11	4	7	←	10	4	6	7641.605	-0.0033

12	0	12	←	11	0	11	7675.146	0.002
12	1	12	←	11	1	11	7675.054	-0.0055
12	2	11	←	11	2	10	7855.763	0.0027
12	1	11	←	11	1	10	7858.911	0.0028
8	8	1	←	7	7	1	7908.962	-0.0007
8	8	0	←	7	7	0	7908.962	-0.0006
8	8	0	←	7	7	1	7908.962	-0.0007
8	8	1	←	7	7	0	7908.962	-0.0006

3.2 Least Squares Fit r_0 Structural Analysis

Derivation of reliable experimental structures for the studied clusters presents considerable challenges. Even though multiple heavy nuclei have been substituted isotopically, such substitution is not possible for the seven fluorine nuclei. Furthermore, Kraitchman substitution analysis performed above revealed that for each cluster there is one imaginary coordinate for a carbon atom in the sevoflurane backbone. This is illustrative of non negligible vibrational contributions to the determined ground state rotational constants.

For these reasons an independent determination of the geometry of the sevoflurane unit in each cluster was not realistic. At the same time, availability of rotational constants for several isotopic species could be used to perform a maximally robust least-squares r_0 analysis in order to determine the most plausible values for geometrical parameters describing the orientation of water units in relation to sevoflurane.

The adopted procedure was to use the quantum chemistry computed structure as the basis for the assumptions. This was first scaled to reproduce the three ground state rotational constants of the parent species, by using the planar moment based scaling in program CORSCL from the PROSPE website. The next step involved search for a minimal set of parameters of fit that would meet the criteria of a small deviation of fit to all observed rotational constants while, at the same time, avoided departure of parameters to nonphysical values. The final fitting models were tested for both B3LYP D3BJ/aug-cc-pVTZ and for RI-MP2/cc-pVTZ starting structures as summarised below (identified by DFT, black, and MP2, red). Only heavy atom distances could be reliably determined and, in each case, the fitted value is above the computed value listed in square brackets.

Abbreviated results of fits are given further below for the DFT versions for the three clusters. Intercomparison of various fitted and computed values shows that uncertainty in derived distance values is at the level of several 0.01Å, even though their nominal statistical uncertainty is often considerably smaller.

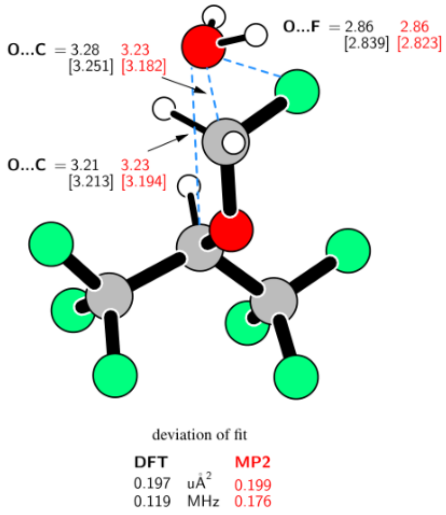

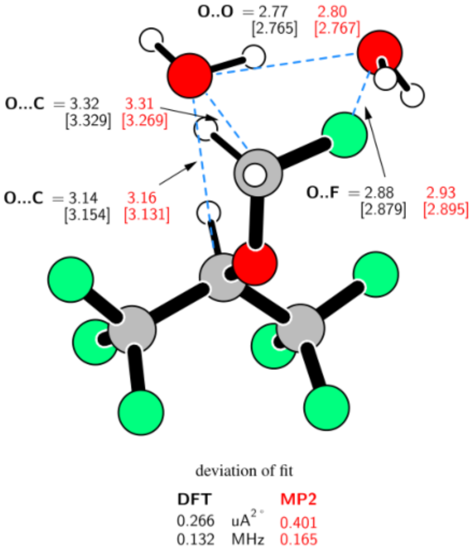

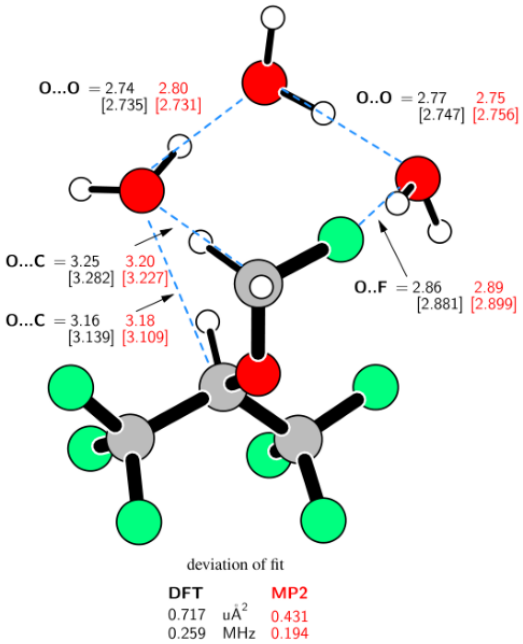

The SEV-(H_2O) cluster

Atom numbering used in the least-squares fit of the Sevoflurane-(H_2O) structure, red and grey numbers in bold are as used in spectroscopic assignment of the isotopic species.

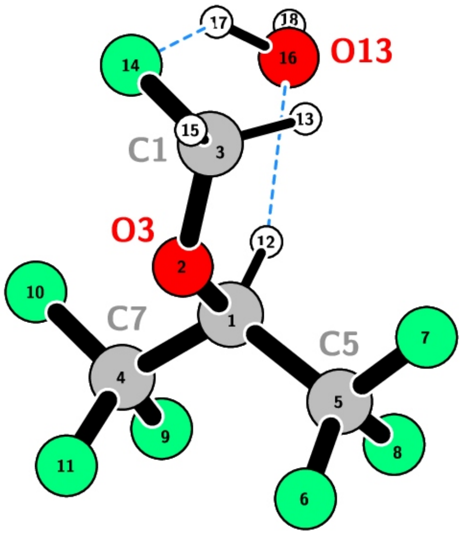

Table S35: The abbreviated results of fitting the partial r_0 geometry of the Sevoflurane-(H_2O) cluster with the STRFIT program.

```
 ___________________________________________________________________________
|                                                                           |
|    STRFIT - General structure fitting program using CART definitions      |
|___________________________________________________________________________|
 version 29.XI.2023                                          Zbigniew KISIEL

_____________________________________________________________________________

Sevoflurane...H2O
_____________________________________________________________________________

!
!    starting structure = planar moment scaled ORCA
!
!                                unscaled ORCA
!
!    CF3                         Acalc = 823.02   Aobs   822.23653
!       \                        Bcalc = 642.90   Bobs   645.85854
!      H-C-O-CF3 + H2O           Ccalc = 528.06   Cobs   533.76059
!       /
!    CF3
!
!

NUMBER OF ATOMS = 18

  NO  NA  NB  NC       NO.NA      NO.NA.NB    NO.NA.NB.NC        MASS

   1   0   0   0     0.000000     0.000000     0.000000     12.0000000
   2   1   0   0     1.419009     0.000000     0.000000     15.9949150
   3   2   1   0     1.373203   116.204203     0.000000     12.0000000
   4   1   2   3     1.529196   107.403236  -129.720887     12.0000000
   5   1   2   3     1.528196   108.346048   106.281924     12.0000000
   6   5   1   2     1.339972   112.701802    60.867522     18.9984046
   7   5   1   2     1.344029   108.808486   -58.140483     18.9984046
   8   5   1   2     1.347272   111.213266  -176.310113     18.9984046
   9   4   1   2     1.348418   110.733048  -178.096852     18.9984046
  10   4   1   2     1.334696   109.432548    63.510537     18.9984046
  11   4   1   2     1.342624   112.440469   -56.048896     18.9984046
  12   1   2   3     1.095813   113.082374   -12.344174      1.0078250
  13   3   2   1     1.091572   112.626415   -39.143836      1.0078250
  14   3   2   1     1.394585   110.333440    79.267437     18.9984046
  15   3   2   1     1.092453   106.733694  -164.315045      1.0078250
  16   1   2   3     3.211629    99.736995   -18.202156     15.9949150
  17  16   1   2     0.965715    88.055439   -33.972258      1.0078250
  18  16  17   1     0.966104   106.898153   141.066989      1.0078250
```

```
--------------------------------------------
TOTAL NUMBER OF STRUCTURAL PARAMETERS:    3
--------------------------------------------

Parameters to be fitted:
          R(16, 1) =     3.500000
       A(16, 1, 2) =    99.736995
    D(16, 1, 2, 3) =   -18.202156

--------------------------------------------------------------------------------------
TOTAL NUMBER OF SPECTROSCOPIC CONSTANTS:    18
```

Isotopic species		B_expt	Ib_expt	dI (or g_bb)	dB	dB_el	B_corr	Ib_corr
	A	822.23653	614.63945	0.00000	0.00000	0.00000	822.23653	614.63945
	B	645.85854	782.49180	0.00000	0.00000	0.00000	645.85854	782.49180
	C	533.76059	946.82713	0.00000	0.00000	0.00000	533.76059	946.82713
2	A	819.11534	616.98150	0.00000	0.00000	0.00000	819.11534	616.98150
	B	642.03232	787.15509	0.00000	0.00000	0.00000	642.03232	787.15509
	C	531.26993	951.26598	0.00000	0.00000	0.00000	531.26993	951.26598
3	A	820.95002	615.60265	0.00000	0.00000	0.00000	820.95002	615.60265
	B	644.58947	784.03237	0.00000	0.00000	0.00000	644.58947	784.03237
	C	532.35177	949.33283	0.00000	0.00000	0.00000	532.35177	949.33283
4	A	819.63397	616.59110	0.00000	0.00000	0.00000	819.63397	616.59110
	B	645.77566	782.59222	0.00000	0.00000	0.00000	645.77566	782.59222
	C	532.59909	948.89199	0.00000	0.00000	0.00000	532.59909	948.89199
5	A	817.88819	617.90721	0.00000	0.00000	0.00000	817.88819	617.90721
	B	642.76207	786.26141	0.00000	0.00000	0.00000	642.76207	786.26141
	C	533.19846	947.82534	0.00000	0.00000	0.00000	533.19846	947.82534
6	A	810.49226	623.54576	0.00000	0.00000	0.00000	810.49226	623.54576
	B	629.62210	802.67038	0.00000	0.00000	0.00000	629.62210	802.67038
	C	526.60688	959.68934	0.00000	0.00000	0.00000	526.60688	959.68934

```
    B_corr = B_expt + dB - dB_el, Ib_corr=505379.01/B_corr
    g_bb is dimensionles, dB_el= = 0.000544617 * g_bb * B, where B=B_expt+dB
or
    Ib_corr = Ib_expt + dI,        B_corr =505379.01/Ib_corr


-------------------------------------------------------
DEFINITIONS OF SUBSTITUTED ISOTOPIC SPECIES
-------------------------------------------------------
!
!   species 2  = C1
!
ISOTOPIC SPECIES  2,  changes from parent species:
atom no.,parameter no.,value     3  4       13.0033544
!
!   species 3  = C5
!
ISOTOPIC SPECIES  3,  changes from parent species:
atom no.,parameter no.,value     5  4       13.0033544
!
!   species 4  = C7
!
ISOTOPIC SPECIES  4,  changes from parent species:
atom no.,parameter no.,value     4  4       13.0033544
!
!   species 5  = O3
!
ISOTOPIC SPECIES  5,  changes from parent species:
atom no.,parameter no.,value     2  4       17.9991610
!
!   species 6  = O13
!
ISOTOPIC SPECIES  6,  changes from parent species:
atom no.,parameter no.,value    16  4       17.9991610

______________________________________________________________________________
fit after:   4 iterations,  ALAMDA=  0.10E-06

   Number of fitted spectroscopic constants = 18
                Number of parameters of fit =  3
               Number of degrees of freedom = 15

FINAL RESULTS OF LEAST SQUARES FIT:                         starting value
```

```
          R(16, 1) =     3.212203 +-  0.000559         O.C1    3.2116
       A(16, 1, 2) =    99.726539 +-  0.023841       H.O.C1   99.74
    D(16, 1, 2, 3) =   -18.200355 +-  0.066158   O.C1.O2.C3  -18.20

     Chi-squared =        0.5803275595     = Sum( (Iobs-calc)**2 )
 Deviation of fit =        0.196694 uA^2    = Sqrt(Chisq/Ndegf),  Ndegf= 15

 Note that the fit is to moments of inertia but it also corresponds to:
 Deviation of fit =        0.118875 MHz     = Sqrt( Sum( (Bo-c)**2 )/Ndegf )
```

Ni	Axis	Iobs	Icalc	Io-c	Bobs	Bcalc	Bo-c
1	a	614.63945	614.64037	-0.00092	822.23653	822.23530	0.00123
1	b	782.49180	782.55360	-0.06180	645.85854	645.80753	0.05101
1	c	946.82713	946.89983	-0.07269	533.76059	533.71961	0.04098
2	a	616.98150	617.00533	-0.02383	819.11534	819.08370	0.03164
2	b	787.15509	787.07687	0.07822	642.03232	642.09613	-0.06381
2	c	951.26598	951.45849	-0.19251	531.26993	531.16244	0.10749
3	a	615.60265	615.61705	-0.01440	820.95002	820.93081	0.01921
3	b	784.03237	784.14187	-0.10950	644.58947	644.49946	0.09001
3	c	949.33283	949.44340	-0.11057	532.35177	532.28977	0.06200
4	a	616.59110	616.61478	-0.02368	819.63397	819.60250	0.03147
4	b	782.59222	782.66243	-0.07021	645.77566	645.71773	0.05793
4	c	948.89199	948.98304	-0.09105	532.59909	532.54799	0.05110
5	a	617.90721	617.82907	0.07814	817.88819	817.99163	-0.10344
5	b	786.26141	786.29390	-0.03249	642.76207	642.73551	0.02656
5	c	947.82534	948.07040	-0.24506	533.19846	533.06064	0.13782
6	a	623.54576	623.59526	-0.04950	810.49226	810.42792	0.06434
6	b	802.67038	802.48548	0.18490	629.62210	629.76717	-0.14507
6	c	959.68934	959.06852	0.62082	526.60688	526.94776	-0.34088

Correlation coefficients:

		1	2	3
1:	R(16, 1)	1.000		
2:	A(16, 1, 2)	-0.045	1.000	
3:	D(16, 1, 2, 3)	0.141	-0.663	1.000

Principal coordinates and estimated uncertainties:

ATOM NO.	A	dA	B	dB	C	dC	MASS
1	-0.01972	0.00013	0.09129	0.00029	-0.00475	0.00009	12.000000
2	0.65500	0.00051	0.39229	0.00199	1.20675	0.00030	15.994915
3	1.82399	0.00118	1.10318	0.00303	1.08932	0.00084	12.000000
4	-0.32813	0.00135	-1.40646	0.00001	0.00342	0.00121	12.000000
5	-1.25316	0.00098	0.98804	0.00100	-0.10400	0.00092	12.000000
6	-2.11576	0.00087	0.79431	0.00098	0.90293	0.00082	18.998405
7	-0.86019	0.00224	2.27241	0.00057	-0.05512	0.00200	18.998405
8	-1.89520	0.00073	0.80616	0.00263	-1.27441	0.00082	18.998405
9	-1.00954	0.00178	-1.75957	0.00162	-1.10529	0.00147	18.998405
10	0.81510	0.00203	-2.09521	0.00112	0.00974	0.00189	18.998405
11	-1.03327	0.00165	-1.77864	0.00025	1.08365	0.00149	18.998405
12	0.59043	0.00025	0.28122	0.00013	-0.89495	0.00022	1.007825
13	1.76857	0.00188	1.87899	0.00232	0.32344	0.00150	1.007825
14	2.86838	0.00034	0.25954	0.00373	0.71199	0.00035	18.998405
15	2.05850	0.00164	1.48945	0.00412	2.08394	0.00116	1.007825
16	2.37918	0.00079	0.55172	0.00291	-2.09079	0.00085	15.994915
17	2.98564	0.00061	0.22110	0.00194	-1.41589	0.00076	1.007825
18	2.68447	0.00097	0.20223	0.00418	-2.93815	0.00068	1.007825
		|		|		|	

```
 NOTES: 1/ only the uncertainties for those coordinates which are completely
           defined by the fitted internals should be trusted
        2/ the uncertainties are somewhat limited by the linear approximation
           coord=(d coord/d parameter)*parameter used for evaluation
        3/ only the effect of the internals R, A, and D is propagated
```

The SEV-$(H_2O)_2$ cluster

Atom numbering used in the least-squares fit of the Sevoflurane-$(H_2O)_2$ structure, red and grey numbers in bold are as used in spectroscopic assignment of the isotopic species.

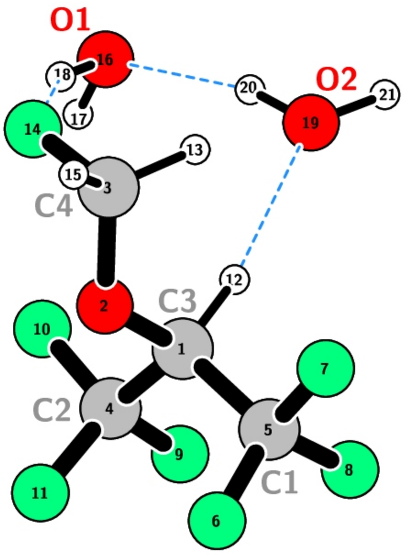

Table S36: The abbreviated results of fitting the partial r_0 geometry of the Sevoflurane-$(H_2O)_2$ cluster with the STRFIT program.

```
 _____________________________________________________________________________
|                                                                             |
|    STRFIT - General structure fitting program using CART definitions        |
|_____________________________________________________________________________|
 version 29.XI.2023                                           Zbigniew KISIEL

_______________________________________________________________________________

Sevoflurane...(H2O)2
_______________________________________________________________________________

!
!    starting structure = planar moment scaled ORCA
!
!                                ORCA computed       obs
!                                (unscaled)
!    CF3                         Acalc = 747.28      737.85016
!       \                        Bcalc = 476.88      481.99047
!      H-C-O-CF3 + (H2O)2        Ccalc = 424.75      425.93344
!       /
!    CF3
!

NUMBER OF ATOMS = 21

  NO  NA  NB  NC       NO.NA      NO.NA.NB   NO.NA.NB.NC       MASS

   1   0   0   0    0.000000     0.000000     0.000000     12.0000000
   2   1   0   0    1.413434     0.000000     0.000000     15.9949150
   3   2   1   0    1.379032   117.291988     0.000000     12.0000000
   4   1   2   3    1.550331   108.561110  -121.696633     12.0000000
   5   1   2   3    1.529286   107.481825   114.073593     12.0000000
   6   5   1   2    1.335929   112.442379    61.355328     18.9984046
   7   5   1   2    1.360647   109.103023   -58.295181     18.9984046
   8   5   1   2    1.342085   111.082970  -176.967562     18.9984046
   9   4   1   2    1.344021   111.396135  -178.342030     18.9984046
  10   4   1   2    1.341640   109.530687    63.349106     18.9984046
  11   4   1   2    1.336818   113.262179   -56.031761     18.9984046
  12   1   2   3    1.092401   112.542979    -2.547960      1.0078250
  13   3   2   1    1.092639   113.155469   -30.812815      1.0078250
  14   3   2   1    1.404245   109.183189    87.948411     18.9984046
  15   3   2   1    1.089189   107.774044  -156.672864      1.0078250
  16  14   3   2    2.879751   122.012624   -84.778065     15.9949150
  17  16  14   3    0.971057   111.083180    99.571163      1.0078250
```

```
  18  16  17   1     0.967962   105.303991   -56.903149      1.0078250
  19   1   2   3     3.150478   103.375564    -2.336132     15.9949150
  20  16   1   2     1.827478    56.206876   115.601107      1.0078250
  21  16  17   1     3.352675   114.834794    57.781023      1.0078250


---------------------------------------------
TOTAL NUMBER OF STRUCTURAL PARAMETERS:     2
---------------------------------------------

Parameters to be fitted:
          R(16,14) =     2.879751
          R(19, 1) =     3.150478

-------------------------------------------------------------------------------------
TOTAL NUMBER OF SPECTROSCOPIC CONSTANTS:    21

Isotopic    B_expt     Ib_expt      dI         dB      dB_el      B_corr     Ib_corr
species                          (or g_bb)
-------------------------------------------------------------------------------------
     A     737.85016  684.93447   0.00000    0.00000  0.00000    737.85016  684.93447
     B     481.99047 1048.52490   0.00000    0.00000  0.00000    481.99047 1048.52490
     C     425.93344 1186.52109   0.00000    0.00000  0.00000    425.93344 1186.52109
 2   A     737.16780  685.56848   0.00000    0.00000  0.00000    737.16780  685.56848
     B     480.67889 1051.38591   0.00000    0.00000  0.00000    480.67889 1051.38591
     C     424.74329 1189.84578   0.00000    0.00000  0.00000    424.74329 1189.84578
 3   A     735.69180  686.94392   0.00000    0.00000  0.00000    735.69180  686.94392
     B     481.96070 1048.58967   0.00000    0.00000  0.00000    481.96070 1048.58967
     C     425.19668 1188.57704   0.00000    0.00000  0.00000    425.19668 1188.57704
 4   A     737.84000  684.94390   0.00000    0.00000  0.00000    737.84000  684.94390
     B     481.97180 1048.56552   0.00000    0.00000  0.00000    481.97180 1048.56552
     C     425.92665 1186.54001   0.00000    0.00000  0.00000    425.92665 1186.54001
 5   A     733.55200  688.94776   0.00000    0.00000  0.00000    733.55200  688.94776
     B     480.34767 1052.11088   0.00000    0.00000  0.00000    480.34767 1052.11088
     C     424.85318 1189.53802   0.00000    0.00000  0.00000    424.85318 1189.53802
 6   A     735.88112  686.76719   0.00000    0.00000  0.00000    735.88112  686.76719
     B     469.05838 1077.43307   0.00000    0.00000  0.00000    469.05838 1077.43307
     C     416.34494 1213.84689   0.00000    0.00000  0.00000    416.34494 1213.84689
 7   A     726.30016  695.82665   0.00000    0.00000  0.00000    726.30016  695.82665
     B     476.16626 1061.34989   0.00000    0.00000  0.00000    476.16626 1061.34989
     C     421.46746 1199.09378   0.00000    0.00000  0.00000    421.46746 1199.09378
excluded

    NO OF CONSTANTS TO FIT TO:    20
    NO OF EXCLUDED CONSTANTS:      1
-------------------------------------------------------------------------------------
    B_corr = B_expt + dB - dB_el, Ib_corr=505379.01/B_corr
    g_bb is dimensionles, dB_el= = 0.000544617 * g_bb * B, where B=B_expt+dB
or
    Ib_corr = Ib_expt + dI,        B_corr =505379.01/Ib_corr


-------------------------------------------------------
DEFINITIONS OF SUBSTITUTED ISOTOPIC SPECIES
-------------------------------------------------------
!
!   species 2  = C5
!
ISOTOPIC SPECIES  2,  changes from parent species:
atom no.,parameter no.,value     5  4       13.0033544
!
!   species 3  = C4
!
ISOTOPIC SPECIES  3,  changes from parent species:
atom no.,parameter no.,value     4  4       13.0033544
!
!   species 4  = C1
!
ISOTOPIC SPECIES  4,  changes from parent species:
atom no.,parameter no.,value     1  4       13.0033544
!
!   species 5  = C3
!
ISOTOPIC SPECIES  5,  changes from parent species:
atom no.,parameter no.,value     3  4       13.0033544
!
!   species 6  = O16
!
```

```
 ISOTOPIC SPECIES  6,  changes from parent species:
 atom no.,parameter no.,value    16  4      17.9991610
 !
 !   species 7  = O19
 !
 ISOTOPIC SPECIES  7,  changes from parent species:
 atom no.,parameter no.,value    19  4      17.9991610

 ______________________________________________________________________________
 fit after:   4 iterations,  ALAMDA=  0.10E-06

    Number of fitted spectroscopic constants = 20
                 Number of parameters of fit =  2
                Number of degrees of freedom = 18

FINAL RESULTS OF LEAST SQUARES FIT:                         starting value

           R(16,14) =     2.883326 +-  0.003216       O.F       2.8798
           R(19, 1) =     3.147028 +-  0.003471       O.C       3.1505

      Chi-squared =        1.2786726337     = Sum( (Iobs-calc)**2 )
 Deviation of fit =        0.266528 uA^2    = Sqrt(Chisq/Ndegf),  Ndegf= 18

 Note that the fit is to moments of inertia but it also corresponds to:
 Deviation of fit =        0.131976 MHz     = Sqrt( Sum( (Bo-c)**2 )/Ndegf )

Ni Axis     Iobs       Icalc      Io-c             Bobs        Bcalc       Bo-c

 1  a    684.93447   684.85907   0.07540        737.85016    737.93139   -0.08123
 1  b   1048.52490  1048.59894  -0.07404        481.99047    481.95644    0.03403
 1  c   1186.52109  1186.55591  -0.03482        425.93344    425.92094    0.01250
 2  a    685.56848   685.39354   0.17494        737.16780    737.35596   -0.18816
 2  b   1051.38591  1051.57721  -0.19130        480.67889    480.59144    0.08745
 2  c   1189.84578  1189.99150  -0.14572        424.74329    424.69128    0.05201
 3  a    686.94392   686.86351   0.08041        735.69180    735.77793   -0.08613
 3  b   1048.58967  1048.66279  -0.07313        481.96070    481.92709    0.03361
 3  c   1188.57704  1188.62286  -0.04583        425.19668    425.18029    0.01639
 4  a    684.94390   684.87984   0.06407        737.84000    737.90902   -0.06902
 4  b   1048.56552  1048.70470  -0.13919        481.97180    481.90783    0.06397
 4  c   1186.54001  1186.67579  -0.13579        425.92665    425.87791    0.04874
 5  a    688.94776   688.82199   0.12578        733.55200    733.68595   -0.13395
 5  b   1052.11088  1051.86859   0.24229        480.34767    480.45831   -0.11064
 5  c   1189.53802  1190.01862  -0.48060        424.85318    424.68160    0.17158
 6  a    686.76719   686.65919   0.10800        735.88112    735.99687   -0.11575
 6  b   1077.43307  1076.81509   0.61798        469.05838    469.32757   -0.26919
 6  c   1213.84689  1213.36741   0.47948        416.34494    416.50947   -0.16453
 7  a    695.82665   696.00557  -0.17892        726.30016    726.11346    0.18670
 7  b   1061.34989  1061.75120  -0.40130        476.16626    475.98629    0.17997

 Deviations for constants excluded from the fit:

Ni Axis     Iobs       Icalc      Io-c             Bobs        Bcalc       Bo-c

 7  c   1199.09378  1197.29852   1.79526        421.46746    422.09942   -0.63196

 Correlation coefficients:

                          1      2

 1:          R(16,14)   1.000
 2:          R(19, 1)  -0.964  1.000
 ______________________________________________________________________________

 Principal coordinates and estimated uncertainties:

 ATOM NO.      A        dA            B       dB           C        dC           MASS

   1      -0.32020  0.00006      0.13215  0.00009      0.05765  0.00010       12.000000
   2       0.09483  0.00047      0.57511  0.00148      1.33411  0.00046       15.994915
   3       1.17169  0.00089      1.43500  0.00206      1.38575  0.00111       12.000000
   4      -0.25079  0.00073     -1.41630  0.00009      0.02597  0.00135       12.000000
   5      -1.71408  0.00028      0.70726  0.00084     -0.19741  0.00085       12.000000
   6      -2.61184  0.00023      0.28411  0.00045      0.69684  0.00061       18.998405
   7      -1.65649  0.00096      2.06457  0.00074     -0.12162  0.00210       18.998405
   8      -2.15289  0.00011      0.38360  0.00219     -1.42374  0.00062       18.998405
   9      -0.66929  0.00115     -1.90180  0.00121     -1.15536  0.00174       18.998405
  10       1.02114  0.00090     -1.81165  0.00087      0.18688  0.00191       18.998405
  11      -0.96655  0.00088     -1.99680  0.00064      0.99436  0.00179       18.998405
```

12	0.31763	0.00011	0.51012	0.00035	-0.74463	0.00037	1.007825
13	1.25047	0.00107	2.07823	0.00128	0.50603	0.00169	1.007825
14	2.35782	0.00055	0.68588	0.00270	1.44761	0.00037	18.998405
15	1.10746	0.00132	1.99261	0.00290	2.31918	0.00163	1.007825
16	3.65259	0.00178	-0.32486	0.00011	-0.92210	0.00170	15.994915
17	3.43898	0.00226	-1.26139	0.00024	-1.06439	0.00086	1.007825
18	3.39075	0.00196	-0.13897	0.00065	-0.00896	0.00177	1.007825
19	1.76805	0.00272	1.45833	0.00216	-1.88766	0.00111	15.994915
20	2.53551	0.00117	0.86966	0.00104	-1.73753	0.00238	1.007825
21	1.86809	0.00028	1.82395	0.00251	-2.77645	0.00290	1.007825
		|		|		|	

```
NOTES: 1/ only the uncertainties for those coordinates which are completely
          defined by the fitted internals should be trusted
       2/ the uncertainties are somewhat limited by the linear approximation
          coord=(d coord/d parameter)*parameter used for evaluation
       3/ only the effect of the internals R, A, and D is propagated
```

The SEV-(H_2O)$_3$ cluster

Atom numbering used in the least-squares fit of the Sevoflurane-(H_2O)$_3$ structure, red and grey numbers in bold are as used in spectroscopic assignment of the isotopic species.

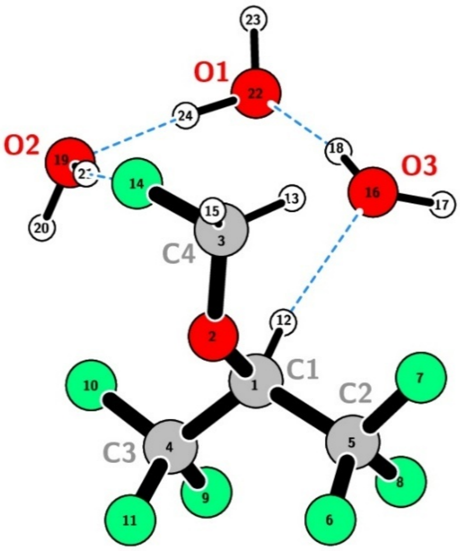

Table S37: The abbreviated results of fitting the partial r_0 geometry of the Sevoflurane-(H_2O)$_3$ cluster with the STRFIT program.

```
 ____________________________________________________________________________
|                                                                            |
|   STRFIT - General structure fitting program using CART definitions        |
|____________________________________________________________________________|
 version 29.XI.2023                                          Zbigniew KISIEL

 ______________________________________________________________________________

Sevoflurane...(H2O)3
 ______________________________________________________________________________

!
!    starting structure = ORCA scaled to obs
!
!                            ORCA computed        obs
!                            (unscaled)
!    CF3                     Acalc = 616.70     Aobs = 612.47396
!       \                    Bcalc = 369.00     Bobs = 372.429002
!      H-C-O-CF3 + (H2O)2    Ccalc = 332.21     Cobs = 328.72967
!       /
!    CF3
!

NUMBER OF ATOMS = 24
```

NO	NA	NB	NC	NO.NA	NO.NA.NB	NO.NA.NB.NC	MASS
1	0	0	0	0.000000	0.000000	0.000000	12.0000000
2	1	0	0	1.388256	0.000000	0.000000	15.9949150
3	2	1	0	1.383183	116.209079	0.000000	12.0000000
4	1	2	3	1.553198	107.376662	-125.593235	12.0000000
5	1	2	3	1.543654	108.083532	110.069747	12.0000000
6	5	1	2	1.339101	112.565106	62.488566	18.9984046
7	5	1	2	1.361576	109.724679	-58.505400	18.9984046
8	5	1	2	1.320426	111.302179	-176.290465	18.9984046
9	4	1	2	1.318843	110.505012	-179.453822	18.9984046
10	4	1	2	1.354736	110.034273	62.263093	18.9984046
11	4	1	2	1.339522	114.129739	-57.878487	18.9984046
12	1	2	3	1.094086	111.758015	-5.958975	1.0078250
13	3	2	1	1.096551	113.961031	-33.295262	1.0078250
14	3	2	1	1.417459	109.243907	86.397055	18.9984046

15	3	2	1	1.068159	106.887728	-158.703098	1.0078250
16	1	2	3	3.161515	102.117353	8.373582	15.9949150
17	16	1	2	0.962576	116.783274	134.118202	1.0078250
18	16	17	1	0.981404	106.803675	-136.800235	1.0078250
19	14	3	2	2.860843	136.611976	-68.667024	15.9949150
20	19	14	3	0.967686	109.430312	74.920660	1.0078250
21	19	20	14	0.959336	104.963326	-4.106369	1.0078250
22	19	14	3	2.770025	97.151597	-49.724154	15.9949150
23	22	14	3	0.961845	111.199299	-116.602255	1.0078250
24	22	23	14	0.991529	106.894381	51.435322	1.0078250

TOTAL NUMBER OF STRUCTURAL PARAMETERS: 3

Parameters to be fitted:
R(16, 1) = 3.500000
R(19,14) = 3.000000
R(22,19) = 3.000000

TOTAL NUMBER OF SPECTROSCOPIC CONSTANTS: 24

Isotopic species		B_expt	Ib_expt	dI (or g_bb)	dB	dB_el	B_corr	Ib_corr
	A	612.47396	825.14367	0.00000	0.00000	0.00000	612.47396	825.14367
	B	372.42900	1356.98081	0.00000	0.00000	0.00000	372.42900	1356.98081
	C	328.72967	1537.36963	0.00000	0.00000	0.00000	328.72967	1537.36963
2	A	612.50470	825.10226	0.00000	0.00000	0.00000	612.50470	825.10226
	B	372.34499	1357.28699	0.00000	0.00000	0.00000	372.34499	1357.28699
	C	328.67462	1537.62712	0.00000	0.00000	0.00000	328.67462	1537.62712
3	A	611.83050	826.01147	0.00000	0.00000	0.00000	611.83050	826.01147
	B	371.56946	1360.11988	0.00000	0.00000	0.00000	371.56946	1360.11988
	C	327.87766	1541.36457	0.00000	0.00000	0.00000	327.87766	1541.36457
4	A	610.93560	827.22141	0.00000	0.00000	0.00000	610.93560	827.22141
	B	372.23946	1357.67178	0.00000	0.00000	0.00000	372.23946	1357.67178
	C	328.21163	1539.79617	0.00000	0.00000	0.00000	328.21163	1539.79617
5	A	609.03680	829.80045	0.00000	0.00000	0.00000	609.03680	829.80045
	B	371.09775	1361.84876	0.00000	0.00000	0.00000	371.09775	1361.84876
	C	328.56690	1538.13123	0.00000	0.00000	0.00000	328.56690	1538.13123
6	A	608.32094	830.77694	0.00000	0.00000	0.00000	608.32094	830.77694
	B	362.64433	1393.59414	0.00000	0.00000	0.00000	362.64433	1393.59414
	C	321.23732	1573.22635	0.00000	0.00000	0.00000	321.23732	1573.22635
7	A	609.87781	828.65617	0.00000	0.00000	0.00000	609.87781	828.65617
	B	366.00231	1380.80826	0.00000	0.00000	0.00000	366.00231	1380.80826
	C	323.04232	1564.43592	0.00000	0.00000	0.00000	323.04232	1564.43592
8	A	605.02078	835.30852	0.00000	0.00000	0.00000	605.02078	835.30852
	B	370.84356	1362.78221	0.00000	0.00000	0.00000	370.84356	1362.78221
	C	325.61200	1552.08963	0.00000	0.00000	0.00000	325.61200	1552.08963

B_corr = B_expt + dB - dB_el, Ib_corr=505379.01/B_corr
g_bb is dimensionles, dB_el= = 0.000544617 * g_bb * B, where B=B_expt+dB
or
Ib_corr = Ib_expt + dI, B_corr =505379.01/Ib_corr

DEFINITIONS OF SUBSTITUTED ISOTOPIC SPECIES

!
!
! species 2 = C1
!
ISOTOPIC SPECIES 2, changes from parent species:
atom no.,parameter no.,value 1 4 13.0033544
!

```
!   species 3  = C2
!
ISOTOPIC SPECIES  3,  changes from parent species:
atom no.,parameter no.,value     5  4       13.0033544
!
!   species 4  = C3
!
ISOTOPIC SPECIES  4,  changes from parent species:
atom no.,parameter no.,value     4  4       13.0033544
!
!   species 5  = C4
!
ISOTOPIC SPECIES  5,  changes from parent species:
atom no.,parameter no.,value     3  4       13.0033544
!
!   species 6  = O22
!
ISOTOPIC SPECIES  6,  changes from parent species:
atom no.,parameter no.,value    22  4       17.9991610
!
!   species 7  = O19
!
ISOTOPIC SPECIES  7,  changes from parent species:
atom no.,parameter no.,value    19  4       17.9991610
!
!   species 8  = O16
!
ISOTOPIC SPECIES  8,  changes from parent species:
atom no.,parameter no.,value    16  4       17.9991610

_______________________________________________________________________________
fit after:   5 iterations,  ALAMDA=  0.10E-07

   Number of fitted spectroscopic constants = 24
                Number of parameters of fit =  3
               Number of degrees of freedom = 21

FINAL RESULTS OF LEAST SQUARES FIT:

         R(16, 1) =     3.159525 +-  0.008330        O.C      3.1595
         R(19,14) =     2.862043 +-  0.001867        O.F      2.8620
         R(22,19) =     2.771328 +-  0.010996        O.O      2.7713

     Chi-squared =       10.8204335405     = Sum( (Iobs-calc)**2 )
Deviation of fit =        0.717815 uA^2    = Sqrt(Chisq/Ndegf),  Ndegf= 21

Note that the fit is to moments of inertia but it also corresponds to:
Deviation of fit =        0.259079 MHz     = Sqrt( Sum( (Bo-c)**2 )/Ndegf )

Ni Axis     Iobs       Icalc      Io-c            Bobs        Bcalc       Bo-c

 1  a    825.14367   825.14261   0.00105        612.47396    612.47474   -0.00078
 1  b   1356.98081  1357.18825  -0.20744        372.42900    372.37208    0.05692
 1  c   1537.36963  1537.44585  -0.07623        328.72967    328.71337    0.01630
 2  a    825.10226   825.16294  -0.06069        612.50470    612.45965    0.04505
 2  b   1357.28699  1357.54707  -0.26009        372.34499    372.27365    0.07134
 2  c   1537.62712  1537.78752  -0.16040        328.67462    328.64034    0.03428
 3  a    826.01147   825.93273   0.07874        611.83050    611.88883   -0.05833
 3  b   1360.11988  1360.62555  -0.50567        371.56946    371.43137    0.13809
 3  c   1541.36457  1541.63709  -0.27251        327.87766    327.81970    0.05796
 4  a    827.22141   827.30297  -0.08156        610.93560    610.87537    0.06023
 4  b   1357.67178  1357.76403  -0.09225        372.23946    372.21417    0.02529
 4  c   1539.79617  1539.84256  -0.04639        328.21163    328.20174    0.00989
 5  a    829.80045   829.70365   0.09680        609.03680    609.10786   -0.07106
 5  b   1361.84876  1361.76318   0.08558        371.09775    371.12107   -0.02332
 5  c   1538.13123  1538.32252  -0.19129        328.56690    328.52604    0.04086
 6  a    830.77694   831.66437  -0.88743        608.32094    607.67183    0.64911
 6  b   1393.59414  1392.08203   1.51211        362.64433    363.03824   -0.39391
```

6	c	1573.22635	1571.05217	2.17419	321.23732	321.68188	-0.44456
7	a	828.65617	827.95417	0.70200	609.87781	610.39491	-0.51710
7	b	1380.80826	1381.93021	-1.12195	366.00231	365.70516	0.29715
7	c	1564.43592	1564.97280	-0.53688	323.04232	322.93150	0.11082
8	a	835.30852	835.95318	-0.64466	605.02078	604.55421	0.46657
8	b	1362.78221	1362.77540	0.00681	370.84356	370.84542	-0.00185
8	c	1552.08963	1552.19560	-0.10597	325.61200	325.58977	0.02223

Correlation coefficients:

		1	2	3
1:	R(16, 1)	1.000		
2:	R(19,14)	-0.136	1.000	
3:	R(22,19)	-0.892	-0.258	1.000

Principal coordinates and estimated uncertainties:

ATOM NO.	A	dA	B	dB	C	dC	MASS
1	-0.58333	0.00028	0.03985	0.00009	0.13698	0.00004	12.000000
2	-0.40977	0.00047	-0.04512	0.00198	1.51172	0.00006	15.994915
3	0.66529	0.00073	0.64970	0.00357	2.03582	0.00123	12.000000
4	-0.63615	0.00099	-1.41119	0.00066	-0.41447	0.00182	12.000000
5	-1.84829	0.00103	0.88169	0.00135	-0.13515	0.00088	12.000000
6	-2.95772	0.00043	0.31605	0.00170	0.35721	0.00082	18.998405
7	-1.70750	0.00196	2.11601	0.00054	0.42208	0.00241	18.998405
8	-2.03003	0.00158	1.06982	0.00315	-1.42941	0.00107	18.998405
9	-0.81222	0.00104	-1.40258	0.00247	-1.72148	0.00185	18.998405
10	0.53702	0.00155	-2.04331	0.00068	-0.17074	0.00233	18.998405
11	-1.60376	0.00170	-2.16612	0.00082	0.12232	0.00306	18.998405
12	0.24051	0.00084	0.57606	0.00024	-0.34344	0.00098	1.007825
13	0.89594	0.00135	1.58050	0.00309	1.50400	0.00245	1.007825
14	1.82390	0.00070	-0.16434	0.00448	1.97140	0.00090	18.998405
15	0.45939	0.00105	0.80398	0.00471	3.07253	0.00134	1.007825
16	1.53219	0.00446	2.24585	0.00616	-0.66351	0.00528	15.994915
17	1.23367	0.00424	2.93481	0.00548	-1.26580	0.00597	1.007825
18	2.42516	0.00438	1.97255	0.00636	-0.96529	0.00531	1.007825
19	3.51708	0.00186	-1.21346	0.00290	-0.08379	0.00044	15.994915
20	3.06600	0.00242	-1.94345	0.00195	-0.53107	0.00074	1.007825
21	2.92682	0.00167	-0.95607	0.00330	0.62732	0.00052	1.007825
22	3.92710	0.00311	1.16730	0.00821	-1.44177	0.00226	15.994915
23	4.78350	0.00304	1.53347	0.00770	-1.20169	0.00151	1.007825
24	3.86629	0.00326	0.27371	0.00707	-1.01642	0.00452	1.007825
		|		|		|	

NOTES: 1/ only the uncertainties for those coordinates which are completely defined by the fitted internals should be trusted
2/ the uncertainties are somewhat limited by the linear approximation coord=(d coord/d parameter)*parameter used for evaluation
3/ only the effect of the internals R, A, and D is propagated

---

Figure S1. NCI plot for the SEV-(H_2O) cluster.

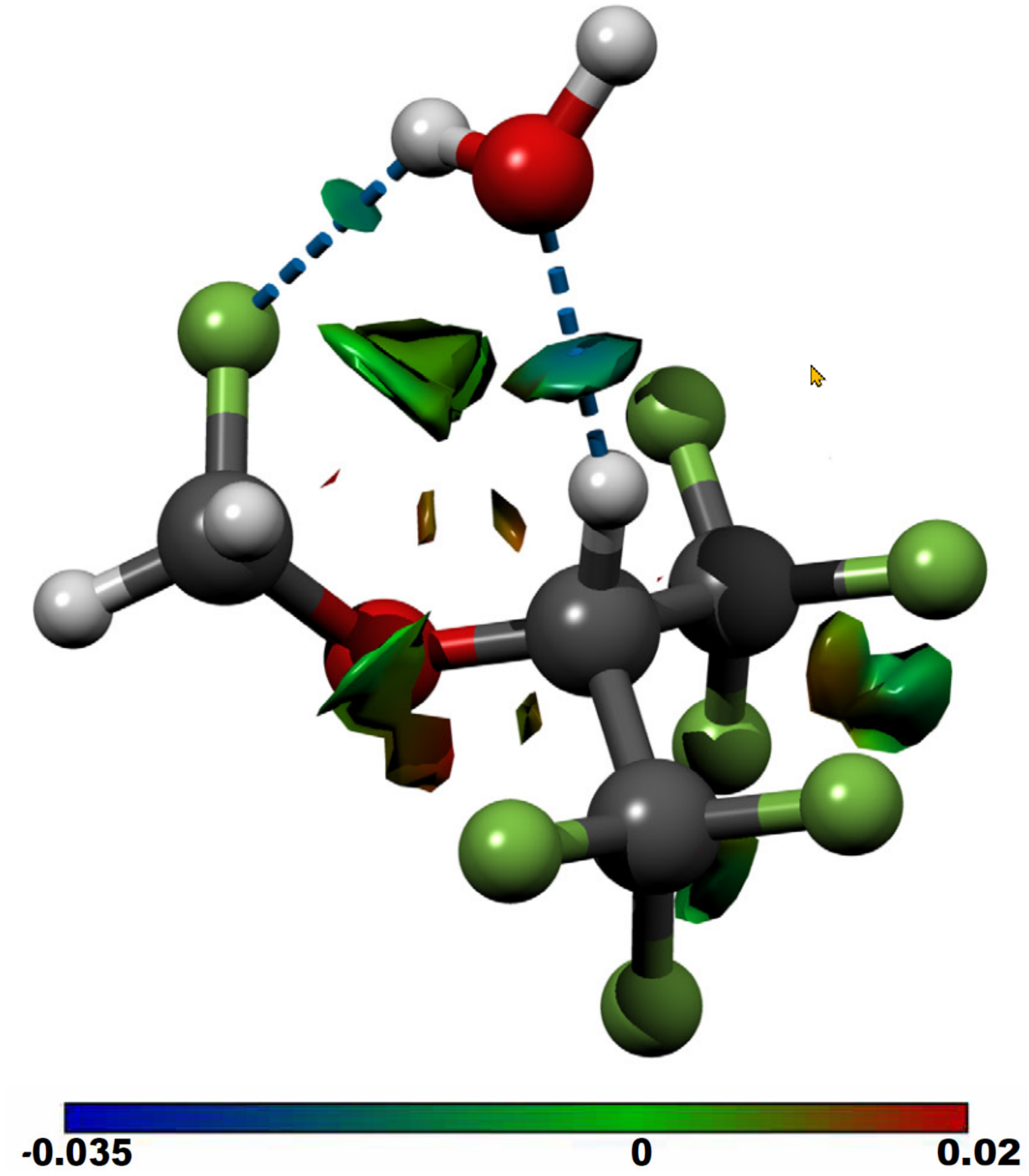

Figure S2. NCI plot for the SEV-$(H_2O)_2$ cluster.

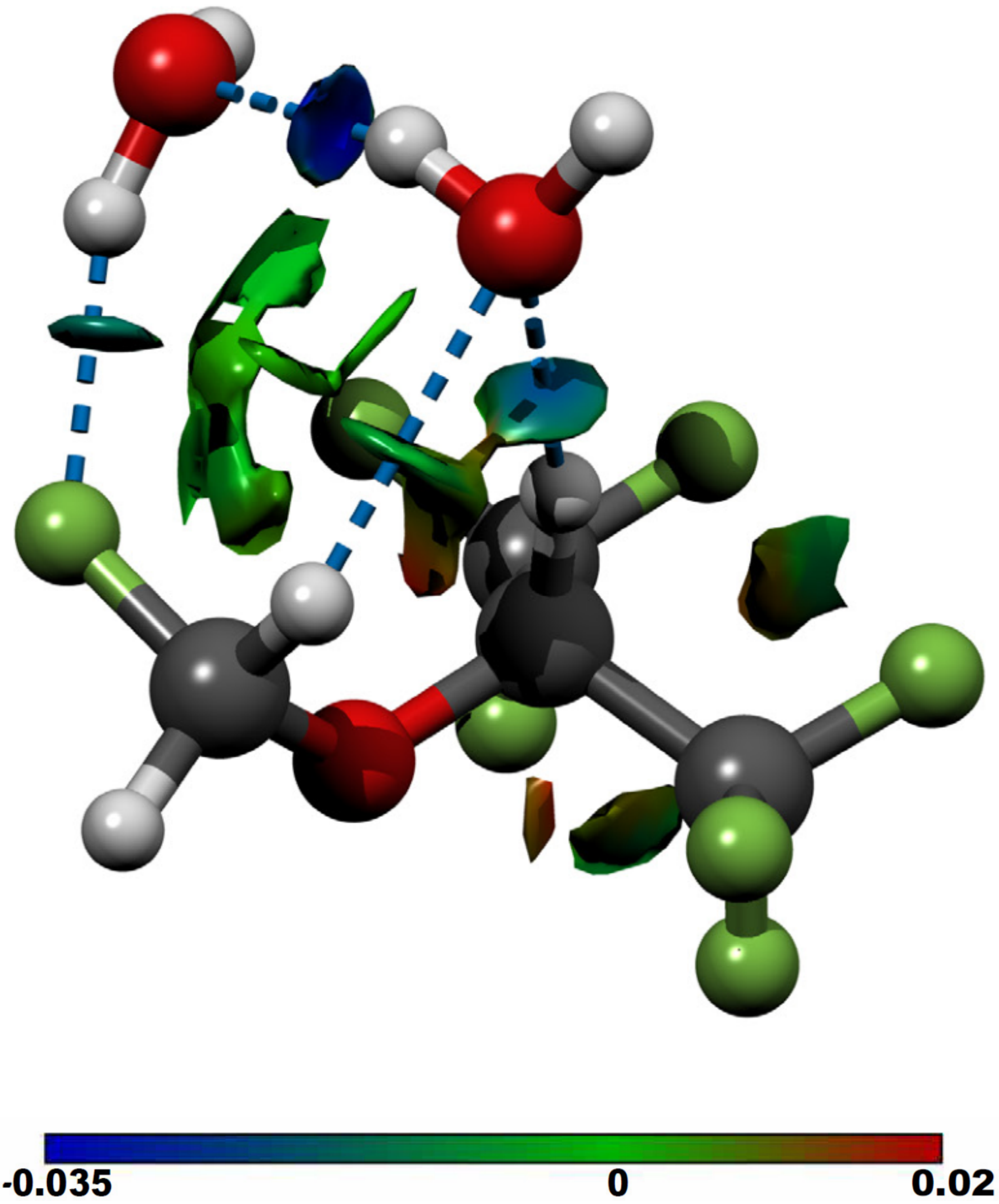

Figure S3. NCI plot for the SEV-$(H_2O)_3$ cluster.

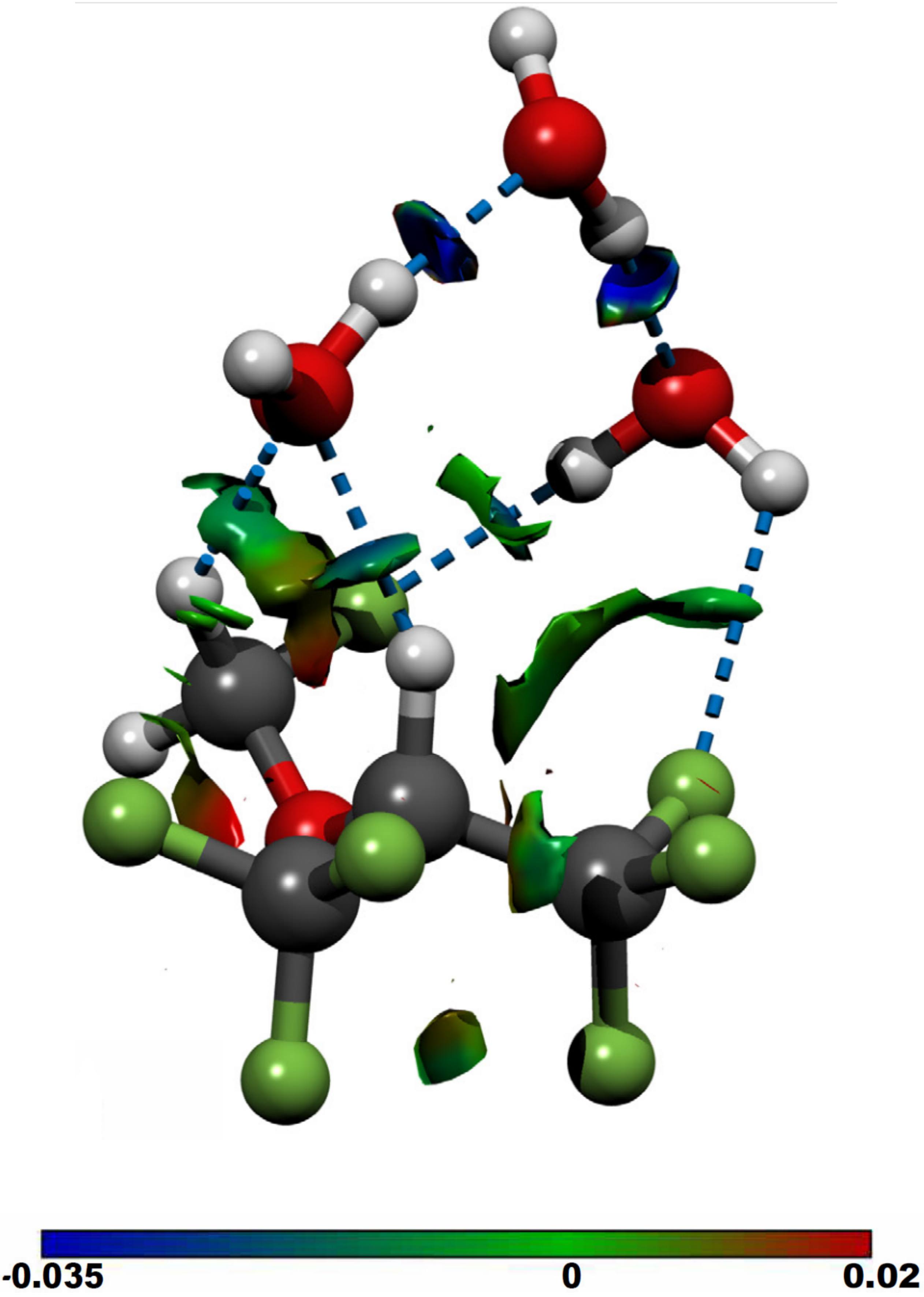

Figure S4. NCI plot for the SEV-$(H_2O)_4$ cluster.

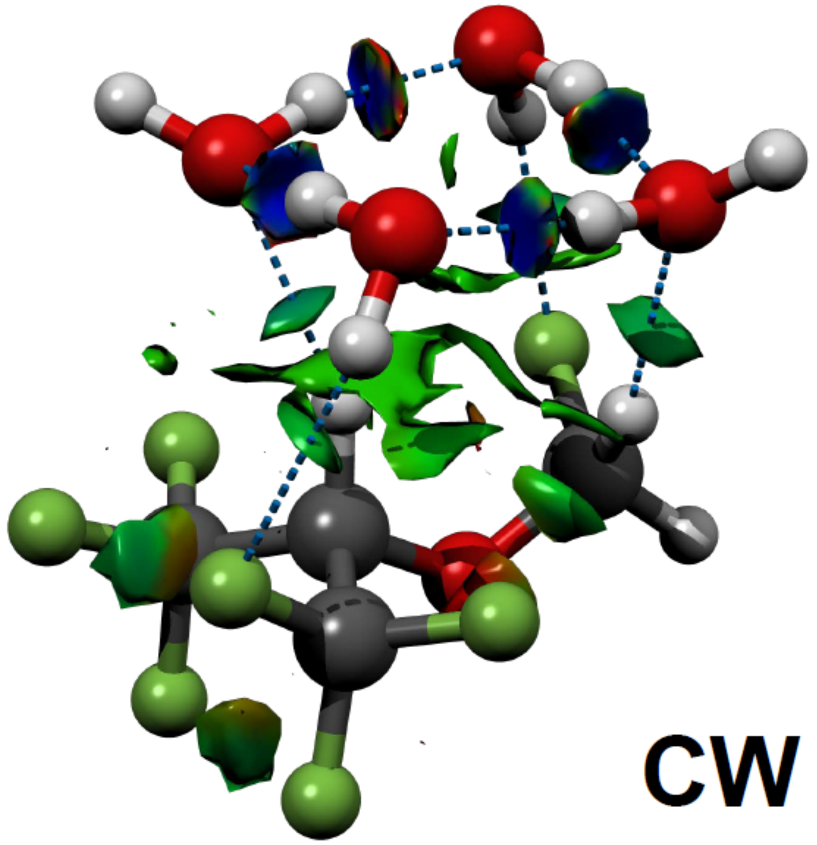

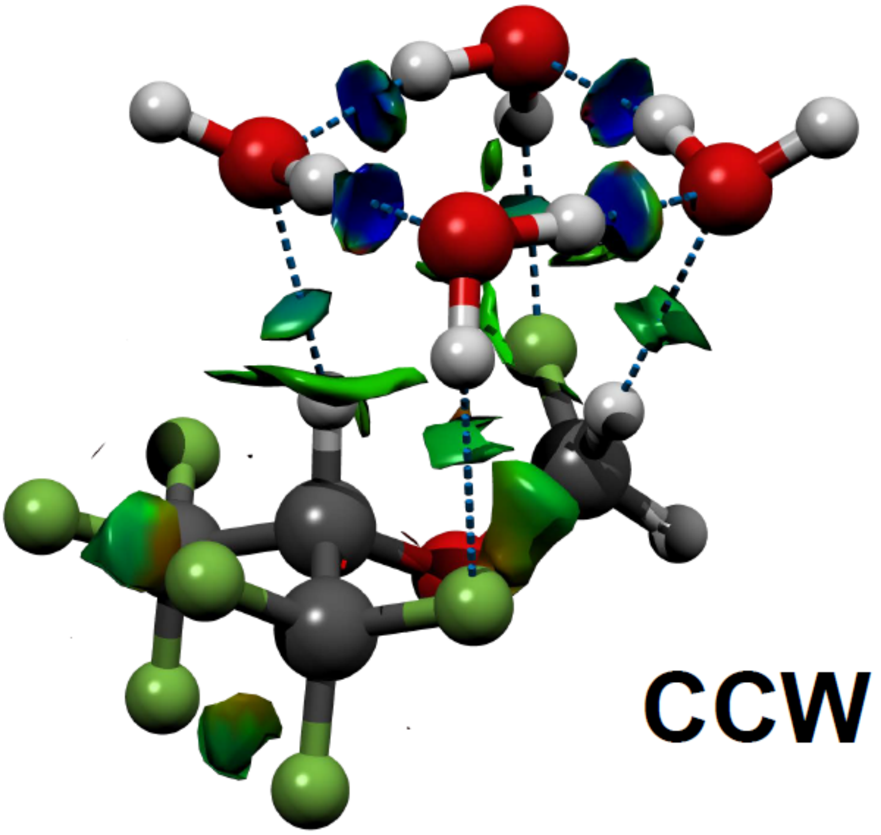

Supplement: Supplementary file 1 [file jz5c01767_si_001.pdf]
